# High correlation between Zika virus NS1 antibodies and neutralizing antibodies in selected serum samples from normal healthy Thais

**DOI:** 10.1038/s41598-019-49569-0

**Published:** 2019-09-18

**Authors:** Wannapa Sornjai, Suwipa Ramphan, Nitwara Wikan, Prasert Auewarakul, Duncan R. Smith

**Affiliations:** 1 Department of Microbiology, Faculty of Medicine, Siriraj Hospital, Mahidol University, Bangkok, Thailand; 2 Institute of Molecular Biosciences, Mahidol University, Salaya, Thailand

**Keywords:** Viral infection, Viral infection

## Abstract

Despite the widespread presence of the mosquito transmitted Zika virus (ZIKV) over much of Southeast Asia, the number of reported cases remains low. One possibility is that residents in Southeast Asia are immunologically protected, although the nature of any such protection remains unclear. This study sought to investigate the presence of antibodies directed to ZIKV NS1 protein in a selected sub-set of samples from a well characterized cohort of serum samples from normal, healthy Thais that had been previously characterized for the presence of neutralizing antibodies to ZIKV, DENV 1-4, and JEV. Because of similarities in molecular weight between the flavivirus E and NS1 proteins, an immunoblot system was established in which the NS1 antigen was not denatured, allowing detection of the dimer form of NS1, distinctly clear from the migration position of the E and NS1 monomer proteins. The results showed that antibodies to ZIKV NS1 protein were only detected in samples with ZIKV neutralizing antibodies (27/30 samples), and no sample (0/30) with a ZIKV plaque reduction neutralization test (PRNT)_90_ < 20 showed evidence of anti-ZIKV NS1 antibodies. The high correlation between the presence of ZIKV NS1 antibodies and ZIKV PRNT suggests that immunological protection against ZIKV infection in Thailand arises from prior exposure to ZIKV, and not through cross neutralization.

## Introduction

*Flaviviruses* are enveloped viruses with an approximately 11-kb positive sense single stranded RNA genome that contains one open reading frame encoding three structural proteins (C, prM and E) and 7 non-structural proteins (NS1, NS2A, NS2B, NS3, NS4A, NS4B and NS5)^1^. A number of mosquito transmitted human pathogenic viruses are members of the *Flavivirus* genus including dengue virus (DENV), Japanese encephalitis virus (JEV), yellow fever virus, West Nile virus, St. Louis encephalitis virus and Zika virus (ZIKV).

ZIKV was first isolated from a sentinel rhesus monkey in 1947 in Zika forest, Uganda, and was subsequently isolated from *Aedes africanus* mosquitoes the following year^2^. A second lineage of ZIKV, an Asian lineage, was discovered in *Aedes aegypti* mosquitoes in Malaysia in 1966^3^. Despite the apparent wide distribution of ZIKV in Africa and Asia only a handful of cases of human infection were reported prior to 2007 (as reviewed elsewhere^4^). The Asian lineage ZIKV emerged in Micronesia in 2007 and again in French Polynesia in 2013 from where the virus was subsequently transmitted to many countries around the world. However, while the introduction of ZIKV into many countries was followed by epidemic outbreaks, Southeast Asia has seen remarkably few cases (as reviewed elsewhere^5^). Clear evidence has shown that ZIKV has circulated in Thailand since as early as 2006^6^, but only around 2,000 cases of ZIKF have been reported in that time, with the majority being reported over the last 2 years^7^.

In a recent study we showed that a high proportion of normal healthy Thais have neutralizing antibodies to ZIKV, and that neutralization of ZIKV does not apparently arise from cross neutralization from prior DENV infection^8^. Neutralization arises through antibodies recognizing the virion and either blocking attachment or membrane fusion^9^, and while antibodies raised against the structural proteins is the main antigenic response to infection, antibodies are also generated against the flavivirus NS1 protein^10^.

NS1 is a non-structural glycoprotein that is highly conserved among flaviviruses. Its molecular weight ranges from 46–55 kDa depending on the glycosylation status^11^. This protein is initially translated as part of the viral polyprotein, and cleavage from the polyprotein gives rise to NS1 monomers in the ER lumen followed by N-linked glycosylation. NS1 forms homodimers associated with the ER-lumen and organelle membranes^11^, but additionally membrane bound NS1 is transported to the plasma membrane or fuses with the trans-Golgi network where it undergoes to maturation and modification and is released from infected cells as a soluble hexamer^12^. NS1 can be detected in patient serum^13–15^, which has been applied to diagnosis of DENV^16^ and ZIKV^17^ infection. Although the function of NS1 remains to be fully elucidated, evidence suggests that the NS1 dimer is required for viral replication while surface associated and secreted NS1 proteins are highly immunogenic and play an important role in immune invasive and pathogenesis by interacting with the host immune system during virus infection^11^.

To further understand the immune status of Thais with regards to ZIKV infection, this study sought to investigate the presence of ZIKV NS1 antibodies in serum samples from normal healthy Thais that had previously been extensively characterized for the presence or absence of ZIKV, DENV and JEV neutralizing antibodies^8^. Using a solid matrix western blotting system, anti-NS1 protein antibodies were investigated in 30 samples with no ZIKV neutralization, and 30 samples with high levels (plaque reduction neutralization test (PRNT_90_ ≥ 20) of ZIKV neutralization. Because of the well documented evidence of cross-neutralizing antibodies generated by *Flavivirus* infections^18^, we selected samples that either had no anti-ZIKV NS1 antibodies (namely PRNT_90_ < 20) or had a highly robust value for PRNT (namely PRNT_90_ ≥ 20). This means that a 20 fold dilution of human sera will reduce the input virus by 90% or greater. In contrast, many authors use a value of PRNT50 ≥ 10 (a tenfold dilution of serum neutralizes 50% of the input virus) for defining the presence of neutralizing antibodies^19–21^. However, the WHO criteria for DENV suggest that a value of PRNT90 ≥ 20 allows sufficient discrimination between specific neutralization and cross neutralization^22^. Significantly, in our study anti-ZIKV NS1 antibodies were only found in samples with high levels (PRNT90 ≥ 20) of ZIKV neutralizing antibodies, and anti-ZIKV NS1 antibodies were absent in samples with no ZIKV neutralizing antibodies.

## Materials and Methods

### Serum samples

The study was approved by the Mahidol University Central Institutional Review Board (Number: COA No. MU-CIRB 2017/067.2404) and was conducted in accordance with the ethical standards as laid down in the 1964 Declaration of Helsinki and its later amendments. Serum samples consisted of 60 serum samples obtained from healthy Thais, recruited through advertisement at the Institute of Molecular Biosciences, Mahidol University Thailand, after written informed consent and characterized for neutralizing antibodies to DENV 1-4, JEV and ZIKV as previously described^8^. Two additional control samples (one diagnosed ZIKV positive and five flavivirus naïve), again as previously described^8^ were included in this study. The sixty serum samples selected from the previous cohort^8^ consisted of 30 samples that were negative for ZIKV neutralizing antibodies (ZIKV PRNT_90_ < 20) and 30 samples that were positive for ZIKV neutralizing antibodies (ZIKV PRNT_90_ ≥ 20).

### Antigen production

Baby hamster kidney cells (BHK-21; ATCC Cat no. CCL-10) were seeded onto 10 cm^3^ dishes in Dulbecco’s Modified Eagle’s Medium (DMEM; Gibco, Invitrogen, Carlsbad, CA) supplemented with 10% heat inactivated fetal bovine serum (FBS; Gibco, Invitrogen) and 100 unit/mL of penicillin/streptomycin (Gibco, Invitrogen). Cells were grown at 37 °C with 5% CO_2_ for overnight. Confluent cells were infected with ZIKV (strain SV0010/15), JEV (strain Beijing-1), DENV 1 (strain 16007), DENV 2 (strain 16681), DENV 3 (strain 16562) and DENV 4 (strain 1036). Infected cells were maintained in Opti-MEM Reduced Serum Medium (Gibco, Invitrogen) at 37 °C with 5% CO_2_. BHK-21 cells incubated with virus free Opti-MEM was used as a negative control (mock). JEV supernatant was collected at 24 hours post infection (h.p.i) while DENV and ZIKV supernatants were collected at 48 h.p.i.

### Immunoblot analysis

Supernatants from viral infections or mock infections were mixed with 5X non-reducing loading dye (0.3 M Tris-HCl pH 6.8, 5% SDS, 50% glycerol, 0.015% bromophenol blue) and either boiled or not boiled before proteins were separated by electrophoresis through 10% SDS-polyacrylamide gels. Proteins were transferred onto 0.2 μm nitrocellulose membranes (GE Healthcare Life Sciences, Chicago, Il) and membranes were blocked with 5% skimmed milk in 1X TBS containing 0.5% tween for 30 min. For validation of NS1 protein expression, membranes were incubated either with a rabbit polyclonal anti-ZIKV NS1 antibody (GTX133307, GeneTex Inc., Irvine, CA), a rabbit polyclonal anti-ZIKV envelope protein antibody (GTX133326, GeneTex Inc.), a rabbit polyclonal anti-DENV 2 NS1 antibody (PA5-27885, Thermo Fisher Scientific, Rockford, IL), a pan specific anti-flavivirus envelope protein monoclonal antibody (purified in house from hybridoma HB112^23^) in 5% skimmed milk for overnight or an appropriate dilution of human serum in 5% skimmed milk for 1 h. The excess primary antibodies or human serum was removed and membranes were subsequently incubated with an appropriate secondary antibody, namely either with a HRP conjugated goat anti-rabbit IgG (31460, Thermo Fisher Scientific), a HRP conjugated goat anti-mouse IgG (A5278, Sigma Aldrich, Saint Louis, Missouri) or a HRP conjugated goat anti-human IgG (62–8420, Thermo Fisher Scientific) for 1 h. Immunochemiluminescent signal was developed by using Luminata Forte Western HRP substrate (Merck, Darmstadt, Germany) and exposure to x-ray film.

## Results

### Antigen evaluation

**Figure 1:**
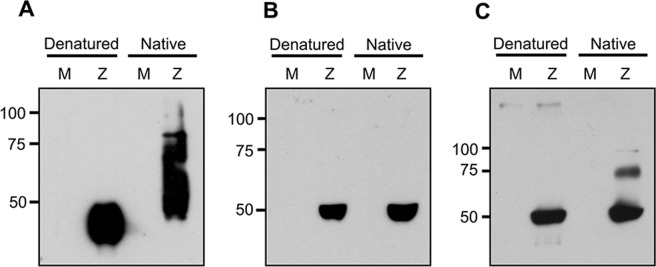
Immunoreactivity of ZIKV NS1 and envelope (E) proteins. Supernatant was collected from mock (M) and ZIKV infected (Z) BHK-21 cells. Proteins were separated by SDS-PAGE under either denaturing (boiled) or native (not boiled) conditions and transferred onto nitrocellulose membrane. Membrane was probed with (**A**) an anti-ZIKV NS1 polyclonal antibody, (**B**) an anti-ZIKV E polyclonal antibody, and (**C**) human ZIKV positive serum.

To detect the presence of NS1 antibodies in selected serum from normal healthy Thais with known ZIKV PRNT status, a western immunoblot system was established using supernatant from infected BHK-21 cells, and specificity was determined. Results (Fig. 1) showed that both ZIKV E and NS1 proteins were clearly detectable, but that both were migrating at approximately the same molecular weight (around 50 kDa). Human serum showed only one band in denatured samples, but two bands were clearly evident in non-denatured samples, with the lower band probably representing both E and NS1 proteins, while the upper band marked the position of NS1 dimers. Thus anti-NS1 antibodies can be specifically detected by the presence of the upper (dimeric) NS1 band.

**Figure 2:**
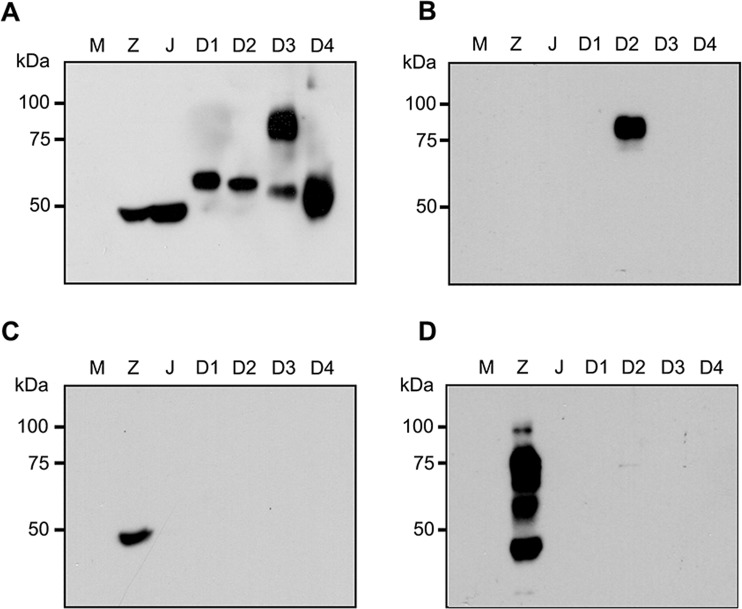
Characterization of envelope (E) and NS1 proteins. Supernatant was collected from mock (M) and flavivirus (ZIKV (Z), JEV (J) and DENV 1-4 (D1-D4)) infected BHK-21 cells. Proteins were separated by SDS-PAGE under native conditions and transferred onto a nitrocellulose membrane. Membrane was probed with (**A**) monoclonal antibody HB112, (**B**) an anti-DENV 2 NS1 polyclonal antibody, (**C**) an anti-ZIKV E polyclonal antibody and (**D**) an anti-ZIKV NS1 polyclonal antibody.

To confirm specificity of the immunoblots, supernatant from BHK-21 cells infected with ZIKV, JEV or DENV 1-4 as appropriate, in parallel with supernatants obtained from mock infected cells were again separated by electrophoresis and transferred to solid matrix support before incubation with a pan-flavivirus E protein antibody, an anti-DENV 2 NS1 antibody, an anti-ZIKV E antibody or an anti-ZIKV NS1 antibody. Results (Fig. 2) showed that E protein antigen was present in all lanes of virus infection (Fig. 2A) as detected by a pan-specific anti-flavivirus E protein antibody. We noted a band at approximately 75 kDa in the DENV 3 antigen lane (Fig. 2A) the nature of which is unclear. An antibody to DENV 2 NS1 protein showed no cross reaction with other NS1 proteins (Fig. 2B), similarly an anti-ZIKV NS1 protein antibody showed no cross reaction with other NS1 proteins (Fig. 2D).

**Figure 3:**
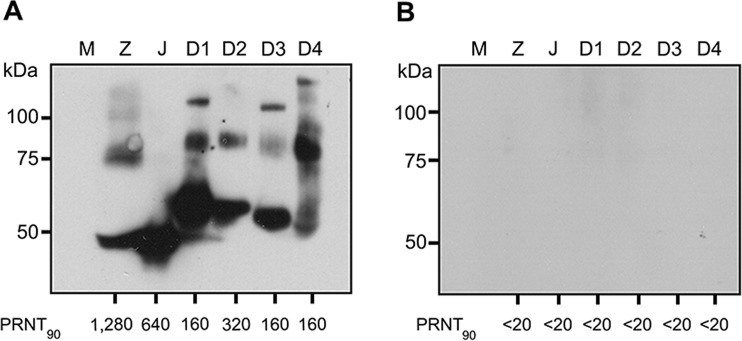
Characterization of envelope (E) and NS1 protein antibodies in flavivirus positive and negative control serum. Supernatant was collected from mock (M) and flavivirus (ZIKV (Z), JEV (J) and DENV 1-4 (D1-D4)) infected BHK-21 cells. Proteins were separated by SDS-PAGE under native conditions and transferred onto nitrocellulose membrane. Membrane was probed with (**A**) control human serum from a known case of ZIKV infection, and (**B**) control human serum known to be flavivirus naïve. PRNT_90_ values are as reported previously^8^.

We next evaluated the ability of this system to detect specific NS1 antibodies in two human serum control samples. As reported elsewhere^8^ these include a positive control serum from a diagnosed case of ZIKV infection (PRNT_90_ ZIKV:1,280; JEV: 640; DENV 1: 160; DENV 2: 320; DENV 3: 160; DENV 4: 160) and a negative control serum from a flavivirus naïve donor (PRNT_90_ ZIKV, JEV, DENV 1-4 all <20). Results (Fig. 3) showed close agreement with the PRNT data^8^. To confirm specificity a further 4 flavivirus naïve samples (PRNT_90_ ZIKV, JEV, DENV 1-4 all <20) were also screened (Supplemental Figs S57–S60 and Supplemental Table 1). NS1 antibodies, (as evidenced by detection of the dimer) were found for ZIKV and DENV 1-4 for the positive control serum sample, and no immunoreactivity at all was seen in the negative control samples. Interestingly no JEV NS1 antibodies were observed in the JEV positive control sample (Fig. 3A), although the serum showed a PRNT_90_ = 640^8^. However, Thailand has a national JEV vaccination campaign that was introduced in 1990, and the vaccine originally introduced into the national routine immunization schedule was an inactivated mouse brain vaccine^24^, and thus, given the age of the donor (22 years of age) the sera is consistent in showing the presence of neutralizing antibodies but no NS1 antibodies. In light of this there was complete correlation between the PRNT data and the immunoblot data, suggesting that this is a suitable system in which to investigate the presence of ZIKV NS1 antibodies in human sera.

### Presence of ZIKV NS1 antibodies in Thai sera

**Figure 4:**
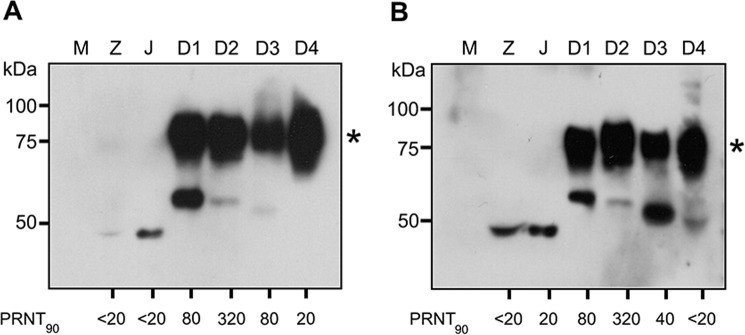
Representative immunoblots from ZIKV neutralizing antibody negative (ZIKV PRNT < 20) serum. Supernatant was collected from mock (M) and flavivirus (ZIKV (Z), JEV (J) and DENV 1-4 (D1-D4)) infected BHK-21 cells. Proteins were separated by SDS-PAGE under native conditions and transferred onto nitrocellulose membranes. Membranes were probed with ZIKV neutralizing antibody negative (ZIKV PRNT < 20) serum. Panel A shows the immunoblot of serum sample S6, while panel B shown the immunoblot for serum sample S60. The asterisk (*) marks the position of the NS1 dimer. PRNT_90_ values are as reported previously^8^. An additional 28 immunoblots can be found in the Supplemental materials file.

**Figure 5:**
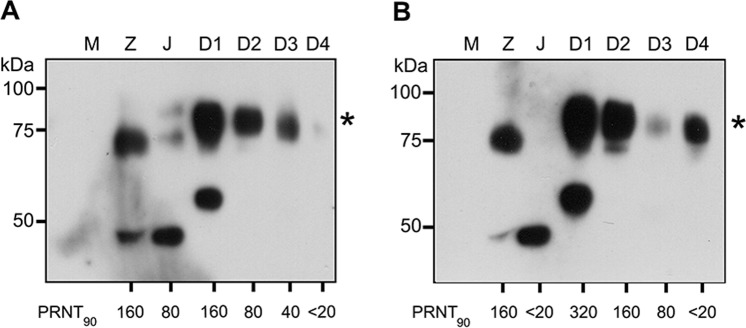
Representative immunoblots from ZIKV neutralizing antibodies positive (ZIKV PRNT ≥ 20) serum. Supernatant was collected from mock (M) and flavivirus (ZIKV (Z), JEV (J) and DENV 1-4 (D1-D4)) infected BHK-21 cells. Proteins were separated by SDS-PAGE under native condition and transferred onto nitrocellulose membrane. Membrane was probed with ZIKV neutralizing antibodies positive (ZIKV PRNT ≥ 20) serum. Panel A shows the immunoplot of serum sample S41, while panel B shown the immunoblot for serum sample S100. The asterisk (*) marks the position of the NS1 dimer. PRNT_90_ values are as reported previously^8^. An additional 28 immunoblots can be found in the Supplemental materials file.

To determine the presence of antibodies to ZIKV NS1 protein in Thai serum samples, a well characterized cohort of 60 samples were used. The PRNT_90_ data for ZIKV, JEV, DENV 1-4 have been previously reported^8^. The sixty samples consisted of 30 samples that were negative for ZIKV neutralizing antibodies (ZIKV PRNT_90_ < 20) and 30 samples that were positive for ZIKV neutralizing antibodies (ZIKV PRNT_90_ ≥ 20). All sera were screened against the full panel of antigens (ZIKV, JEV and DENV 1-4) with an appropriate negative control (supernatant from mock infected cells). The dilution of human serum used in the immunoblot was individually optimized per filter. Filters were scored on the presence of a NS1 dimer band to indicate the presence of NS1 antibodies. The results for the ZIKV PRNT_90_ negative (<20) samples are shown in Table 1, with representative immunoblots shown in Fig. 4, and the results for the PRNT_90_ positive (≥20) are shown in Table 2, with representative blots shown in Fig. 5. All immunoblots are shown in the Supplementary Figures and Table file.

**Table 1:**
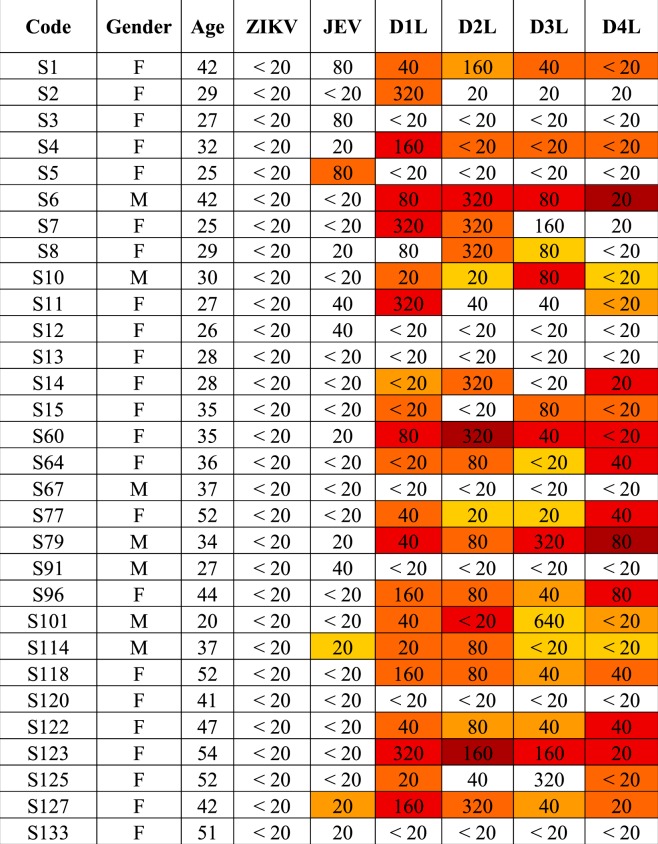
Summary of detection of NS1 antibodies and correlation with PRNT titer in ZIKV neutralizing antibody negative (PRNT_90_ < 20) serum samples.

Band intensities were quantitated using ImageJ software and signals are displayed as a heat map. White color represents no signal while yellow to dark red represent intensity from low to highest intensity. Comparisons are only valid within a serum sample. The numbers represent neutralizing antibody titer of each serum as determined by plaque reduction neutralization test as reported previously^8^. PRNT_90_ ≥ 20 was used as the cutoff.

**Table 2:**
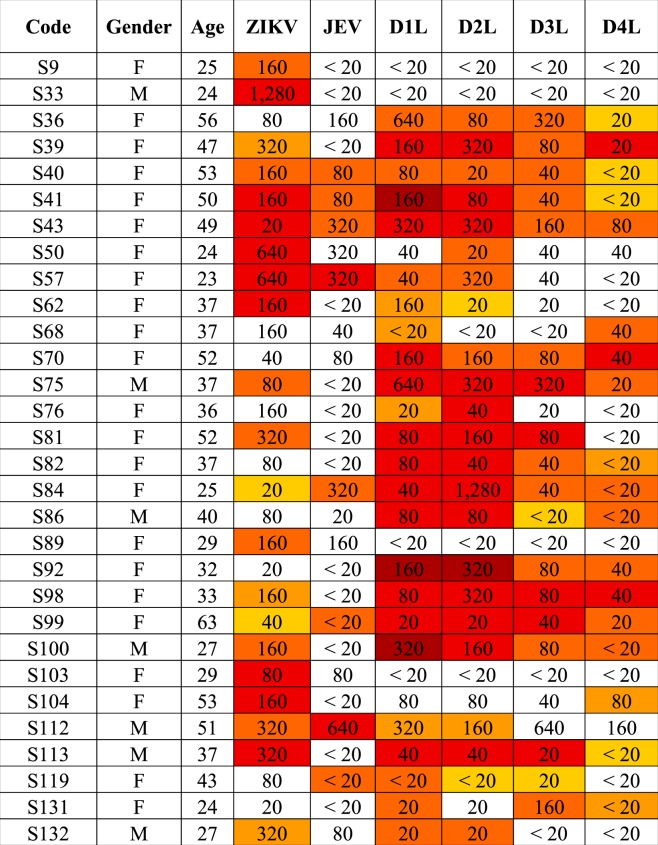
Summary of detection of NS1 antibodies and correlation with PRNT titer in ZIKV neutralizing antibody positive (PRNT_90_ ≥ 20) serum samples.

Band intensities were quantitated using ImageJ software and signals are displayed as a heat map. White color represents no signal while yellow to dark red represent intensity from low to highest intensity. Comparisons are only valid within a serum sample. The numbers represent neutralizing antibody titer of each serum as determined by plaque reduction neutralization test as reported previously^8^. PRNT_90_ ≥ 20 was used as the cutoff.

Most noticeably, no antibodies to ZIKV NS1 were seen in the samples screened to be negative for ZIKV neutralizing antibodies (ZIKV PRNT_90_ < 20). This is a 100% concordance between the PRNT data and the immunoblot data. ZIKV NS1 antibodies were detected in 21 of the 30 (70%) serum samples positive (ZIKV PRNT_90_ ≥ 20) for ZIKV neutralizing antibodies. Thus, the presence of antibodies to ZIKV NS1 is highly correlated with the presence of ZIKV neutralizing antibodies (*P* < 0.00001; Table 3). JEV showed the lowest association, with anti-JEV NS1 antibodies being seen in only 9/27 samples with a positive JEV PRNT (JEV PRNT_90_ ≥ 20), although the association was still statistically significant (*P* < 0.01; Table 3). Overall DENV concordance between the PRNT data and the NS1 antibody data varied widely (Table 3) most likely reflecting cross reactivity between antibodies directed against closely related DENV NS1 proteins.

**Table 3:** Association of anti-NS1 antibodies with the plaque reduction neutralization test*.

Virus	NS1 positive/PRNT_90_ ≥ 20	NS1 negative/PRNT_90_ < 20	*P*
ZIKV	21/30 (70%)	30/30 (100%)	<0.00001
JEV	9/27 (33.3%)	31/33 (93.9%)	<0.01
DENV 1	40/43 (93%)	12/17 (70.6%)	<0.00001
DENV 2	38/43 (88.4%)	14/17 (82.4%)	<0.00001
DENV 3	31/41 (75.6%)	15/19 (78.9%)	<0.0001
DENV 4	20/24 (83.3%)	19/36 (52.8%)	<0.01

*PRNT data as previously published^8^.

## Discussion

Zika virus is known to be widely distributed across Southeast Asia, with reports of recent infections in Southeast Asian countries including Thailand^25^, Vietnam^26^ and Myanmar^27^, but there have been no large scale outbreaks of ZIKF. This situation is in stark contrast to other countries, where the introduction of ZIKV was followed by large epidemic outbreaks^28^.

The history of the circulation of ZIKV in Southeast Asia remains unclear. While early serological studies suggested the circulation of ZIKV in Southeast Asia in the 1950s and early 1960s^29–32^, the reliability of some of this data has recently been questioned^7^. However, the isolation of ZIKV from *Aedes aegypti* mosquitoes in Malaysia^3^ definitively places ZIKV in Southeast Asia in the mid 1960s. The continued circulation of ZIKV in Southeast Asia can be inferred from a recent report of ZIKV isolated from a Thai specimen taken in 2006^6^ and the 2007 ZIKF outbreak in Yap State, Micronesia^33^ which was caused by an Asian lineage ZIKV. Three years later in 2010 ZIKV was detected in a patient in Cambodia^34^ and a retrospective analysis in Thailand identified cases of ZIKV infection occurring in 2012^35^. In the same year one case of ZIKF was identified in Philippines^36^. Thus, scattered cases of ZIKV infection have been reported over much of Southeast Asia for more than a decade. The significant question is therefore why ZIKV causes so few cases in Southeast Asia, while causing large epidemic outbreak when introduced to other countries and territories. Current prevailing theories include changes in the virus which alter transmissibility or possibly pathogenicity^37^, or that some degree of immune protection is present in the population of Southeast Asia^8^.

At least 4 mosquito transmitted flaviviruses circulate in Thailand, namely DENV^38^, JEV^39^, ZIKV^35^ and Tembusu virus (TMUV)^40^. While this latter virus is generally considered to be an avian specific *Flavivirus*^41^, high levels of human seroconversion have been reported in duck industry workers in China^42^ and this virus may play an as yet unrecognized role in Thailand. The antibody responses to these viruses can be highly cross reactive^18^, complicating the use of serology for sero-diagnosis^43^.

In our recent study utilizing serum samples from 135 normal healthy Thais we showed that neutralizing antibodies to ZIKV are present at levels that are considered to be protective (PRNT_50_ ≥ 10) in 70% of the samples, and that high levels of neutralizing antibodies (PRNT_90_ ≥ 20) were found in nearly one quarter of the samples examined^8^. Importantly, no association was found between the presence of neutralizing antibodies to other *Flaviviruses* and the presence of ZIKV neutralizing antibodies^8^. This is consistent with a study that showed cross protection against ZIKV by anti-DENV antibodies generally waned by 6 months post infection^44^. Combined, these studies suggest that there may have been significantly greater levels of ZIKV infection in Thailand than has been previously recognized, and indeed this has recently been confirmed^45^.

To further explore this issue, this study sought to look for antibodies to NS1 in the same serum samples previously characterized for neutralizing antibodies to ZIKV, JEV and DENV 1 to 4^8^. Because both the *Flavivirus* E protein and NS1 protein have similar molecular weights (approximately 50 and 46–50 kDa respectively) we employed a solid matrix immunoblot system in which the proteins were not denatured by boiling or disulphide bridge reduction, and looked for the presence of the NS1 dimer. Remarkably, ZIKV NS1 antibodies were only found in samples with ZIKV neutralizing antibodies. No reactivity to ZIKV NS1 was found in samples without ZIKV neutralizing antibodies. This result shows a lack of cross reactivity between anti-DENV NS1 antibodies and ZIKV NS1, as there was clear evidence of antibodies to DENV NS1 proteins in majority of serum samples examined.

ZIKV antibodies that recognize the ZIKV dimer NS1 antigen are composed of antibodies to both the monomer and any specific for the dimer form. Although not designed to distinguish between these antibodies, four patient samples (S39, S89, S99, S103; Supplementary file 1) reacted only with the dimer, with no evidence of interaction with the monomer suggesting that antibodies capable of recognizing the dimer, or possibly the hexamer of NS1 may form a part of the response to ZIKV infection. Similarly, in nine cases no evidence of anti-ZIKV NS1 antibodies was seen, despite ZIKV PRNT_90_ titers of ≥20. These cases could possibly indicate cross reaction from another flavivirus, and given the relatively apparent lack of cross reaction between ZIKV and DENV/JEV seen here, they might indicate the undetected circulation of yet another *Flavivirus*.

The specificity of the test used in this study was further confirmed by the very low correlation between JEV PRNT titers and the presence of anti-JEV NS1 antibodies. As noted, Thailand introduced a JEV vaccination campaign in the 1990s using an inactivated mouse brain purified virus^24^. As an inactivated virus vaccine, the immune response will be directed solely against structural proteins as no active replication (and hence no NS1 protein production) occurs. Interestingly however, some samples did show the presence of JEV NS1 specific antibodies, supporting the continued circulation of JEV in Thailand as has been reported by others^39^. However, two of the samples with anti-JEV NS1 antibodies were negative for JEV neutralizing antibodies, again possibly indicating the circulation of another unrecognized *Flavivirus*.

While studies have shown that the immune response to a flaviviral infection can last for many decades^46^, the length of time that antibodies to NS1 protein can be detected after infection remains poorly explored. One study has shown that anti-DENV NS1 antibodies can be detected for at least 3 years after infection^47^, while a second study has shown that the duration of seroconversion to seroreversion for JEV NS1 antibodies in subclinically infected humans is of the order of 4.2 years^48^. Given that this study looked at serum samples from normal healthy Thais, it suggests that anti-ZIKV antibodies may have a significantly longer time over which they may be detected.

Overall, this study shows the presence of anti-Zika virus antibodies in normal healthy Thai serum samples. Markedly, anti-ZIKV NS1 antibodies were found only in the serum samples able to neutralize ZIKV (PRNT_90_ ≥ 20), suggesting that these antibodies have arisen as a consequence of natural infection, rather than through cross reaction. In our original study, nearly 25% of samples had ZIKV PRNT_90_ ≥ 20^8^, again supporting that there has been significantly greater transmission of ZIKV in Thailand than has been previously thought. This could have occurred as a consequence of 80% of transmission being asymptomatic in humans^49^, as well as mild symptomatic cases being masked by the large number of cases of dengue fever occurring each year in Thailand^38^. Comparable studies in other countries in Southeast Asian countries may shed further light on the muted transmission of ZIKV in Southeast Asia.

## Supplementary information

Supplementary Figures and Table

Uncropped western blots

## Data Availability

All data generated or analysed during this study are included in this published article (and its Supplementary Information files).

## References

[1] Lindenbach BD, Rice CM (2003). Molecular biology of flaviviruses. Advance Virus Res.

[2] Dick G, Kitchen S, Haddow A (1952). Zika virus (I). Isolations and serological specificity. Trans. R. Soc. Trop. Med. Hyg..

[3] Marchette NJ, Garcia R, Rudnick A (1969). Isolation of Zika virus from Aedes aegypti mosquitoes in Malaysia. Am J Trop Med Hyg.

[4] Wikan N, Smith DR (2016). Zika virus: history of a newly emerging arbovirus. Lancet Infect. Dis..

[5] Duong V, Dussart P, Buchy P (2017). Zika virus in Asia. Int J Infect Dis.

[6] Nitatpattana, N. *et al*. Complete Genome Sequence of a Zika Virus Strain Isolated from the Serum of an Infected Patient in Thailand in 2006. *Genome Announc* **6**, 10.1128/genomeA.00121-18 (2018). 10.1128/genomeA.00121-18 PMC5843728 29519832

[7] Khongwichit Sarawut, Wikan Nitwara, Auewarakul Prasert, Smith Duncan R. (2018). Zika virus in Thailand. Microbes and Infection.

[8] Sornjai W, Jaratsittisin J, Auewarakul P, Wikan N, Smith DR (2018). Analysis of Zika virus neutralizing antibodies in normal healthy Thais. Scientific Rep.

[9] Dai L (2016). Molecular basis of antibody-mediated neutralization and protection against flavivirus. IUBMB Life.

[10] Edeling MA, Diamond MS, Fremont DH (2014). Structural basis of Flavivirus NS1 assembly and antibody recognition. Proc Natl Acad Sci USA.

[11] Muller DA, Young PR (2013). The flavivirus NS1 protein: Molecular and structural biology, immunology, role in pathogenesis and application as a diagnostic biomarker. Antiviral Res.

[12] Flamand M (1999). Dengue Virus Type 1 Nonstructural Glycoprotein NS1 Is Secreted from Mammalian Cells as a Soluble Hexamer in a Glycosylation-Dependent Fashion. J Virol.

[13] Young PR, Hilditch PA, Bletchly C, Halloran W (2000). An Antigen Capture Enzyme-Linked Immunosorbent Assay Reveals High Levels of the Dengue Virus Protein NS1 in the Sera of Infected Patients. J. Clin. Microbiol..

[14] Alcon S (2002). Enzyme-Linked Immunosorbent Assay Specific to Dengue Virus Type 1 Nonstructural Protein NS1 Reveals Circulation of the Antigen in the Blood during the Acute Phase of Disease in Patients Experiencing Primary or Secondary Infections. J. Clin. Microbiol..

[15] Macdonald J (2005). NS1 Protein Secretion during the Acute Phase of West Nile Virus Infection. J. Virol..

[16] Pal S (2014). Evaluation of dengue NS1 antigen rapid tests and ELISA kits using clinical samples. PLoS One.

[17] Jaaskelainen AJ (2019). Validation of serological and molecular methods for diagnosis of zika virus infections. J Virol Methods.

[18] Calisher CH (1989). Antigenic relationships between flaviviruses as determined by cross-neutralization tests with polyclonal antisera. J Gen Virol.

[19] Fortuna C (2017). Imported arboviral infections in Italy, July 2014-October 2015: a National Reference Laboratory report. BMC Infect Dis..

[20] Kim YH (2018). Development of a Rapid Diagnostic Test Kit to Detect IgG/IgM Antibody against Zika Virus Using Monoclonal Antibodies to the Envelope and Non-structural Protein 1 of the Virus. Korean J Parasitol..

[21] Lindholm DA (2017). Mosquito Exposure and Chikungunya and Dengue Infection Among Travelers During the Chikungunya Outbreak in the Americas. Am J Trop Med Hyg..

[22] WHO. Guidelines for plaque reduction neutralization testing of human antibodies to dengue virus. Geneva, Switzerland. http://apps.who.int/iris/bitstream/10665/69687/1/who_ivb_07.07_eng.pdf (2007). 10.1089/vim.2008.0007 18476771

[23] Henchal EA, Gentry MK, McCown JM, Brandt WE (1982). Dengue virus-specific and flavivirus group determinants identified with monoclonal antibodies by indirect immunofluorescence. Am J Trop Med Hyg.

[24] Pongpairoj S (1989). A test production of inactivated mouse brain JE vaccine in Thailand. Southeast Asian J Trop Me and Public Health.

[25] Wongsurawat Thidathip, Athipanyasilp Niracha, Jenjaroenpun Piroon, Jun Se-Ran, Kaewnapan Bualan, Wassenaar Trudy M., Leelahakorn Nattawat, Angkasekwinai Nasikarn, Kantakamalakul Wannee, Ussery David W., Sutthent Ruengpung, Nookaew Intawat, Horthongkham Navin (2018). Case of Microcephaly after Congenital Infection with Asian Lineage Zika Virus, Thailand. Emerging Infectious Diseases.

[26] Lan, P. T. *et al*. Fetal Zika Virus Infection in Vietnam. *PLoS Curr* **9**, 10.1371/currents.outbreaks.1c8f631e0ef8cd7777d639eba48647fa (2017). 10.1371/currents.outbreaks.1c8f631e0ef8cd7777d639eba48647fa PMC5693598 29188136

[27] Ngwe Tun Mya Myat, Kyaw Aung Kyaw, Hmone Saw Wut, Inoue Shingo, Buerano Corazon C., Soe Aung Min, Moi Meng Ling, Hayasaka Daisuke, Thu Hlaing Myat, Hasebe Futoshi, Thant Kyaw Zin, Morita Kouichi (2018). Detection of Zika Virus Infection in Myanmar. The American Journal of Tropical Medicine and Hygiene.

[28] Baud D, Gubler DJ, Schaub B, Lanteri MC, Musso D (2017). An update on Zika virus infection. Lancet.

[29] Hammon WM, Schrack WD, Sather GE (1958). Serological survey for a arthropod-borne virus infections in the Philippines. Am J Trop Med Hyg.

[30] Olson JG (1983). A survey for arboviral antibodies in sera of humans and animals in Lombok, Republic of Indonesia. Ann Trop Med Parasitol.

[31] Pond WL (1963). Arthropod-Borne Virus Antibodies in Sera from Residents of South-East Asia. Trans R Soc Trop Med Hyg.

[32] Smithburn KC (1954). Neutralizing antibodies against arthropod-borne viruses in the sera of long-time residents of Malaya and Borneo. Am J Hyg.

[33] Lanciotti RS (2008). Genetic and serologic properties of Zika virus associated with an epidemic, Yap State, Micronesia, 2007. Emerg Infect Dis.

[34] Heang V (2012). Zika virus infection, Cambodia, 2010. Emerg Infect Dis.

[35] Buathong R (2015). Detection of Zika Virus Infection in Thailand, 2012–2014. Am J Trop Med Hyg.

[36] Alera MT (2015). Zika virus infection, Philippines, 2012. Emerg Infect Dis.

[37] Liu Y (2017). Evolutionary enhancement of Zika virus infectivity in Aedes aegypti mosquitoes. Nature.

[38] Polwiang S (2016). Estimation of dengue infection for travelers in Thailand. Travel Med Infect Dis.

[39] Olsen SJ (2010). Japanese encephalitis virus remains an important cause of encephalitis in Thailand. Int J Infect Dis.

[40] O’Guinn ML (2013). Field detection of Tembusu virus in western Thailand by rt-PCR and vector competence determination of select culex mosquitoes for transmission of the virus. Am J Trop Med Hyg.

[41] Zhang W, Chen S, Mahalingam S, Wang M, Cheng A (2017). An updated review of avian-origin Tembusu virus: a newly emerging avian Flavivirus. J Gen Virol.

[42] Tang Y (2013). Tembusu virus in human, China. Transbound Emerg Dis.

[43] Allwinn R, Doerr HW, Emmerich P, Schmitz H, Preiser W (2002). Cross-reactivity in flavivirus serology: new implications of an old finding?. Med Microbiol Immunol.

[44] Montoya Magelda, Collins Matthew, Dejnirattisai Wanwisa, Katzelnick Leah C, Puerta-Guardo Henry, Jadi Ramesh, Schildhauer Samuel, Supasa Piyada, Vasanawathana Sirijitt, Malasit Prida, Mongkolsapaya Juthathip, de Silva Aruna D, Tissera Hasitha, Balmaseda Angel, Screaton Gavin, de Silva Aravinda M, Harris Eva (2018). Longitudinal Analysis of Antibody Cross-neutralization Following Zika Virus and Dengue Virus Infection in Asia and the Americas. The Journal of Infectious Diseases.

[45] Ruchusatsawat Kriangsak, Wongjaroen Pattara, Posanacharoen Arisara, Rodriguez-Barraquer Isabel, Sangkitporn Somchai, Cummings Derek A T, Salje Henrik (2019). Long-term circulation of Zika virus in Thailand: an observational study. The Lancet Infectious Diseases.

[46] Imrie A (2007). Antibody to dengue 1 detected more than 60 years after infection. Viral Immunol.

[47] Nascimento EJM (2018). Development of antibody biomarkers of long term and recent dengue virus infections. J Virol Methods.

[48] Konishi E, Kitai Y (2009). Detection by ELISA of antibodies to Japanese encephalitis virus nonstructural 1 protein induced in subclinically infected humans. Vaccine.

[49] Musso D, Gubler DJ (2016). Zika Virus. Clin Microbiol Rev.

