# Supplementary material for: High correlation between Zika virus NS1 antibodies and neutralizing antibodies in selected serum samples from normal healthy Thais
Source: Sci Rep. 2019 Sep 18;9:13498. doi: 10.1038/s41598-019-49569-0 (PMC6751300; doi:10.1038/s41598-019-49569-0)
Supplement: Supplementary file 1 — Supplementary Figures and Table [file 41598_2019_49569_MOESM1_ESM.pdf]

Supplemental data

Figure S1-S28 Detection of anti-ZIKV NS1 dimer antibodies in ZIVK $PRNT_{90} < 20$ human serum. All stars represent NS1 dimer.

S1

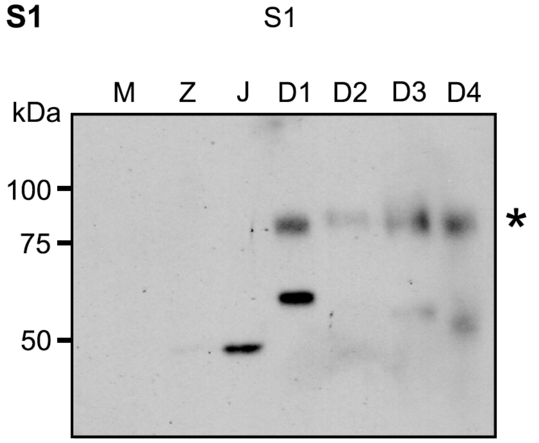

S2

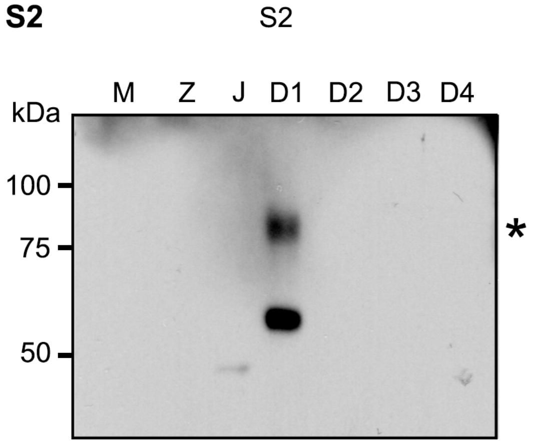

S3

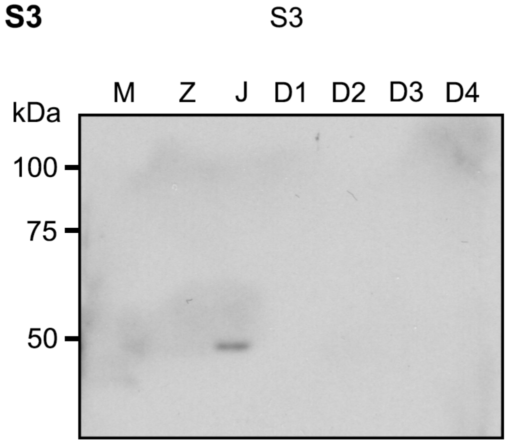

S4

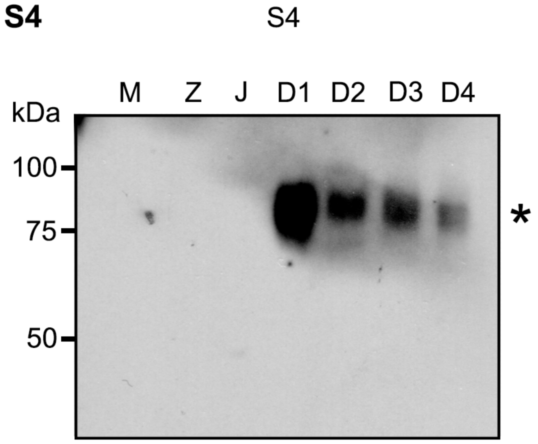

S5

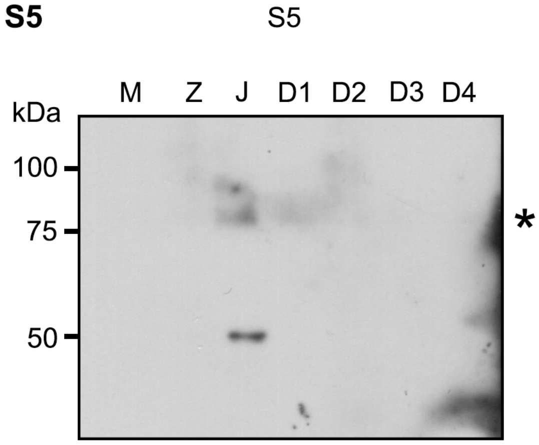

S6

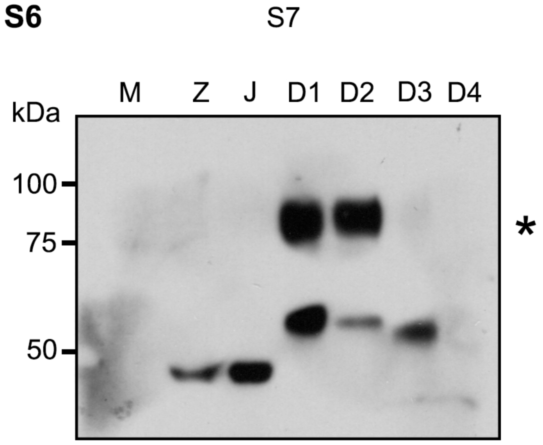

S7 S8

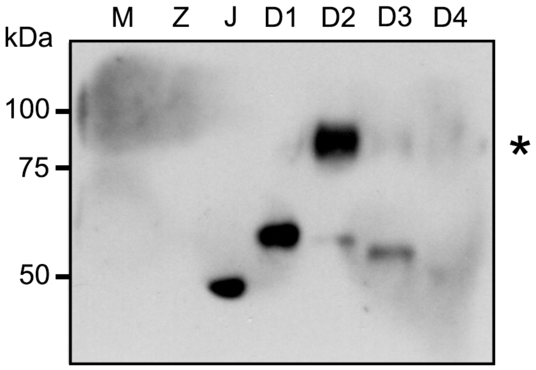

S8 S10

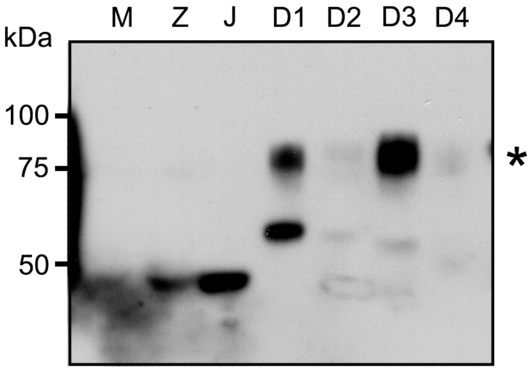

S9 S11

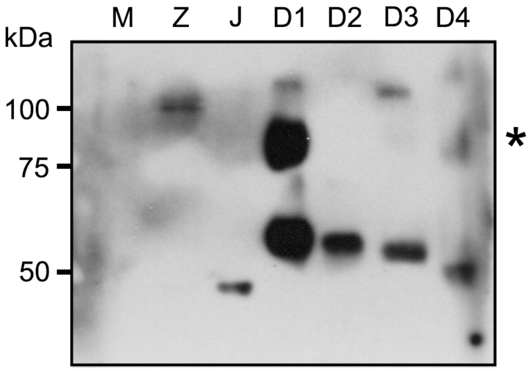

S10 S12

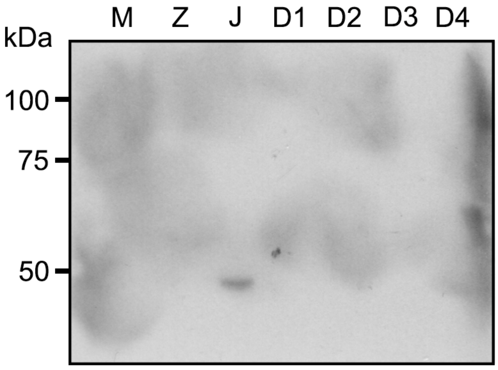

S11 S13

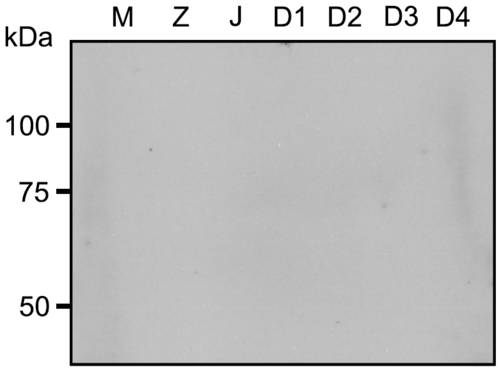

S12 S14

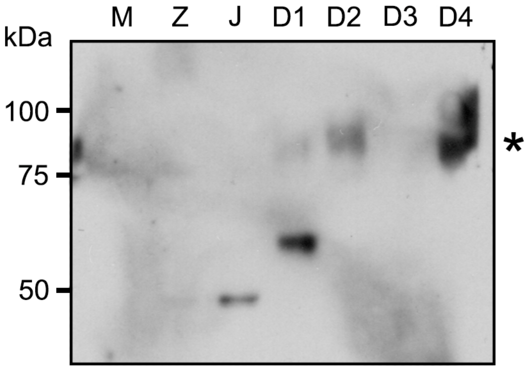

S13 S15

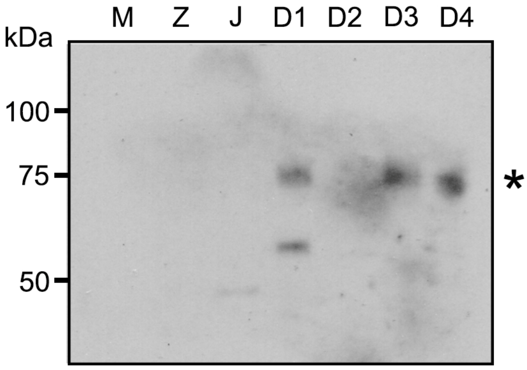

S14 S64

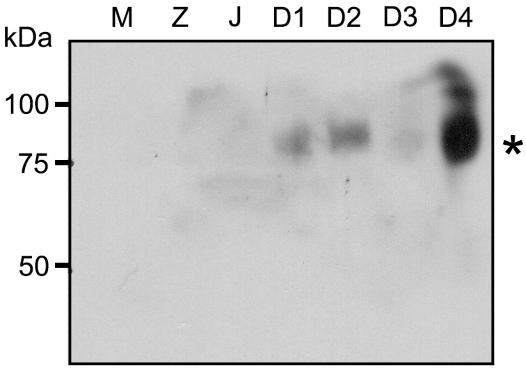

S15 S67

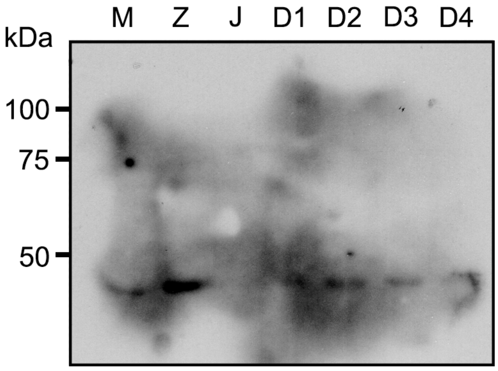

S16 S77

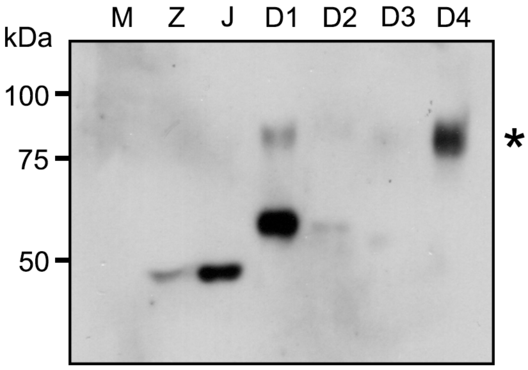

S17 S79

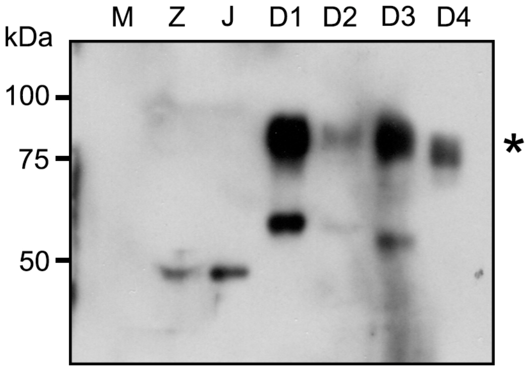

S18 S91

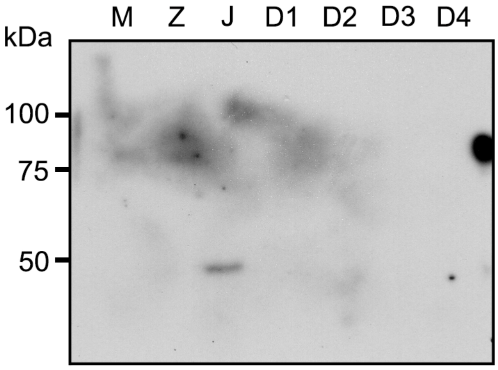

S19 S96

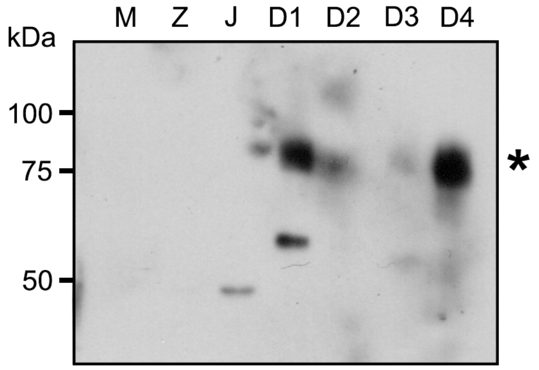

S20 S101

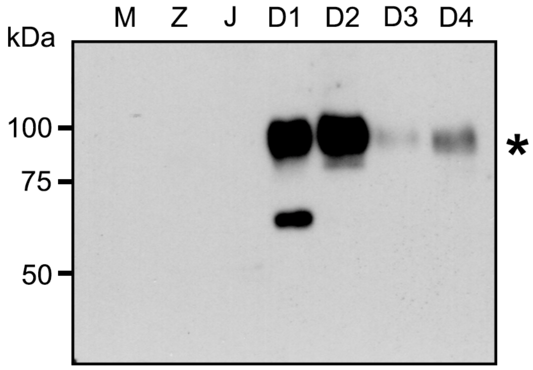

S21 S114

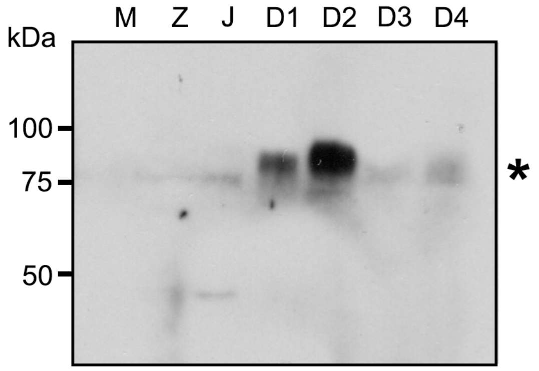

S22 S118

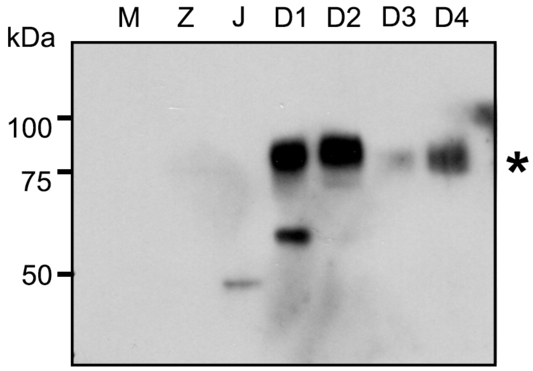

S23 S120

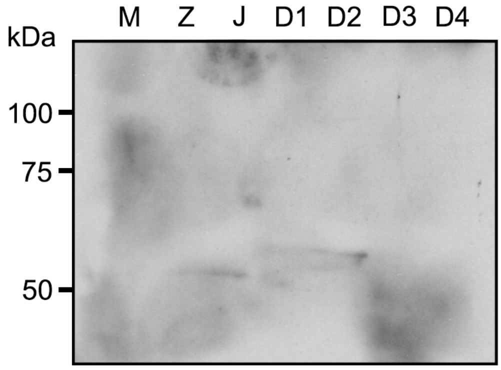

S24 S122

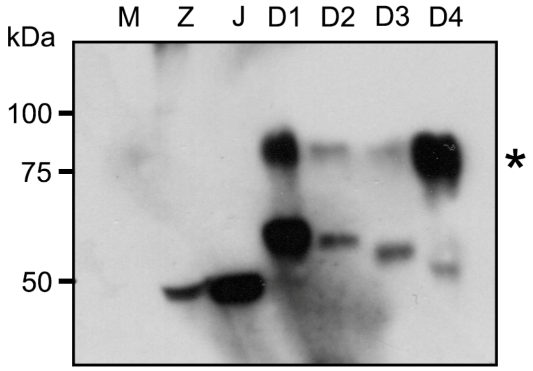

S25 S123

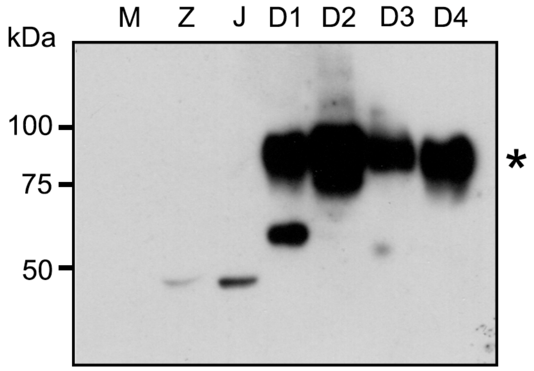

S26 S125

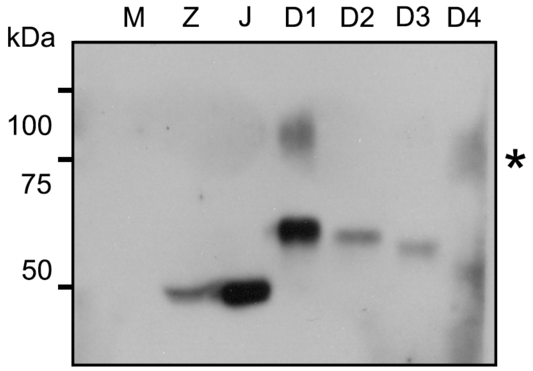

S27 S127

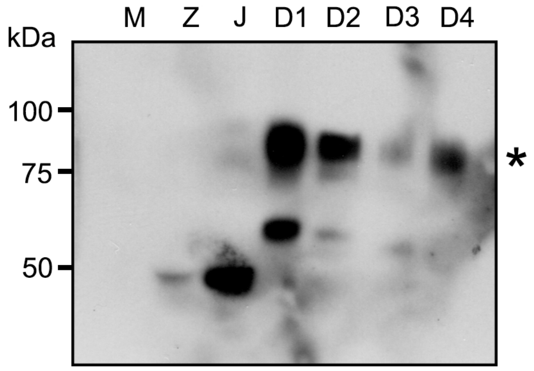

S28 S133

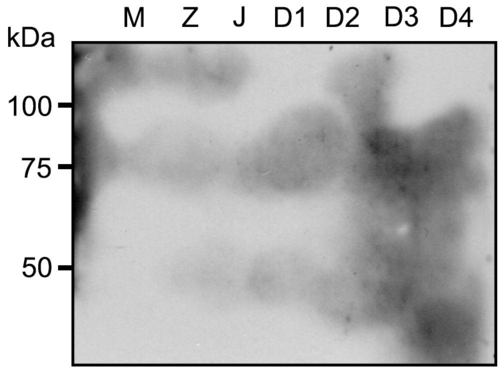

Figure S29-S56 Detection of anti-ZIKV NS1 dimer antibodies in ZIVK $PRNT_{90} \geq 20$ human serum. All stars represent NS1 dimer.

S29 S9

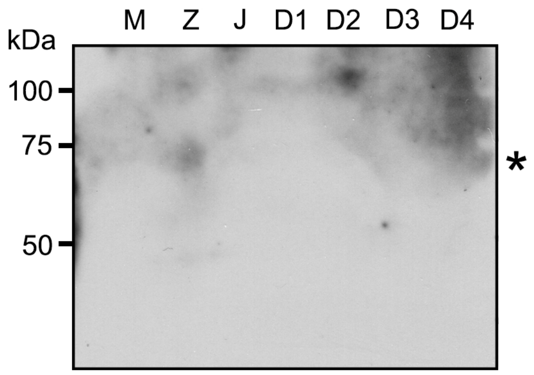

S30 S33

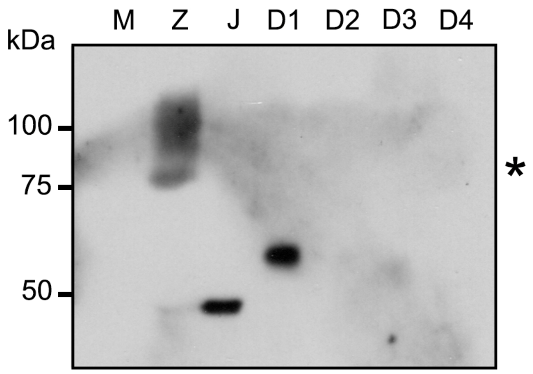

S31 S36

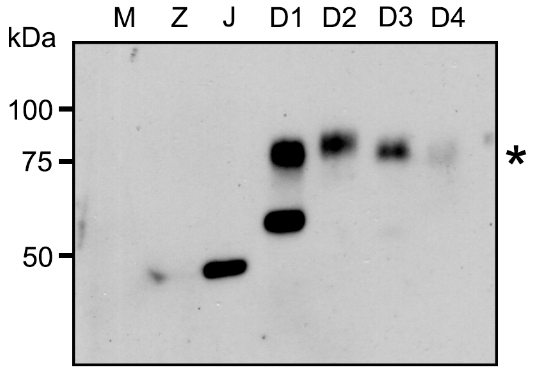

S32 S39

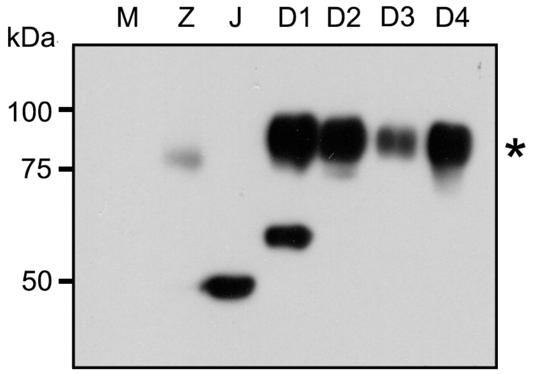

S33 S40

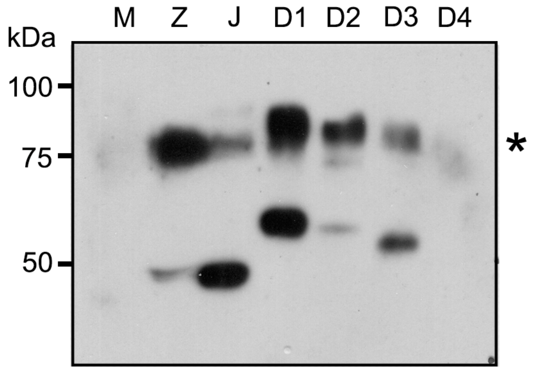

S34 S43

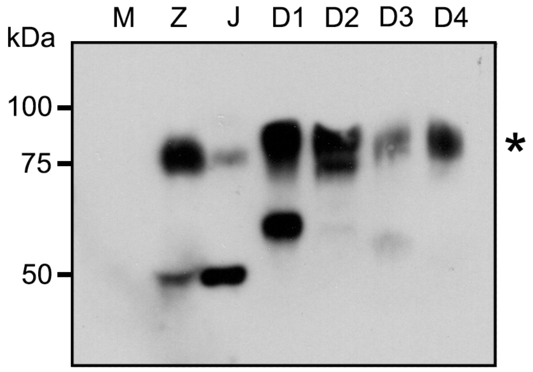

S35 S50

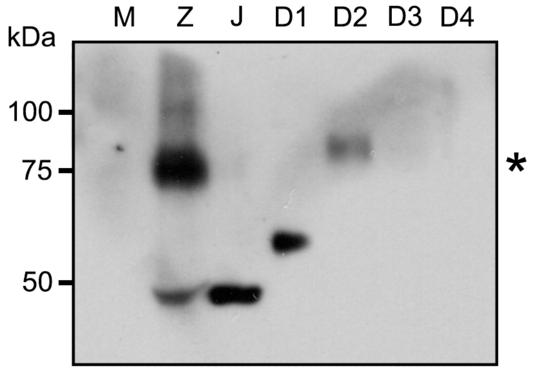

S36 S57

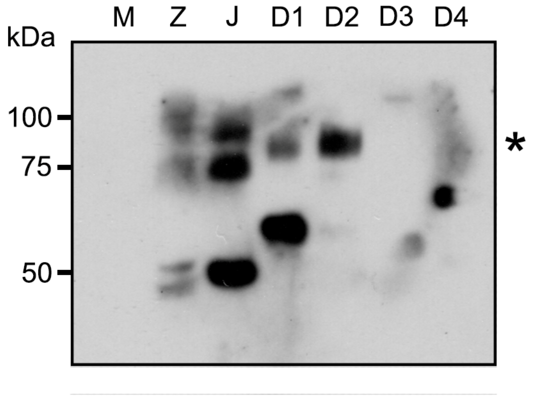

S37 S62

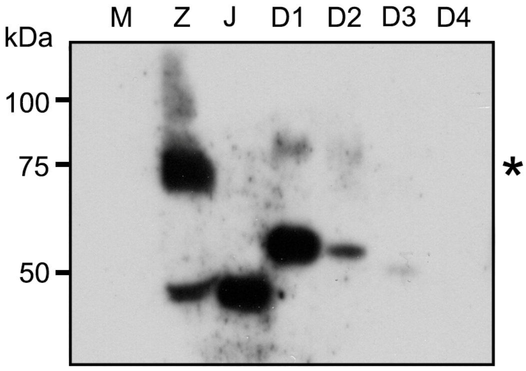

S38 S68

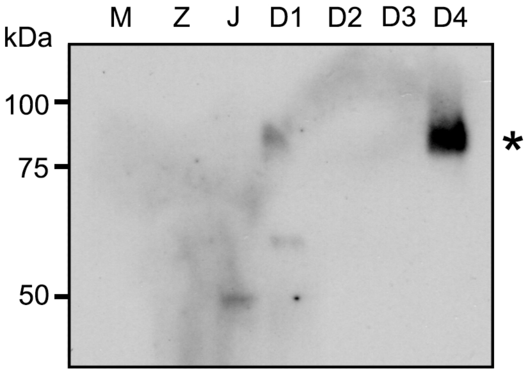

S39 S70

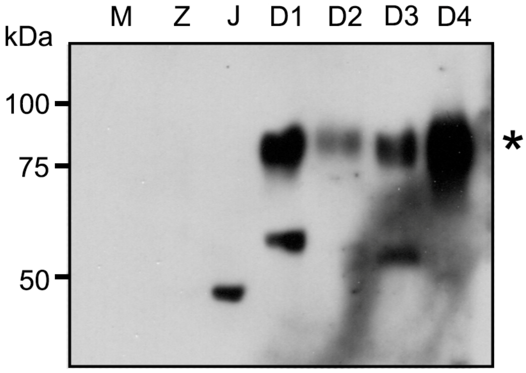

S40 S75

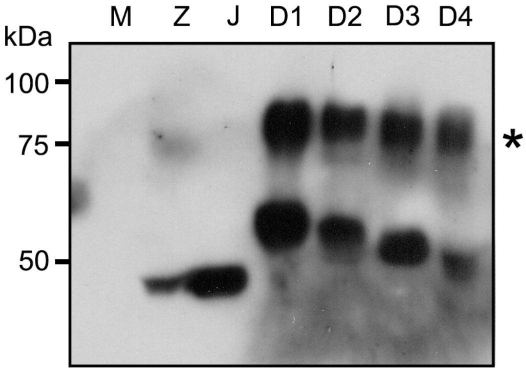

S41 S76

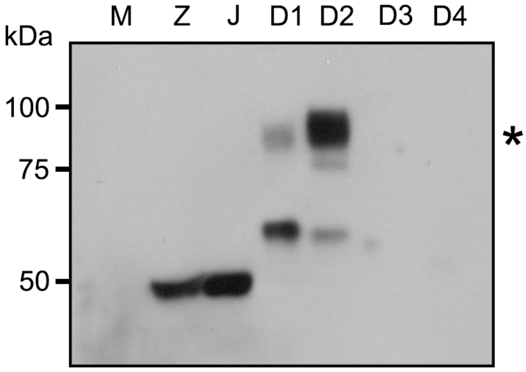

S42 S81

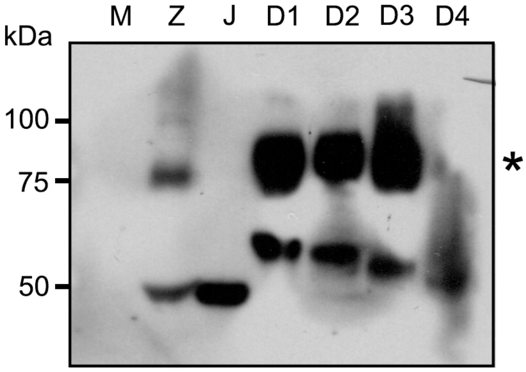

S43 S82

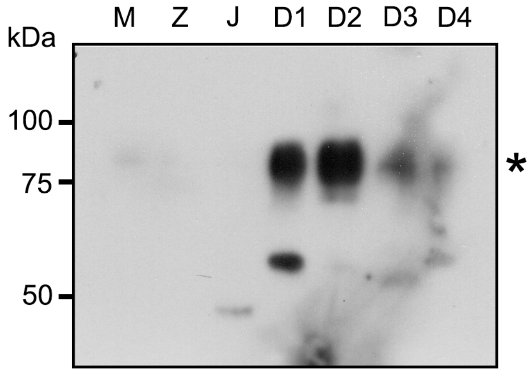

S44 S84

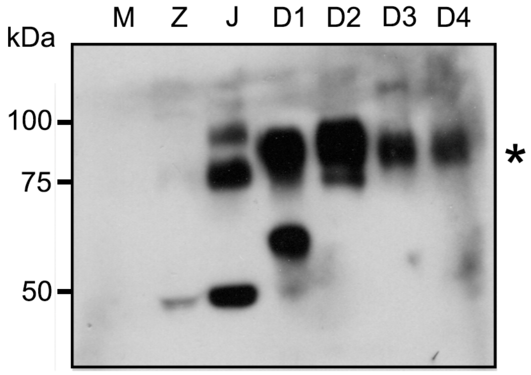

S45 S86

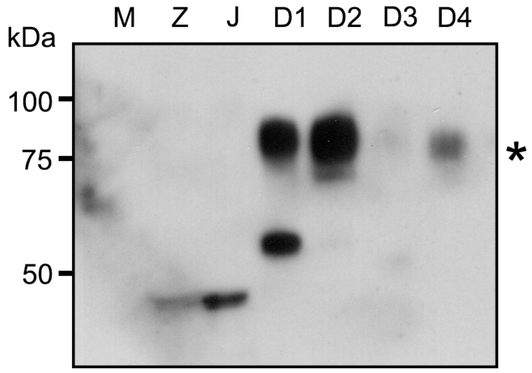

S46 S89

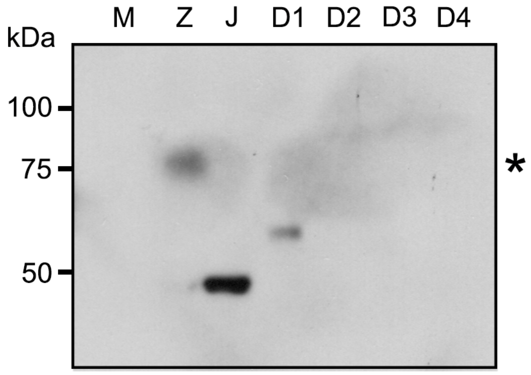

S47 S92

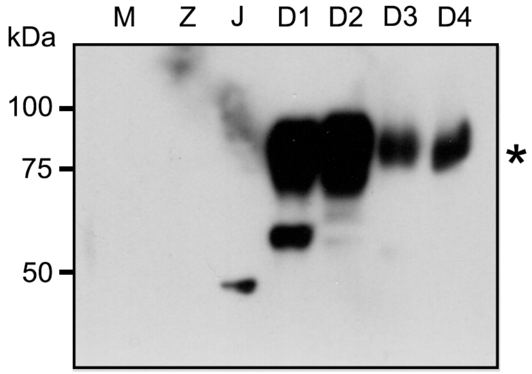

S48 S98

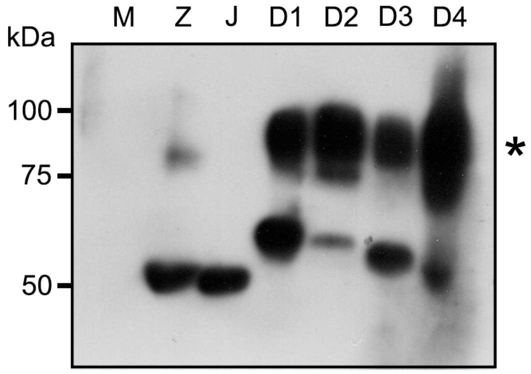

S49 S99

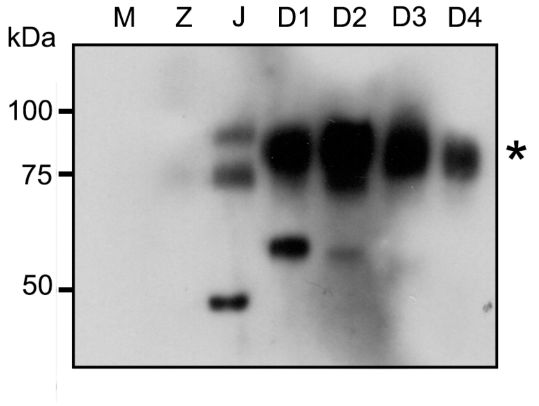

S50 S103

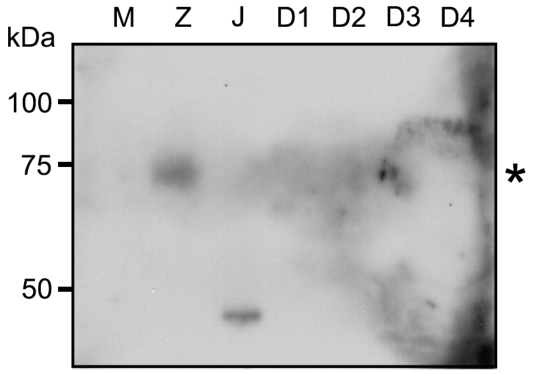

S51 S104

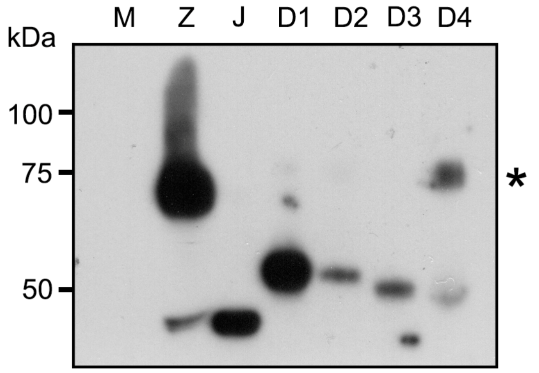

S52 S112

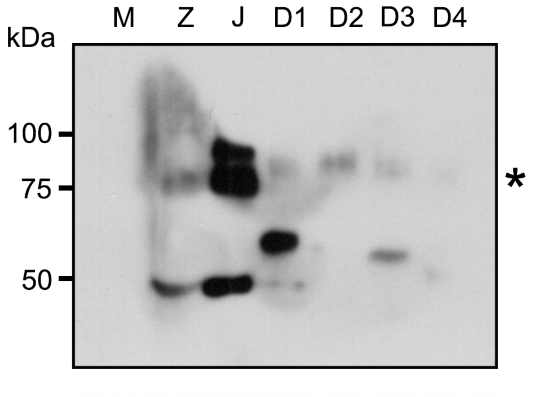

S53 S113

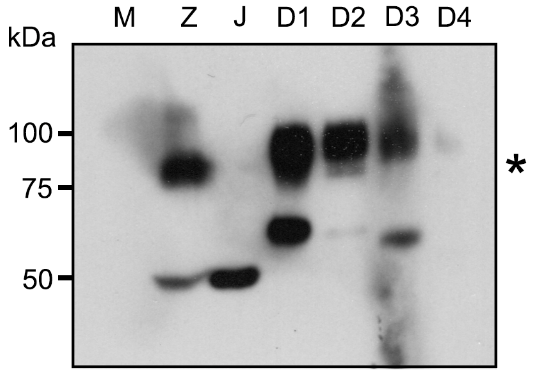

S54 S119

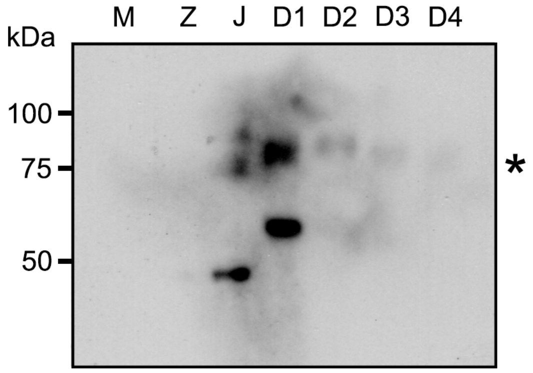

S55 S131

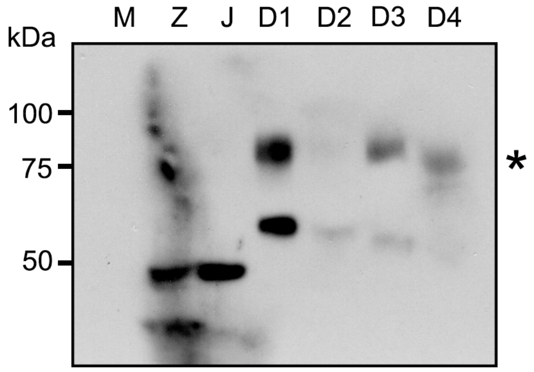

S56 S132

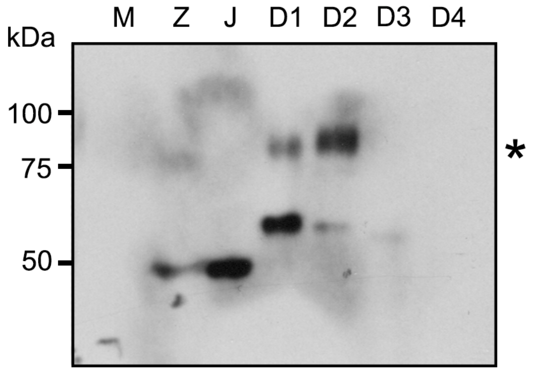

Figure S57-S60 Detection of anti-ZIKV NS1 dimer antibodies in Flavivirus $PRNT_{90} < 20$ human serum (negative control).

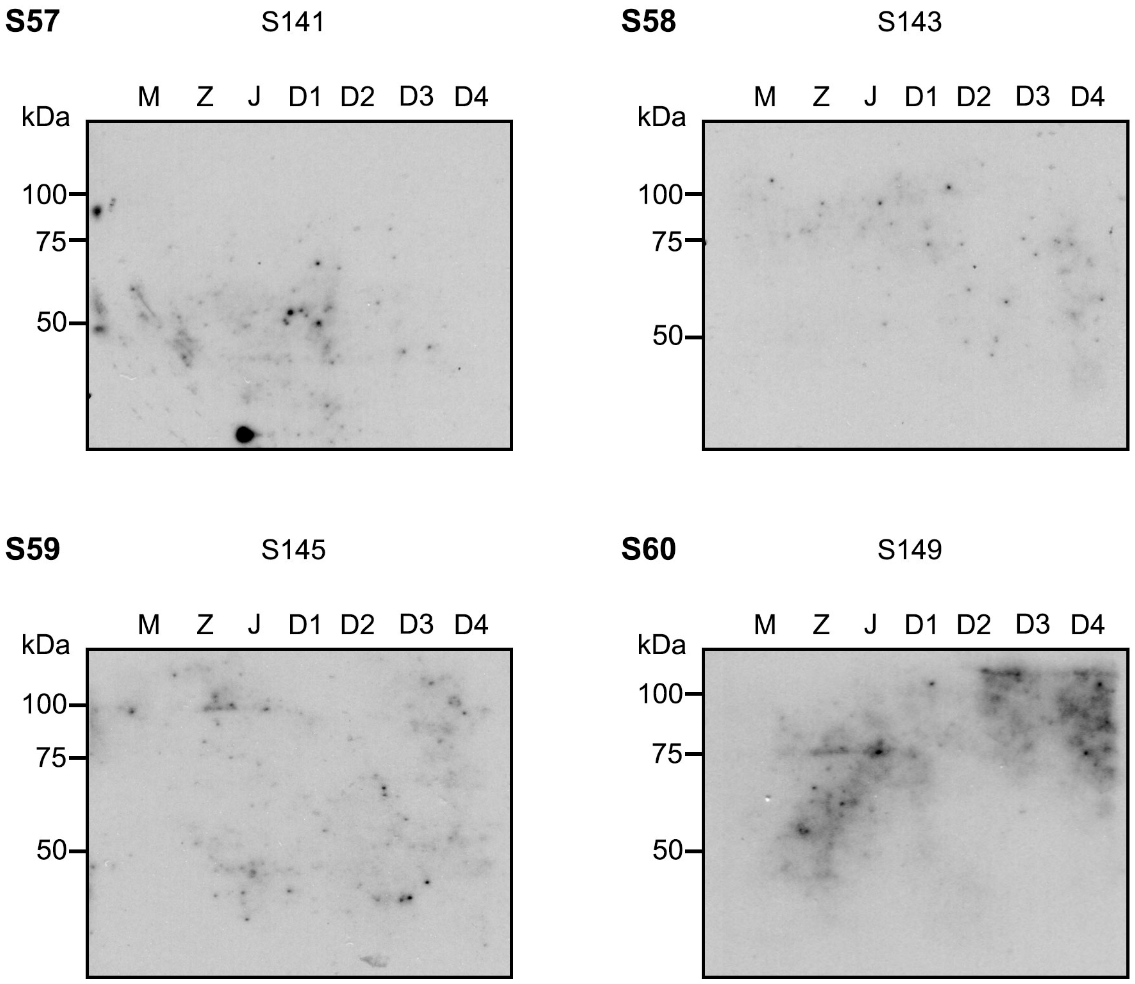

Supplemental Table 1 Full screen of neutralizing antibody titer for ZIKV, JEV and DENV (1-4) by plaque reduction neutralization test for 4 negative controls. PRNT90 ≥20 was defined as positive.

Sample	Gender	Age	ZIKV	JEV	DENV 1	DENV 2	DENV 3	DENV 4
S141	F	25	< 20	< 20	< 20	< 20	< 20	< 20
S143	F	25	< 20	< 20	< 20	< 20	< 20	< 20
S145	F	21	< 20	< 20	< 20	< 20	< 20	< 20
S149	F	21	< 20	< 20	< 20	< 20	< 20	< 20
