# Supplementary material for: High correlation between Zika virus NS1 antibodies and neutralizing antibodies in selected serum samples from normal healthy Thais
Source: Sci Rep. 2019 Sep 18;9:13498. doi: 10.1038/s41598-019-49569-0 (PMC6751300; doi:10.1038/s41598-019-49569-0)

Figure 1A

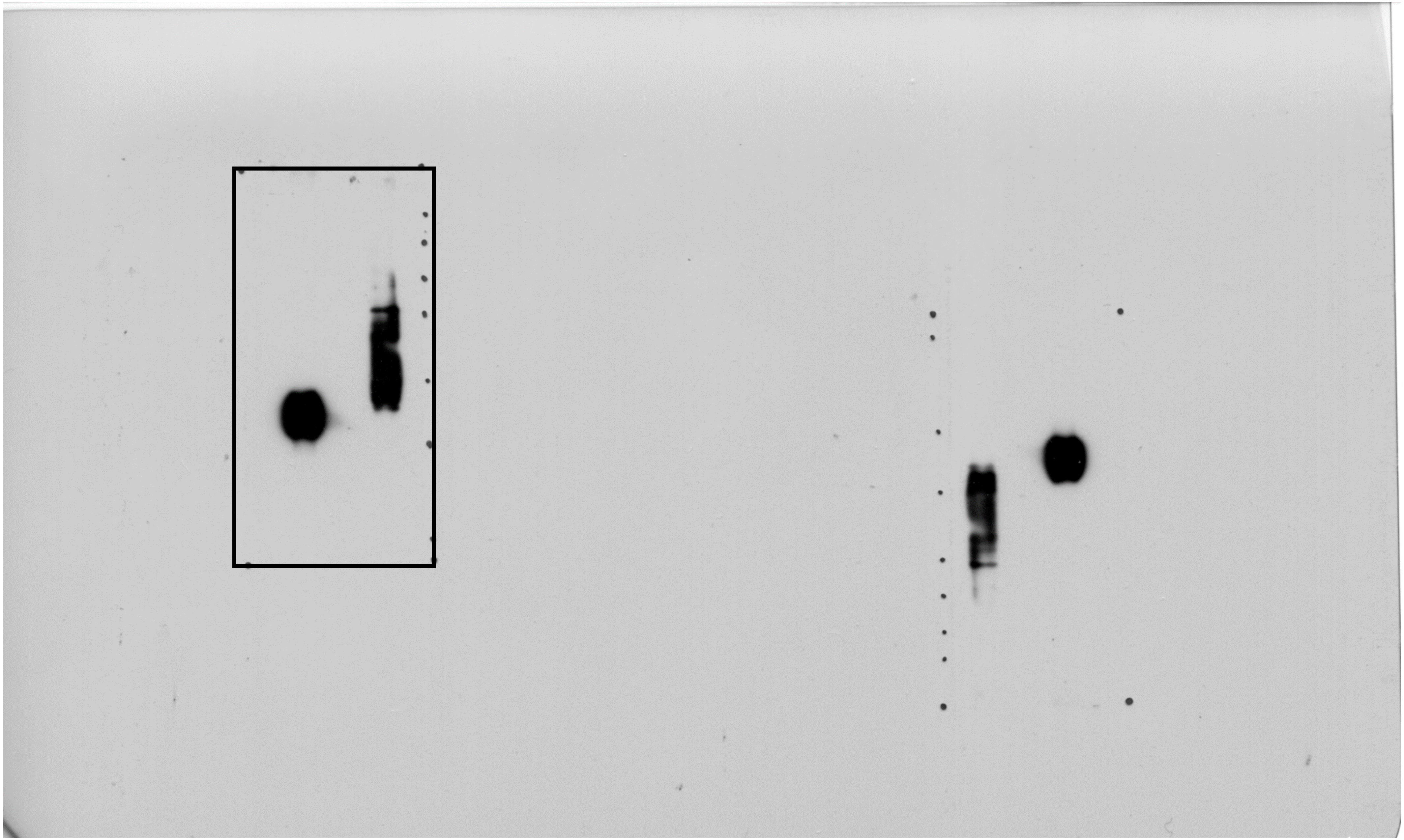

Figure 1B

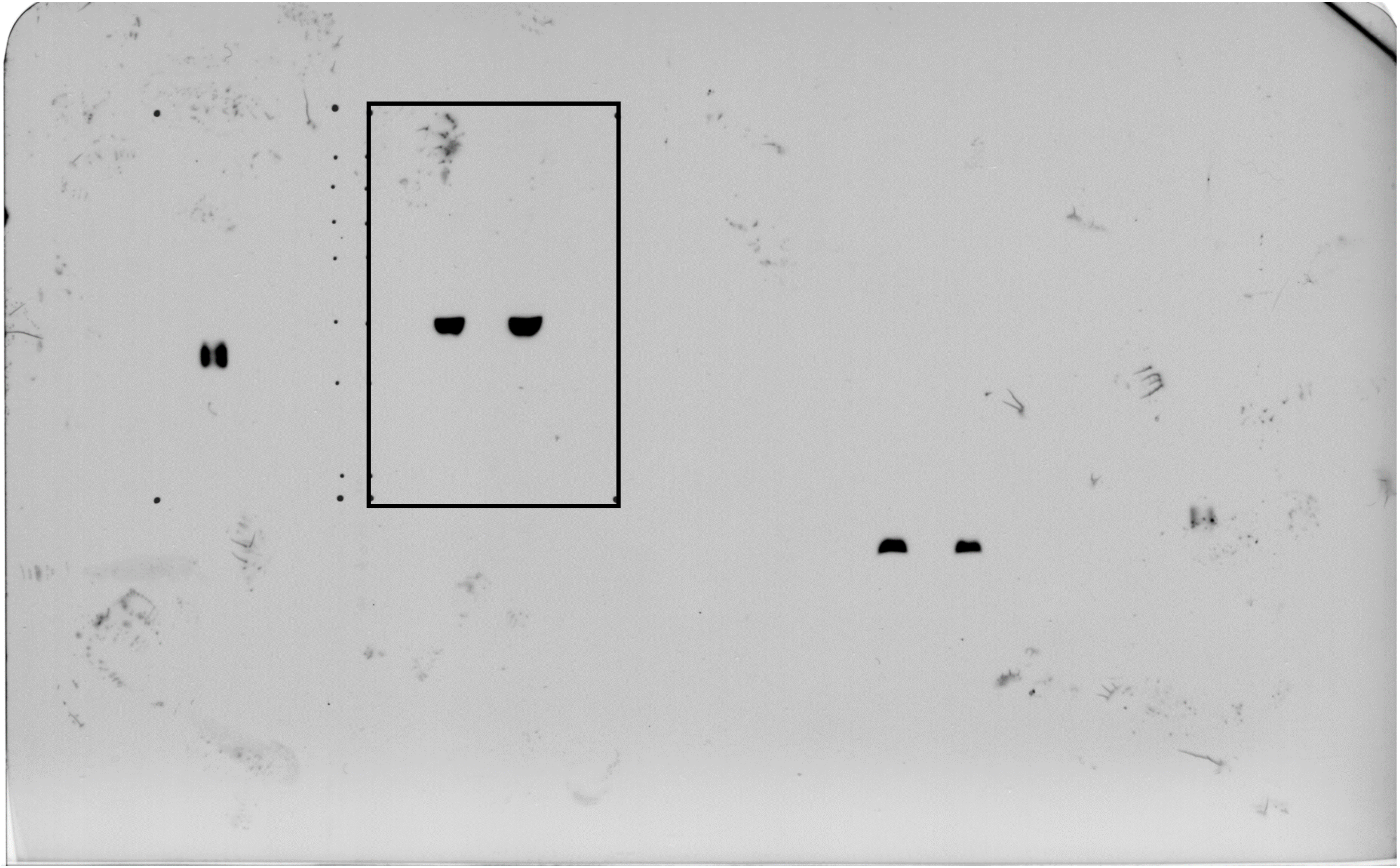

Figure 1C

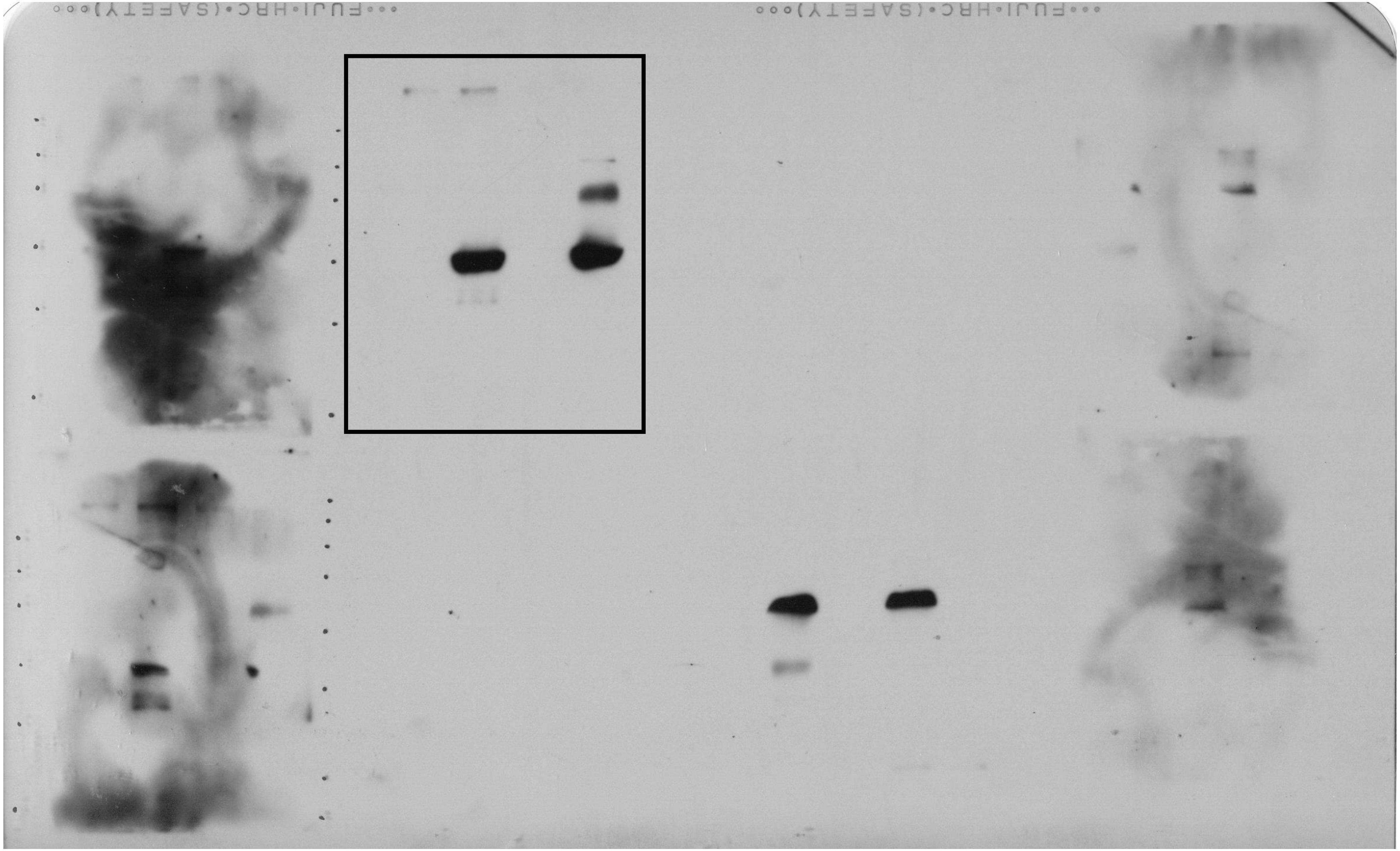

Figure 2A

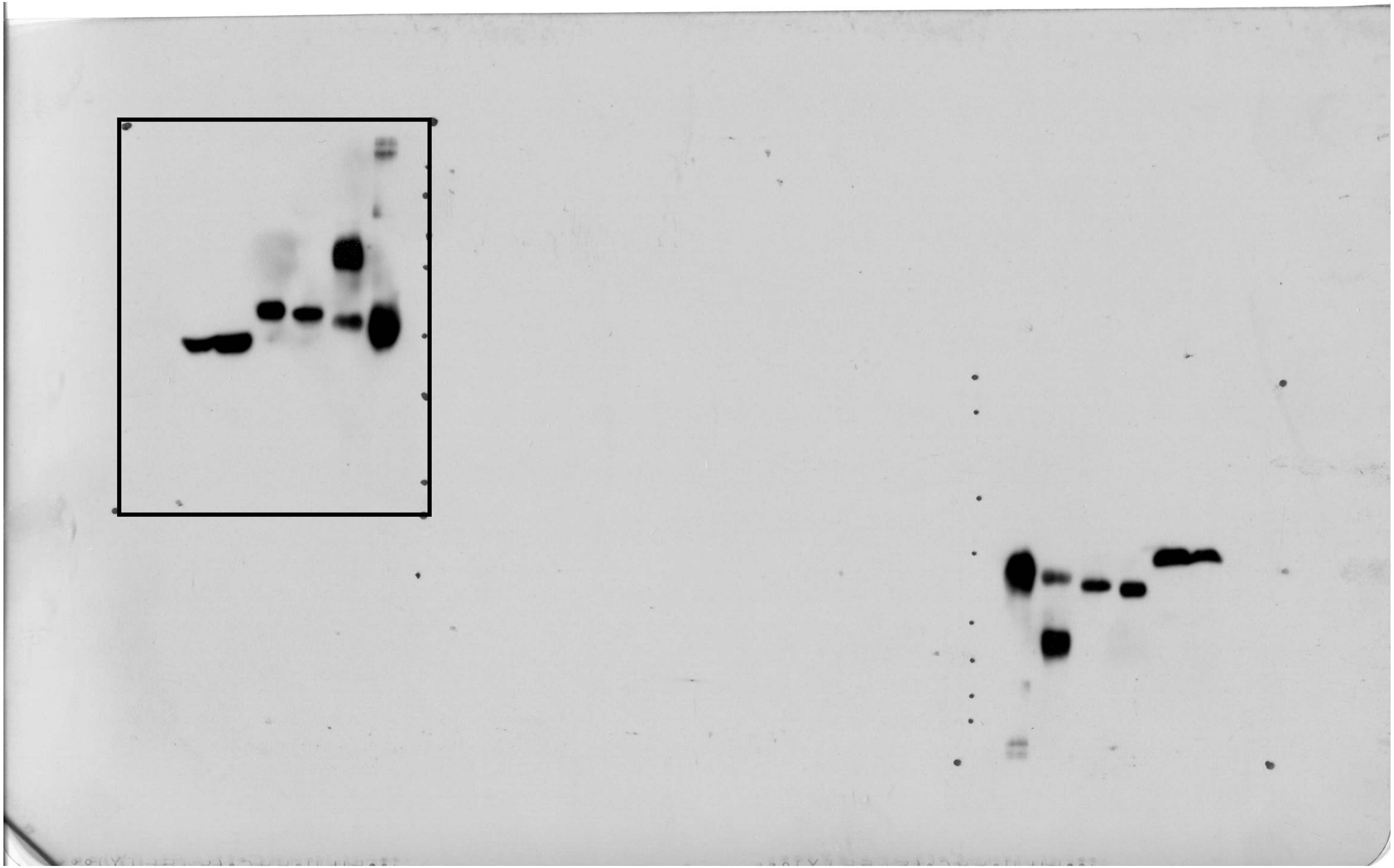

Figure 2B

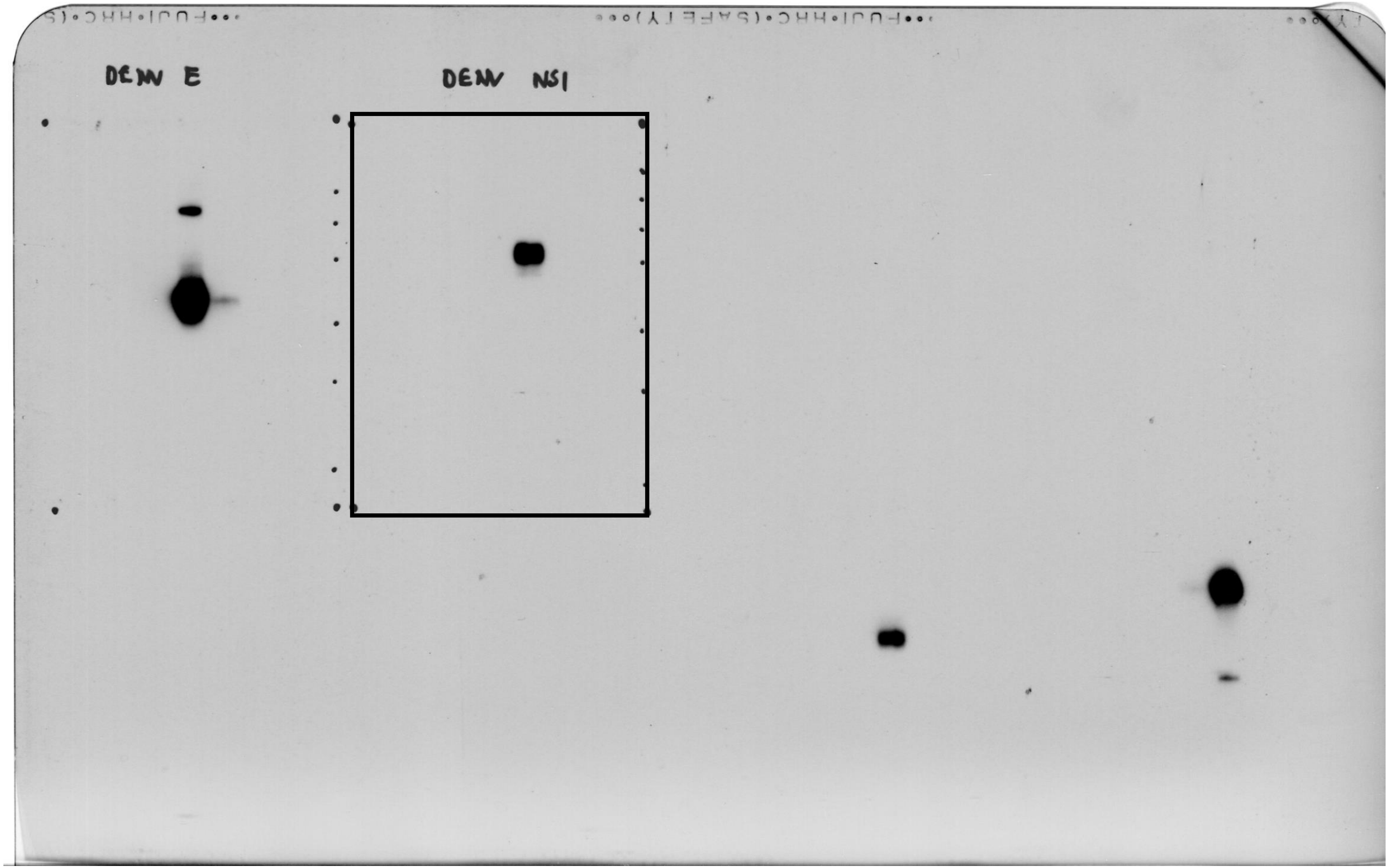

Figure 2C

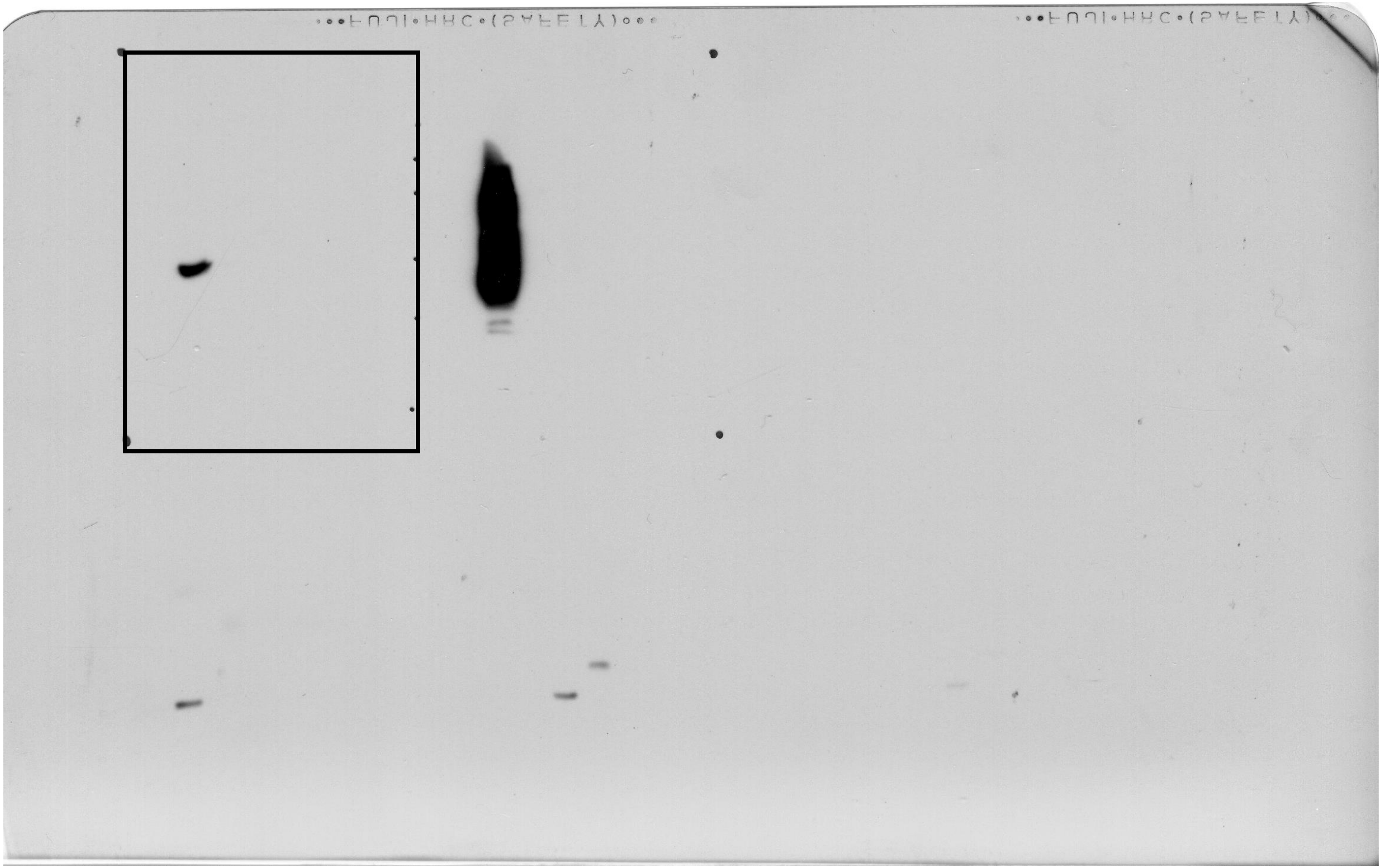

Figure 2D

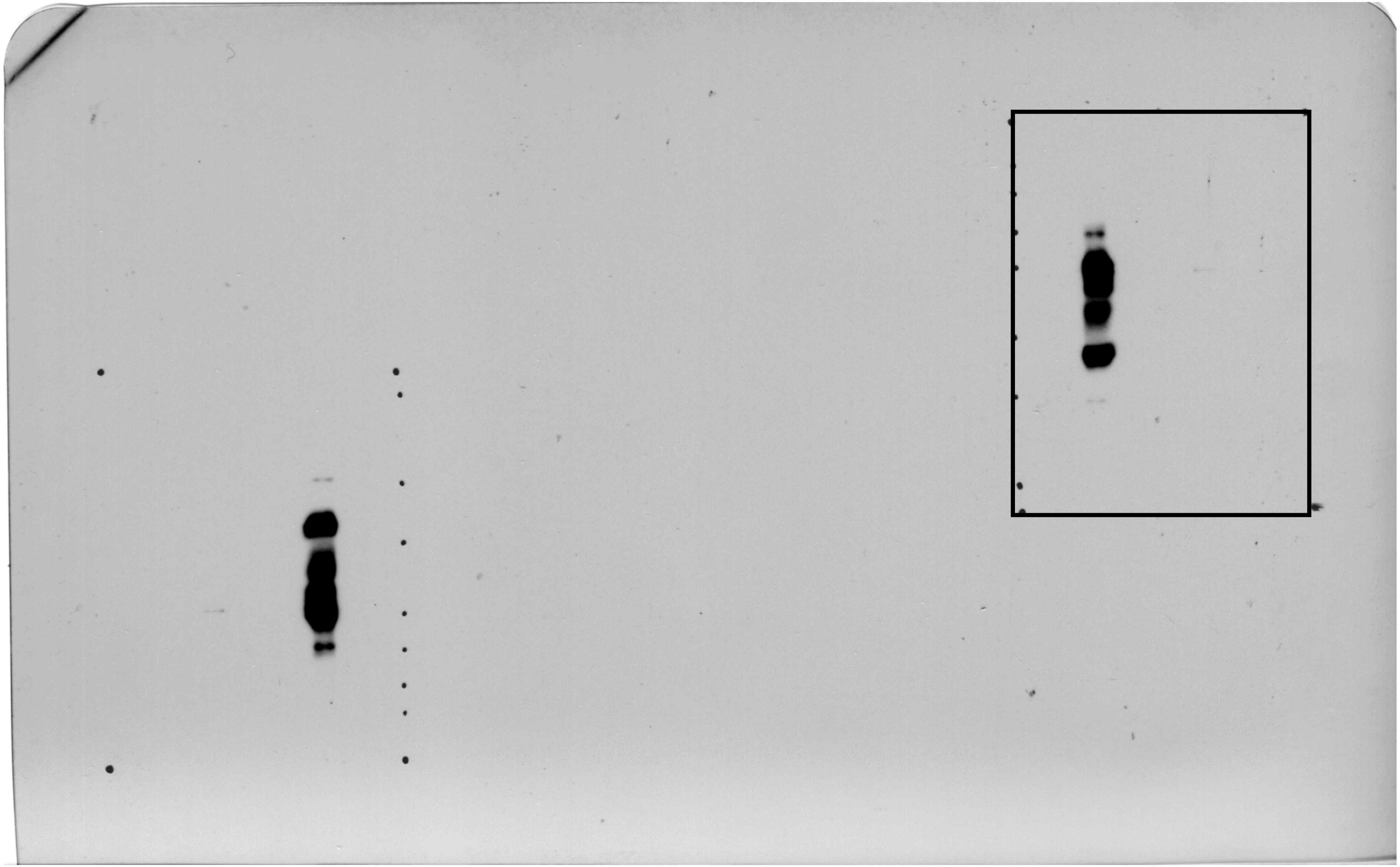

Figure 3A

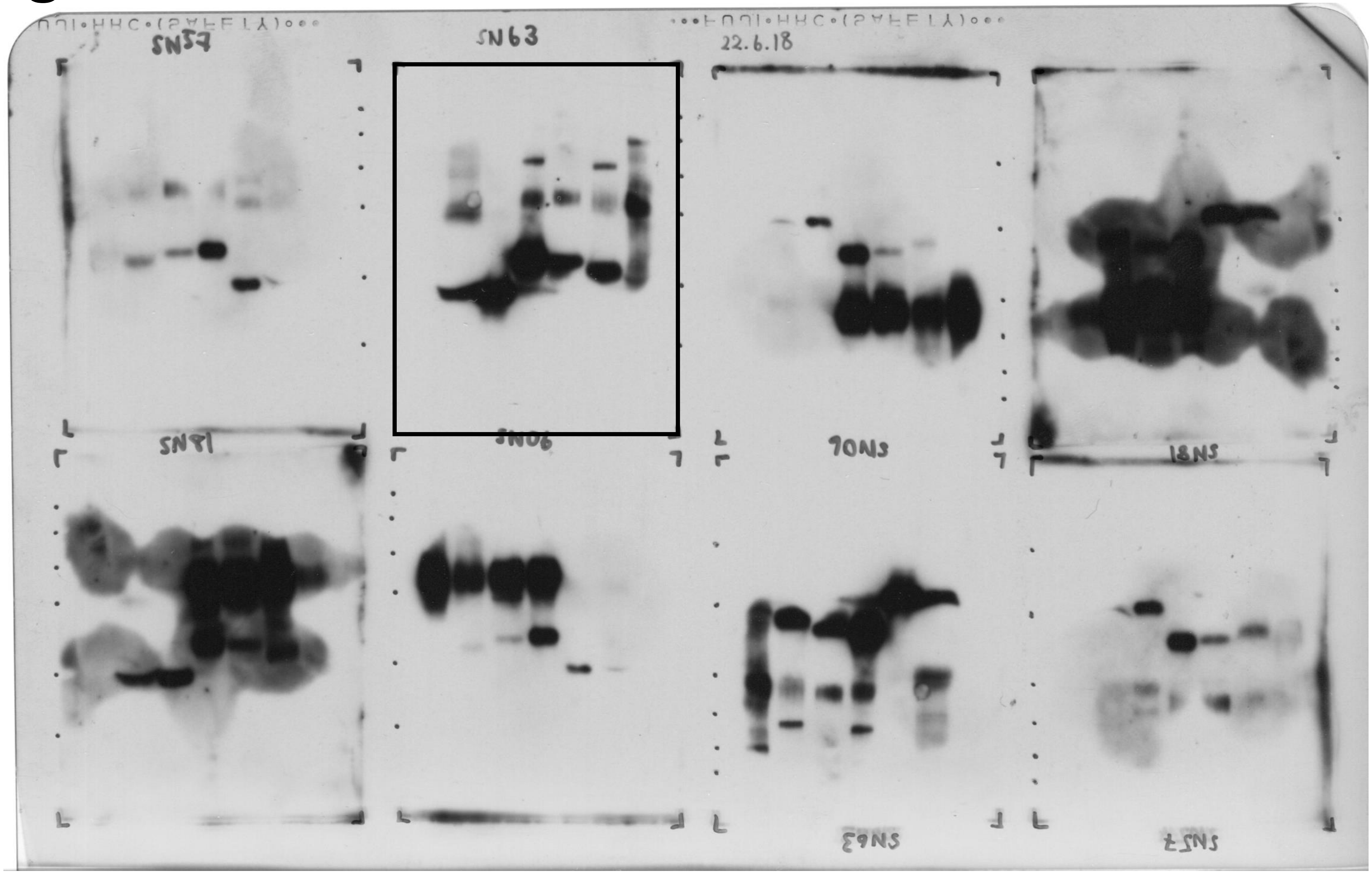

Figure 3B

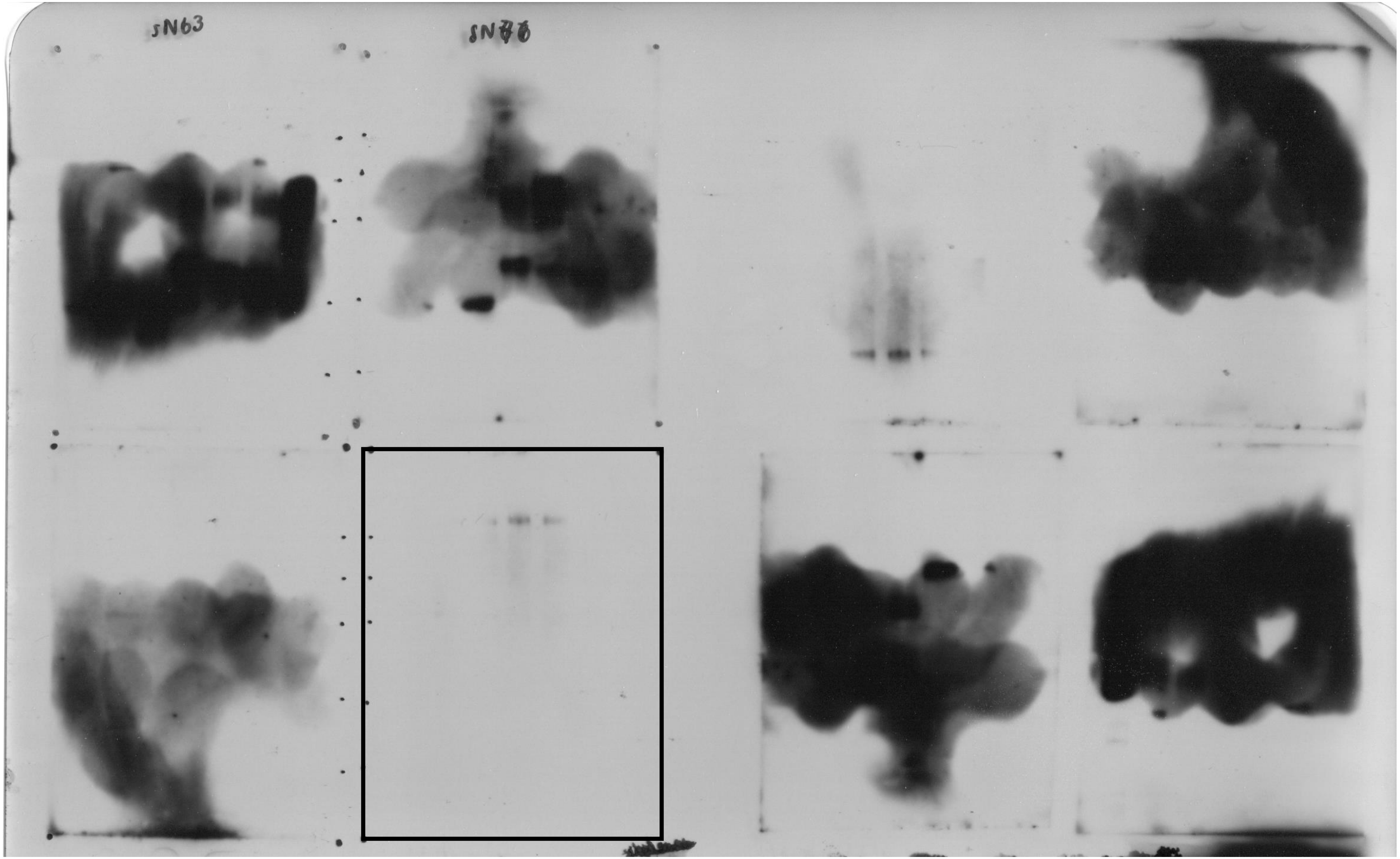

Figure 4A

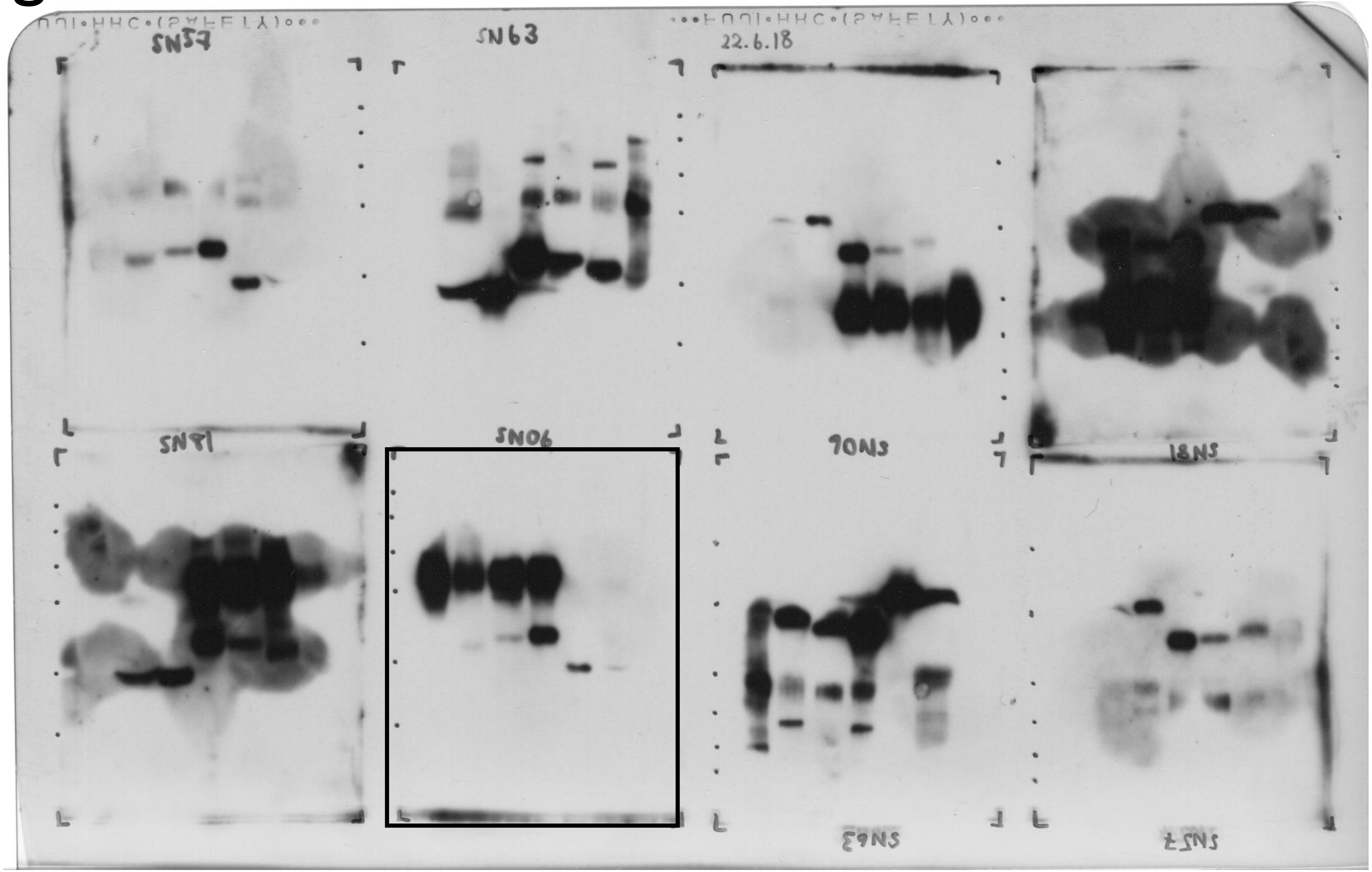

Figure 4B

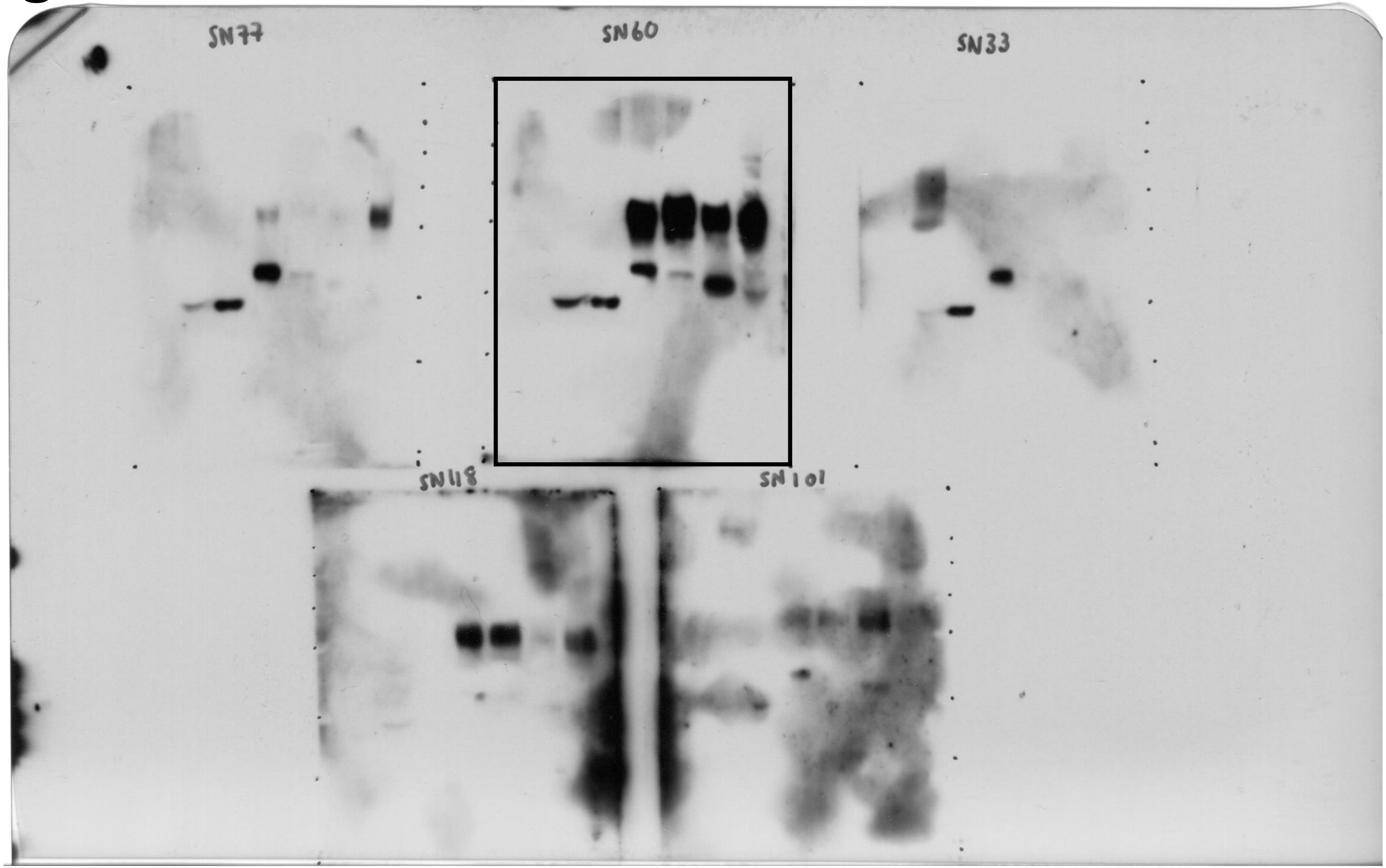

Figure 5A

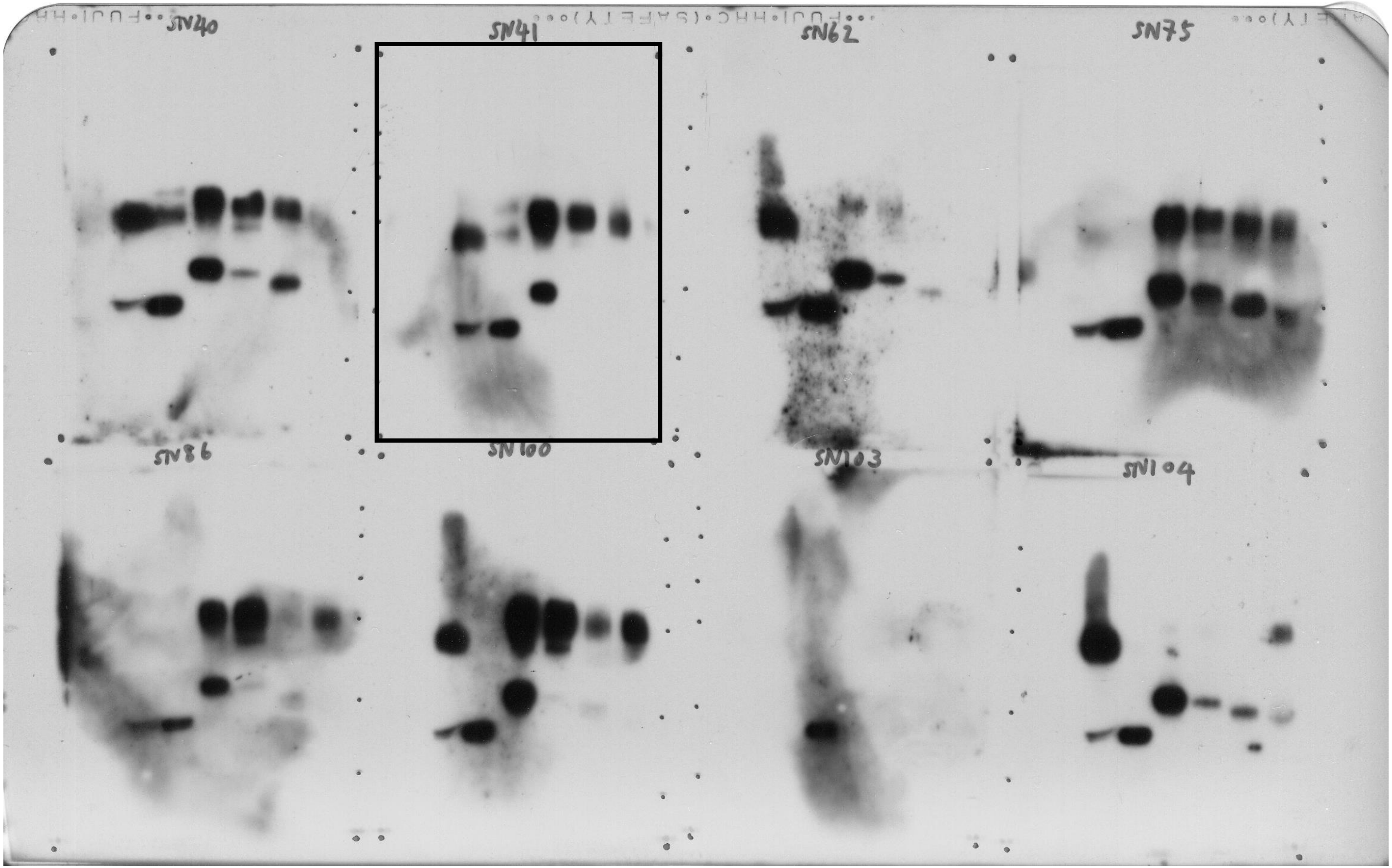

Figure 5B

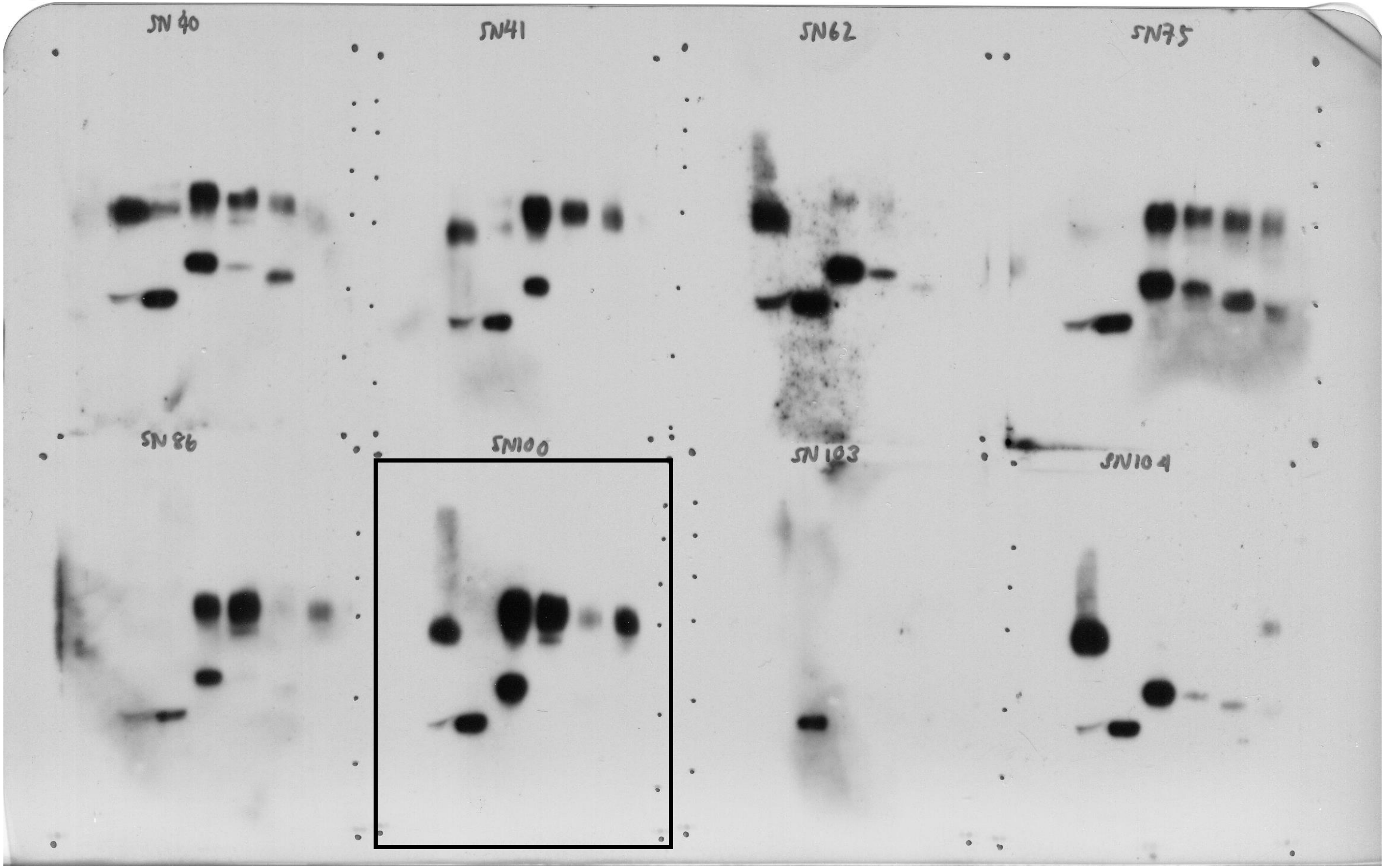

Figure S1

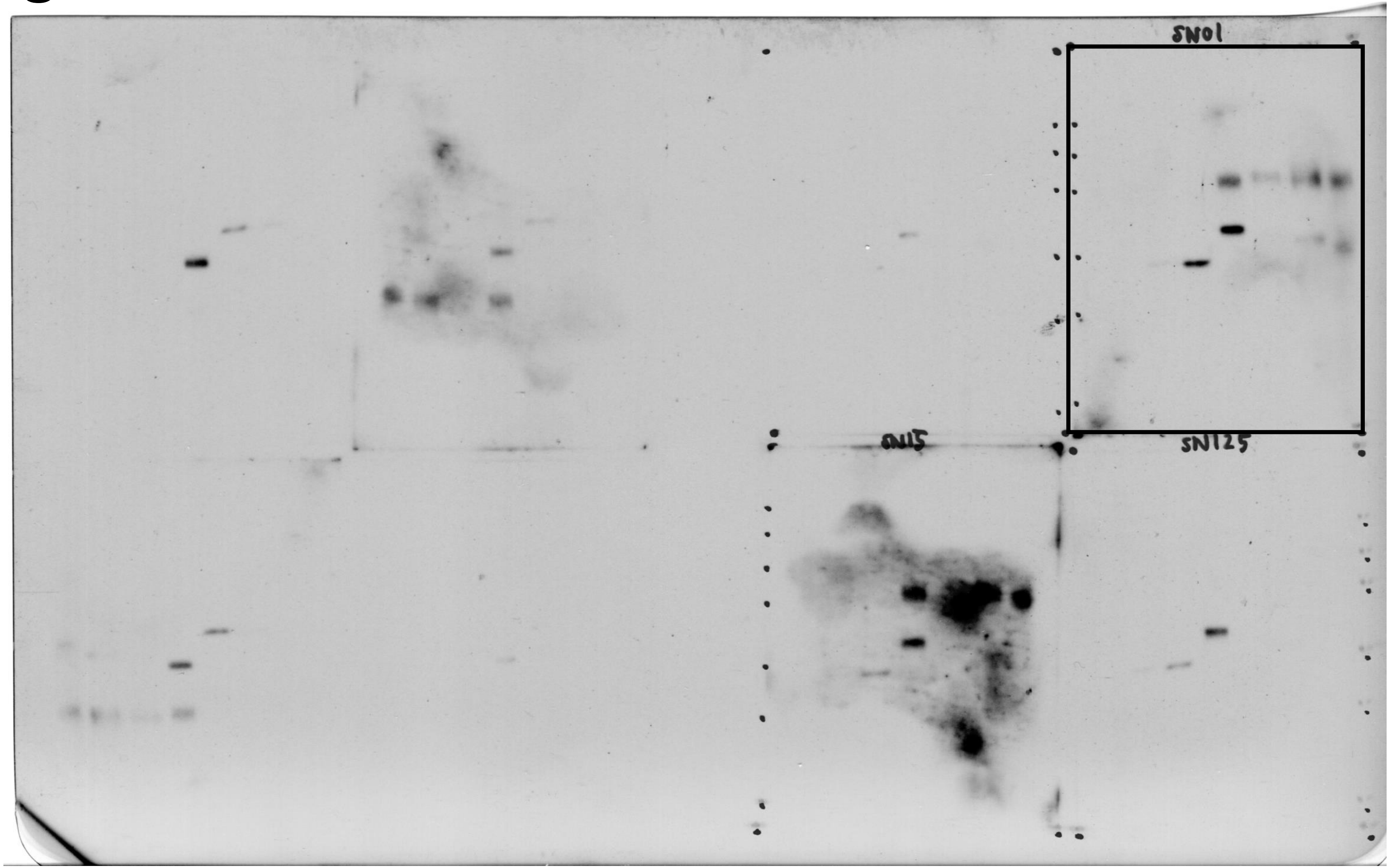

Figure S2

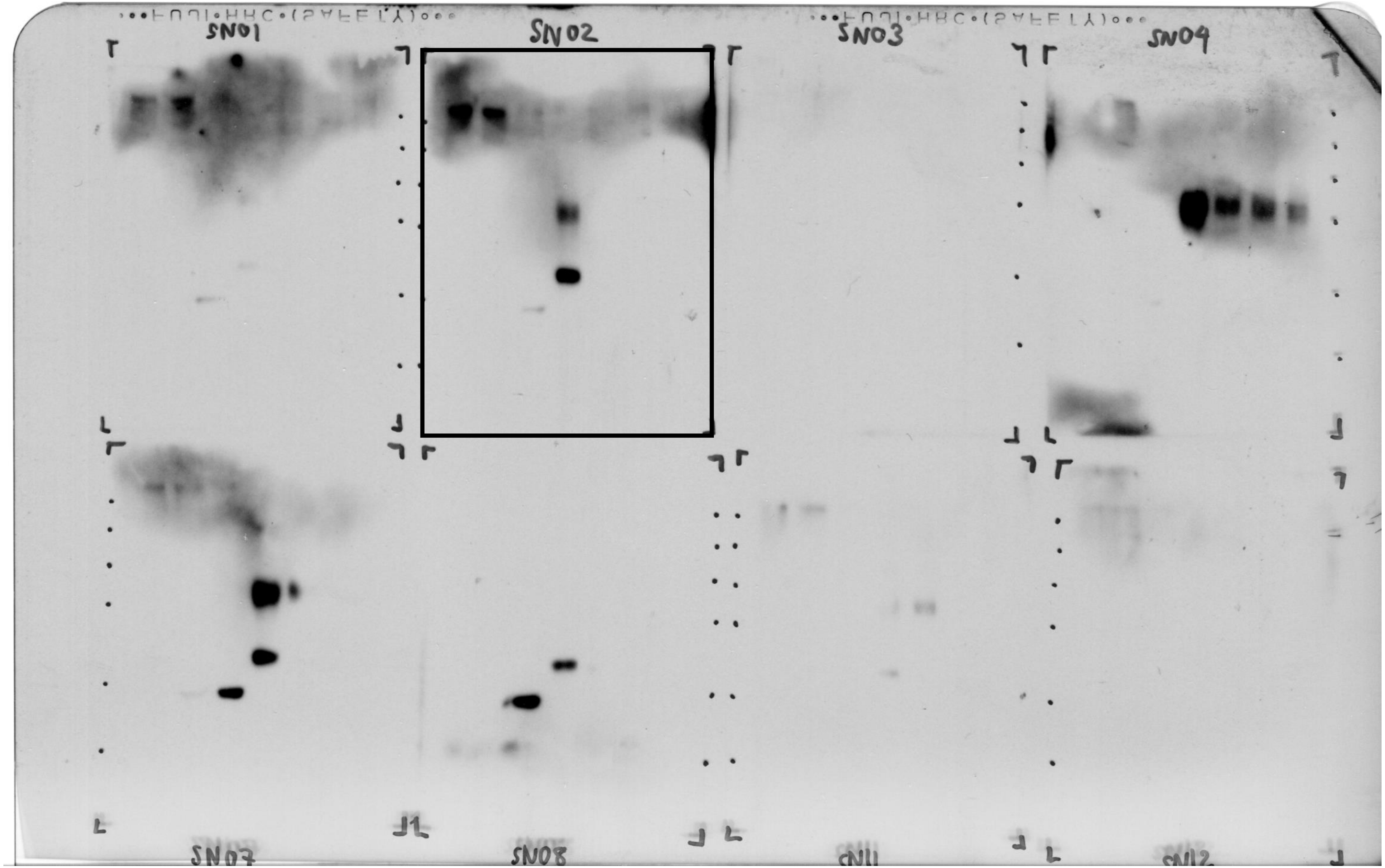

Figure S3

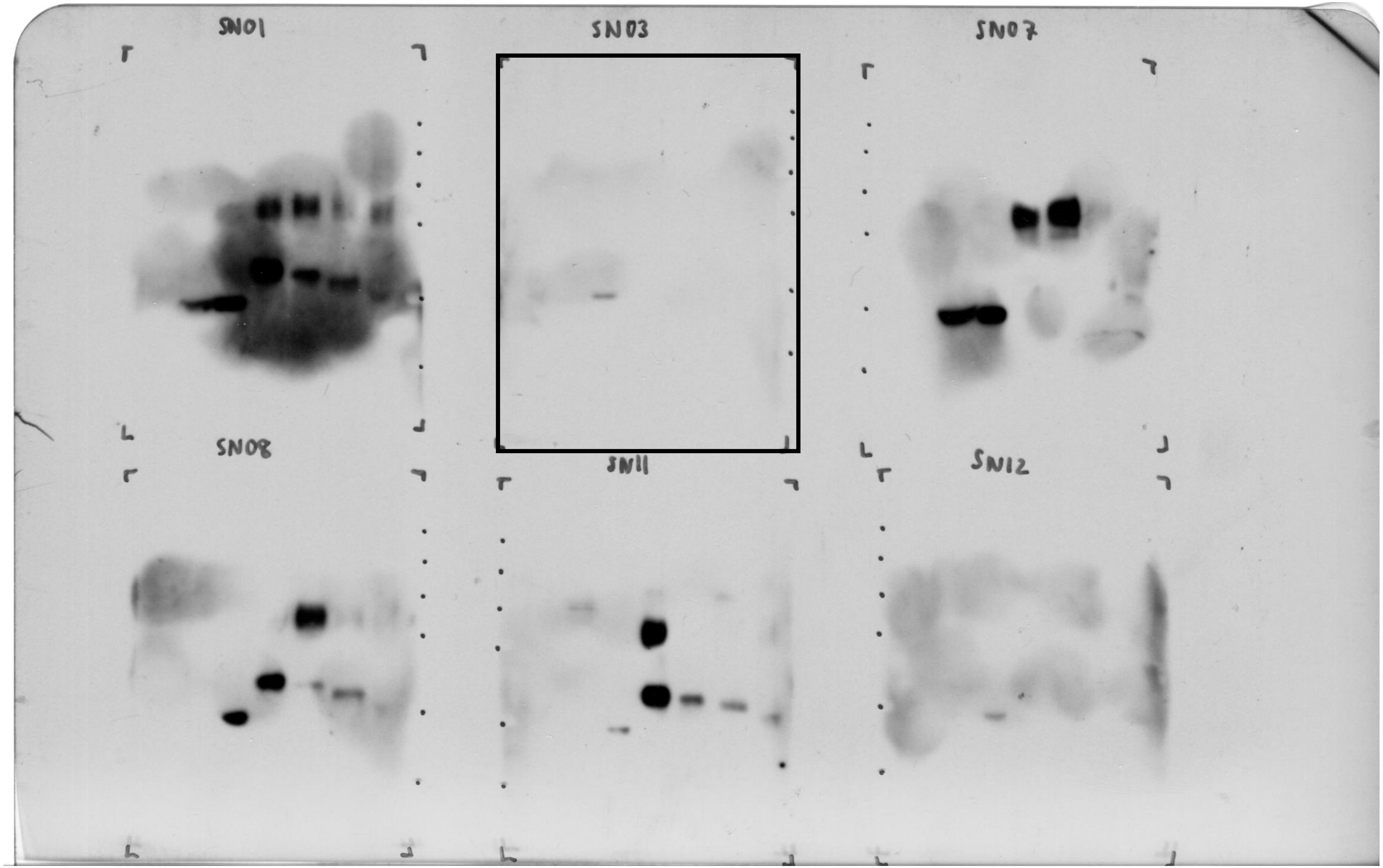

Figure S4

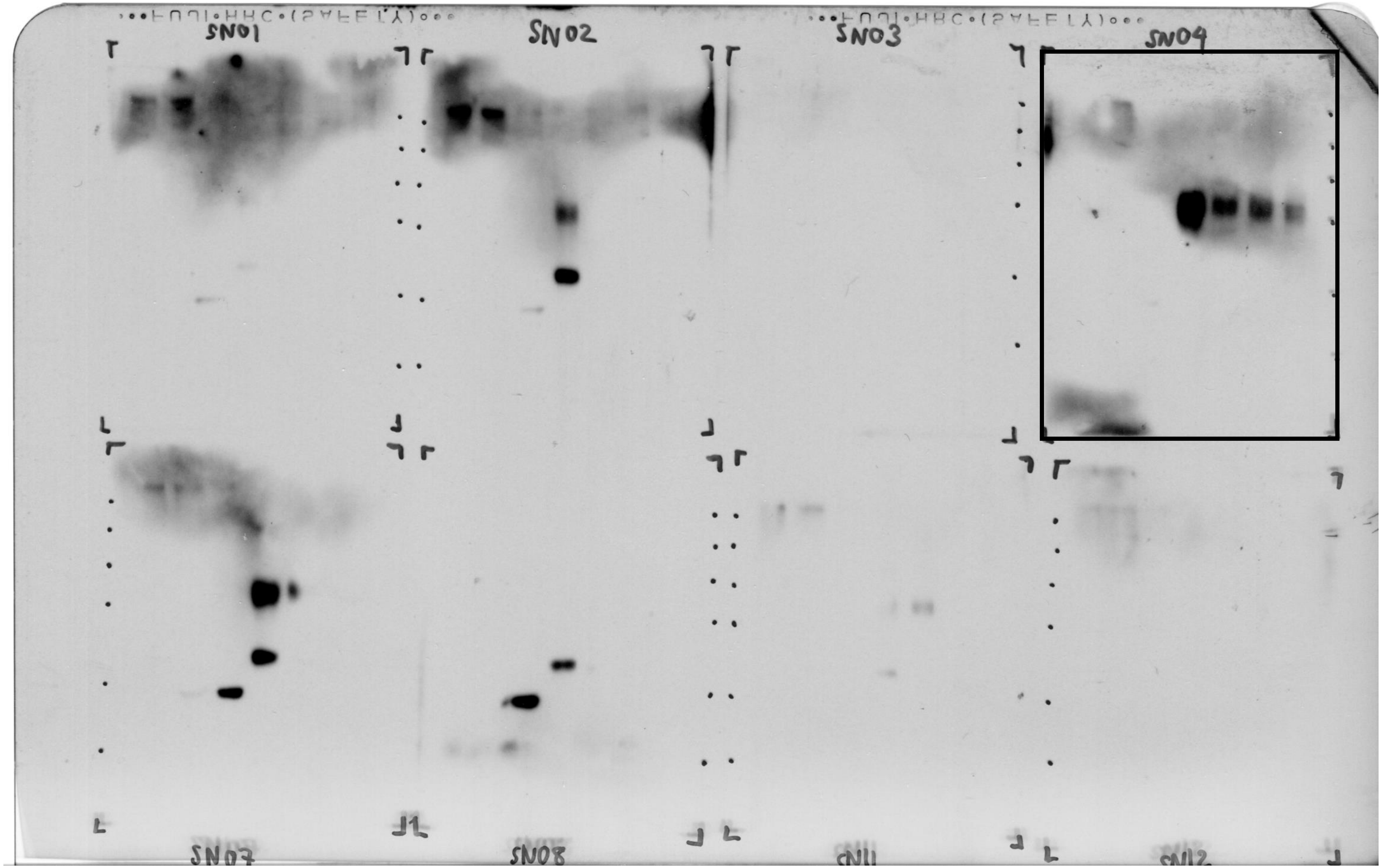

Figure S5

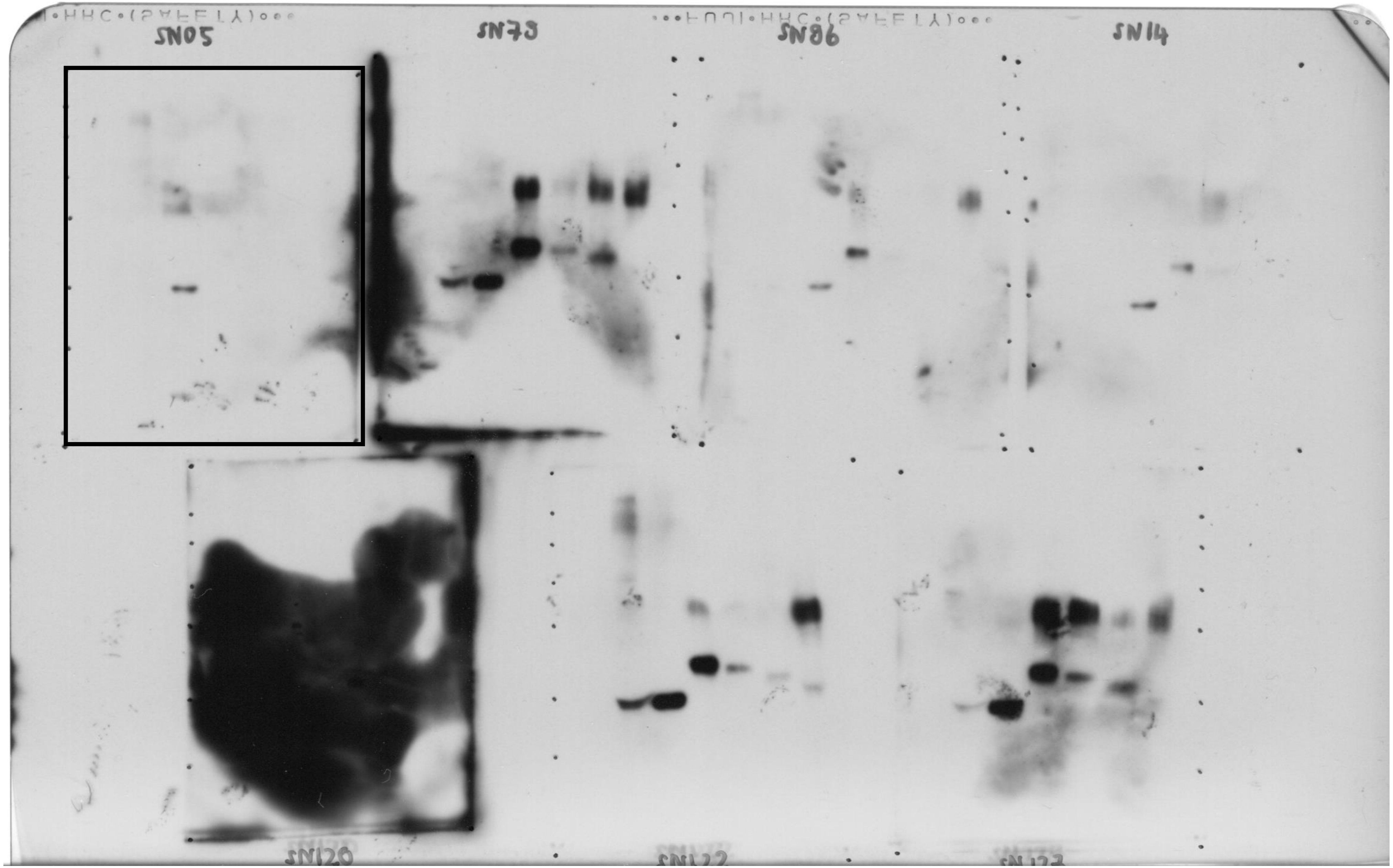

Figure S6

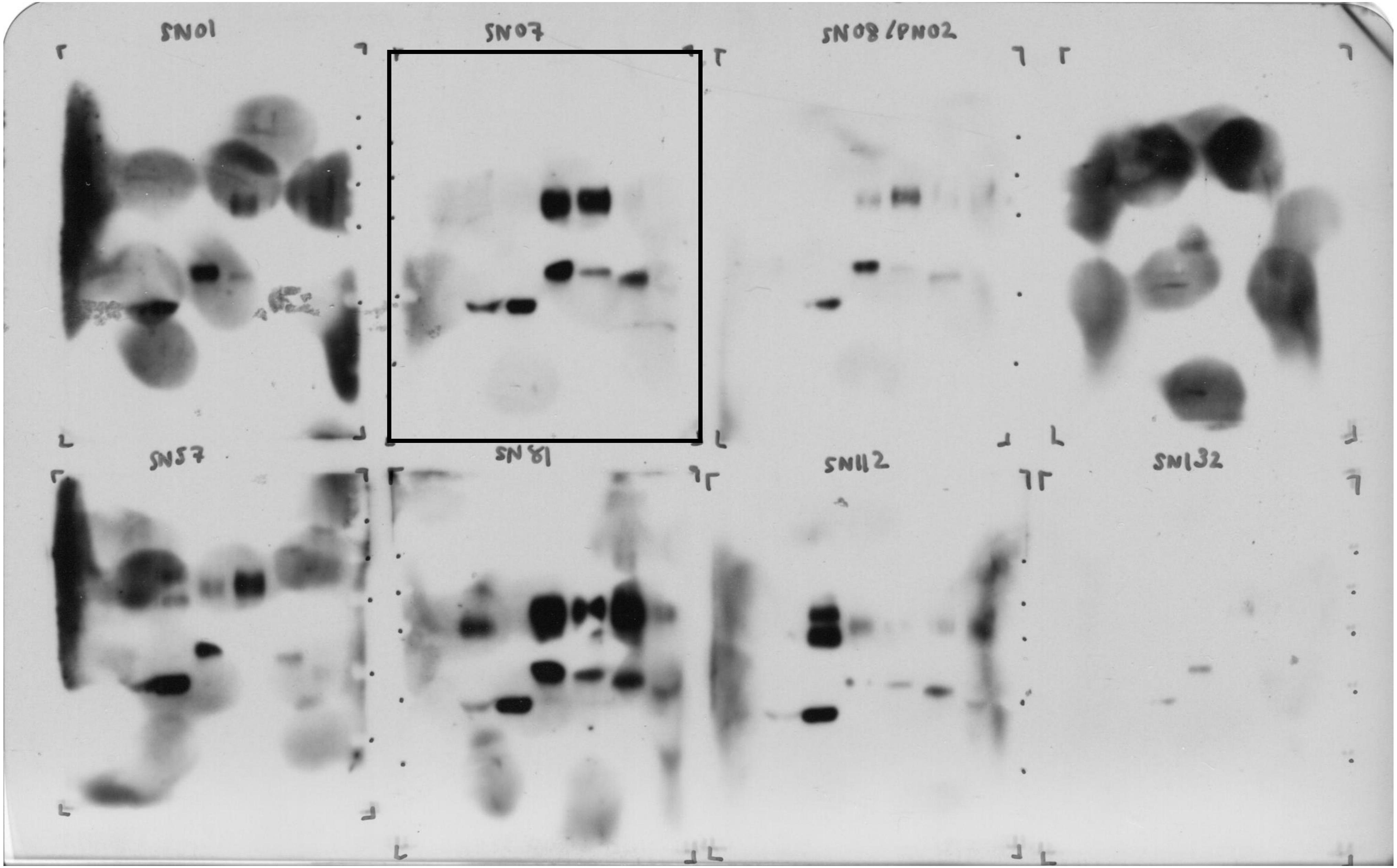

Figure S7

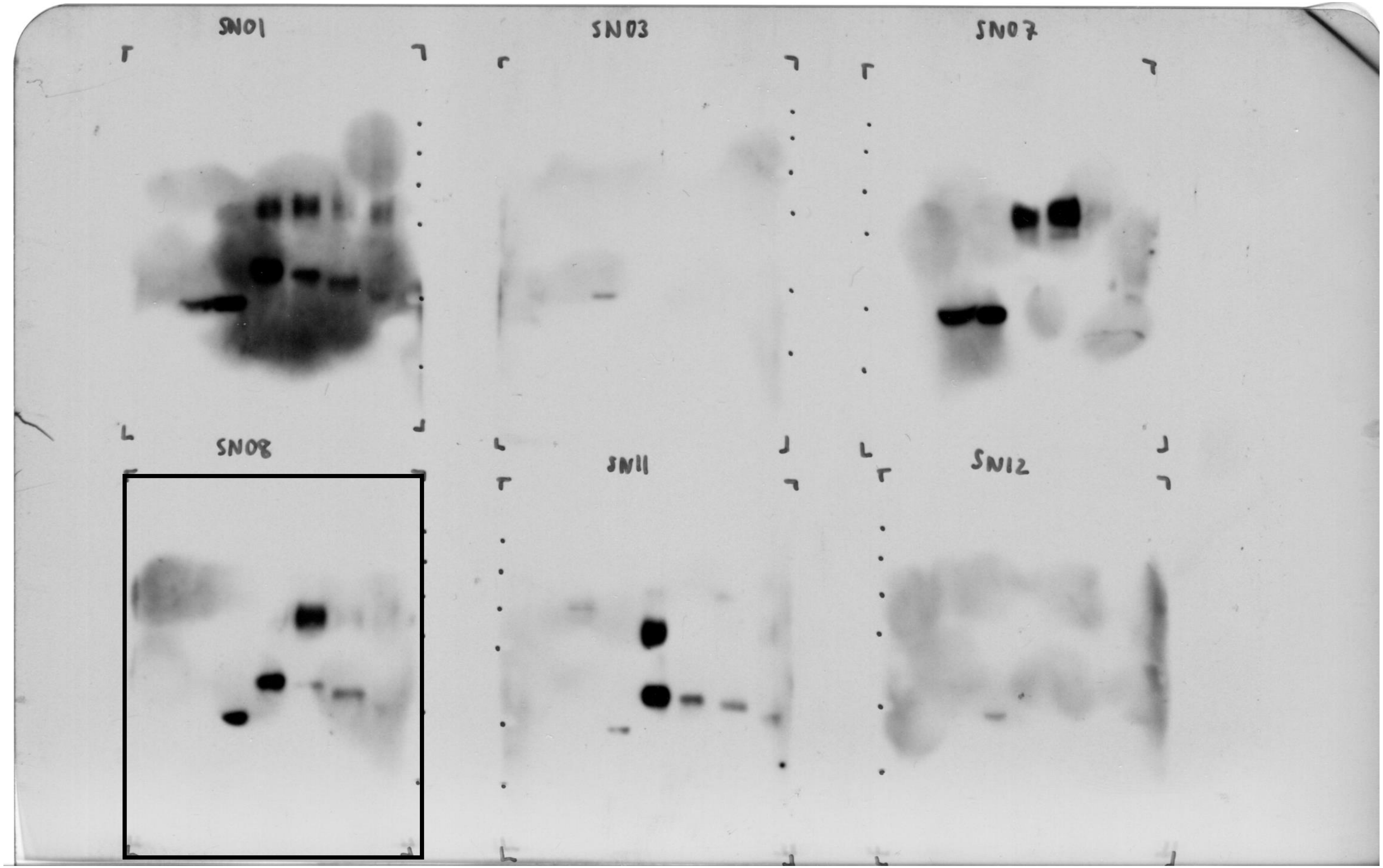

Figure S8

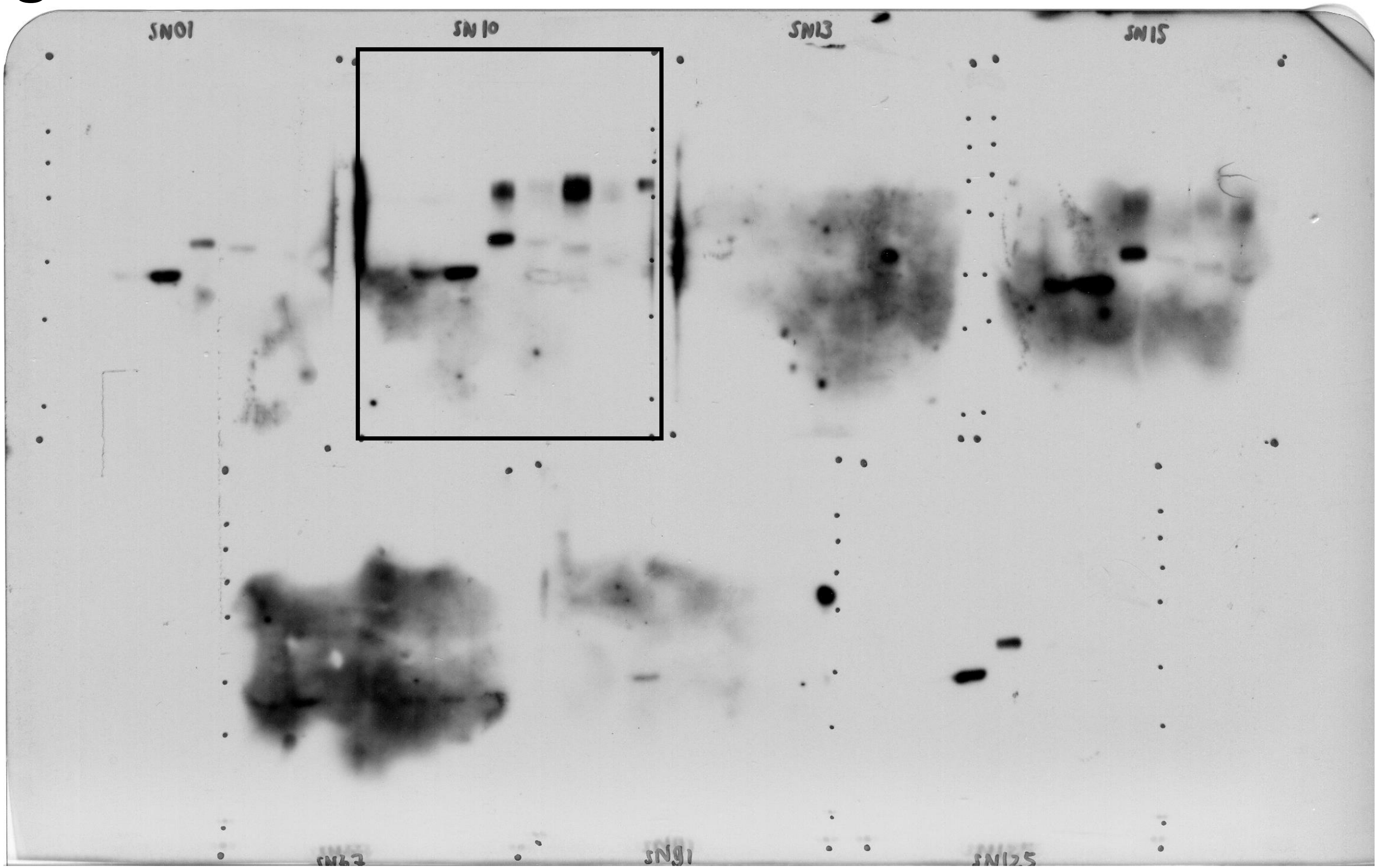

Figure S9

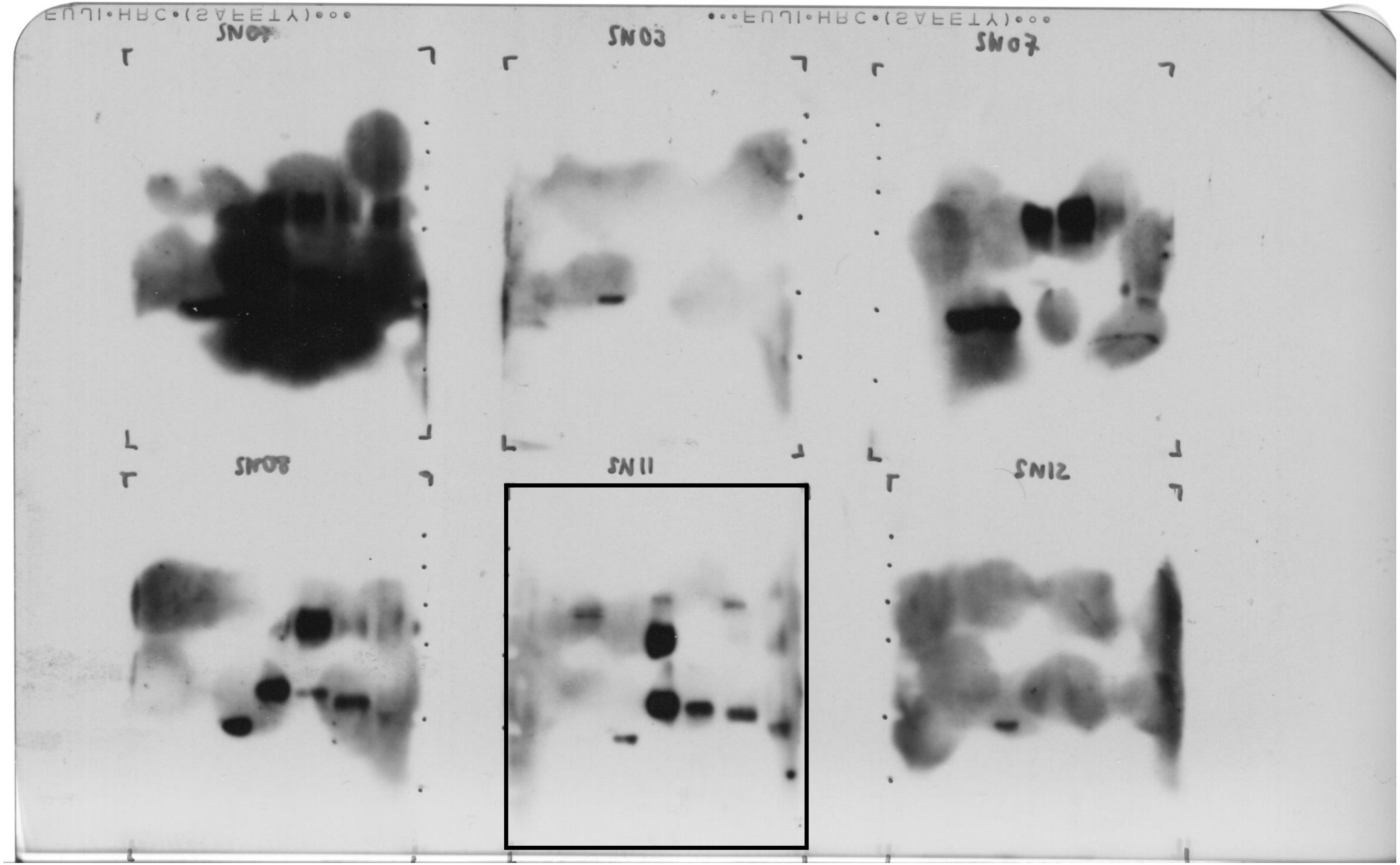

Figure S10

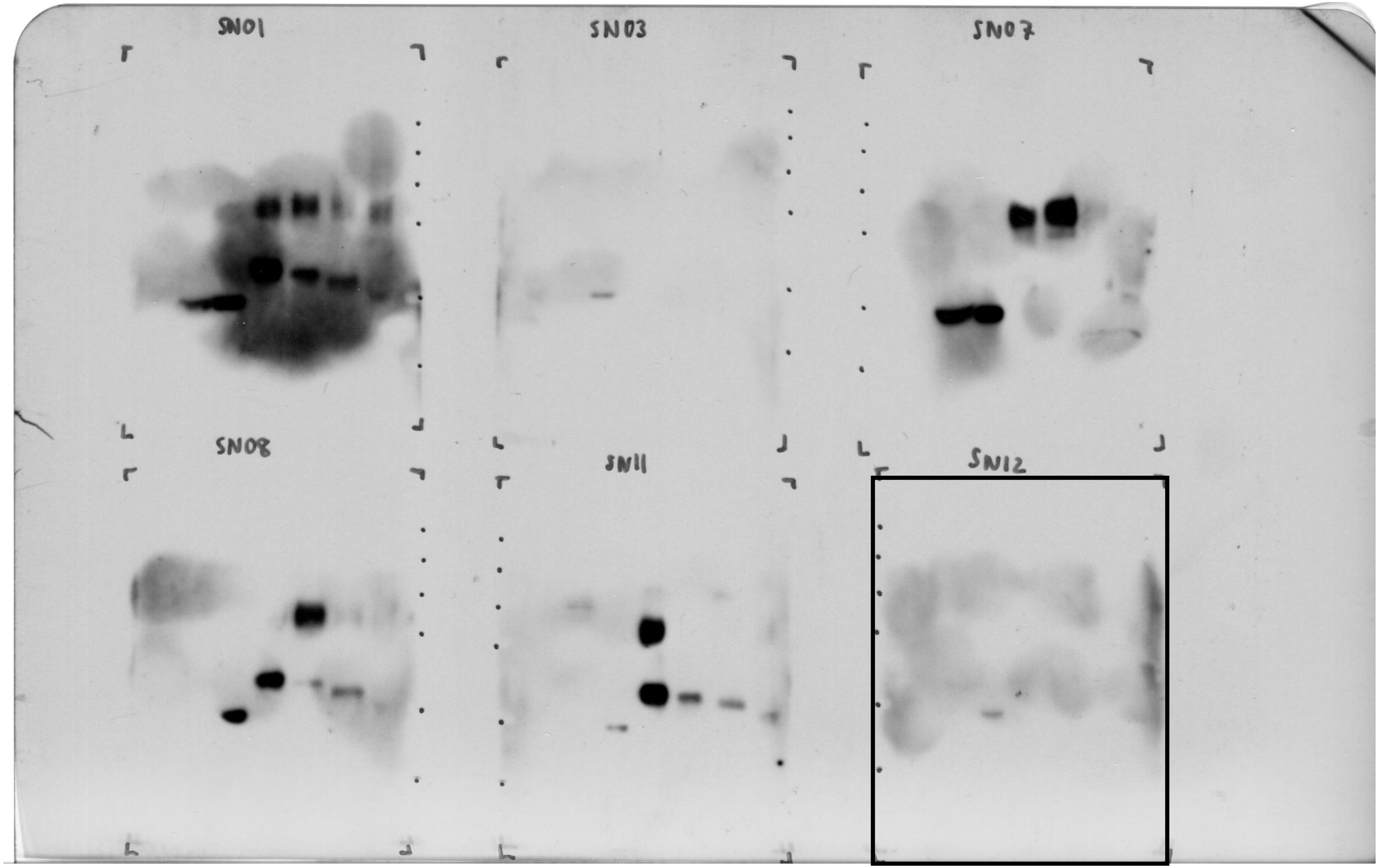

Figure S11

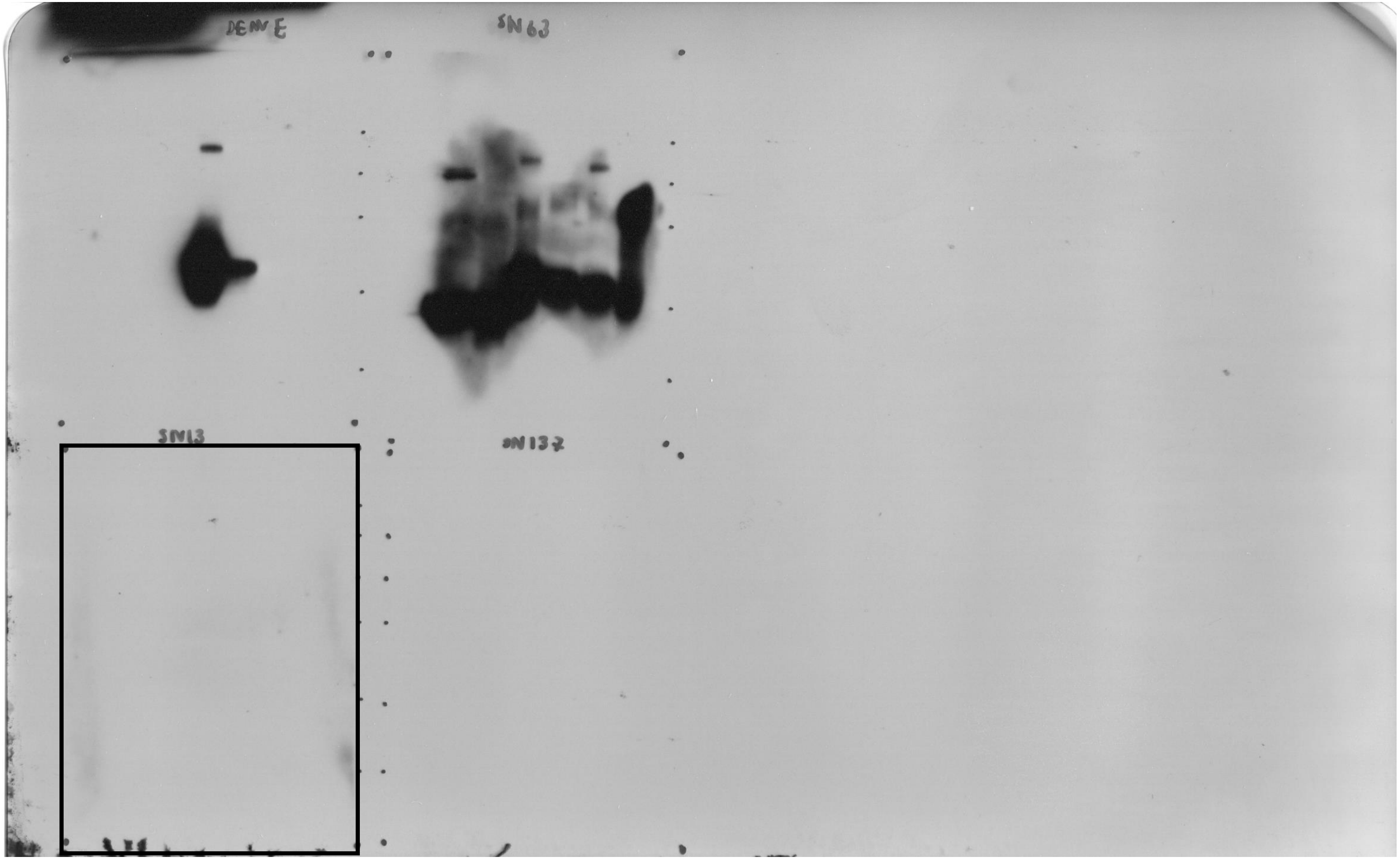

Figure S12

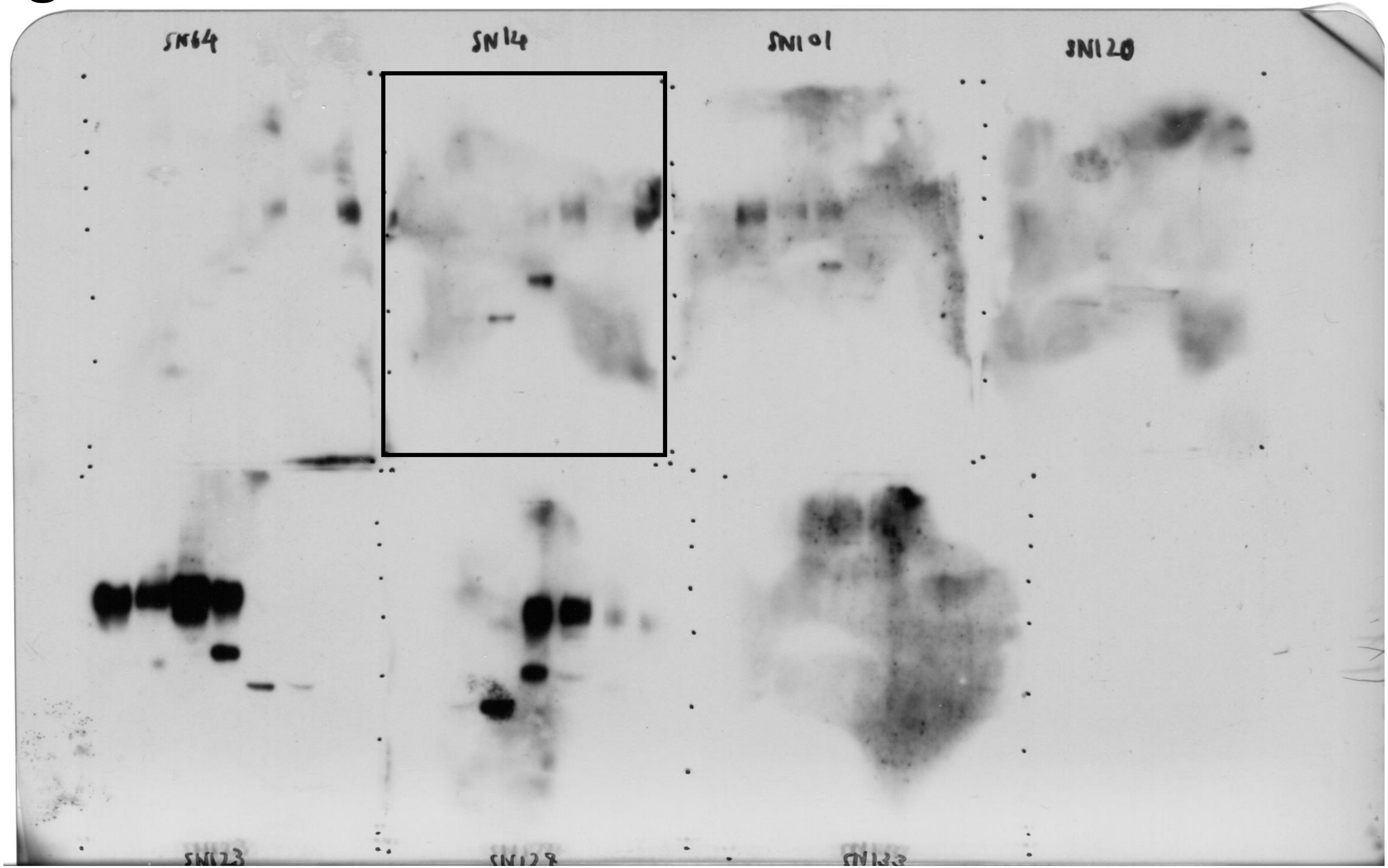

Figure S13

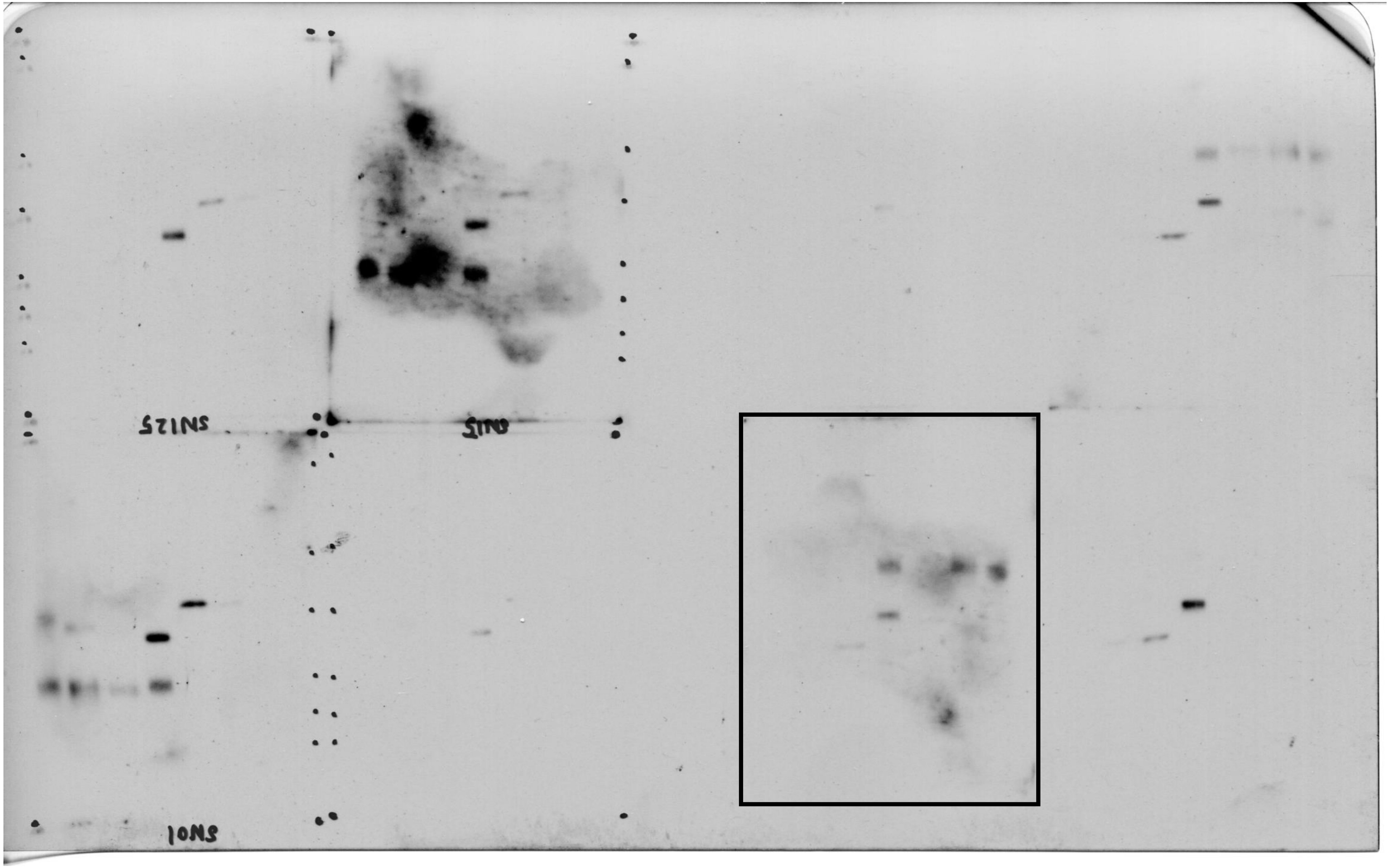

Figure S14

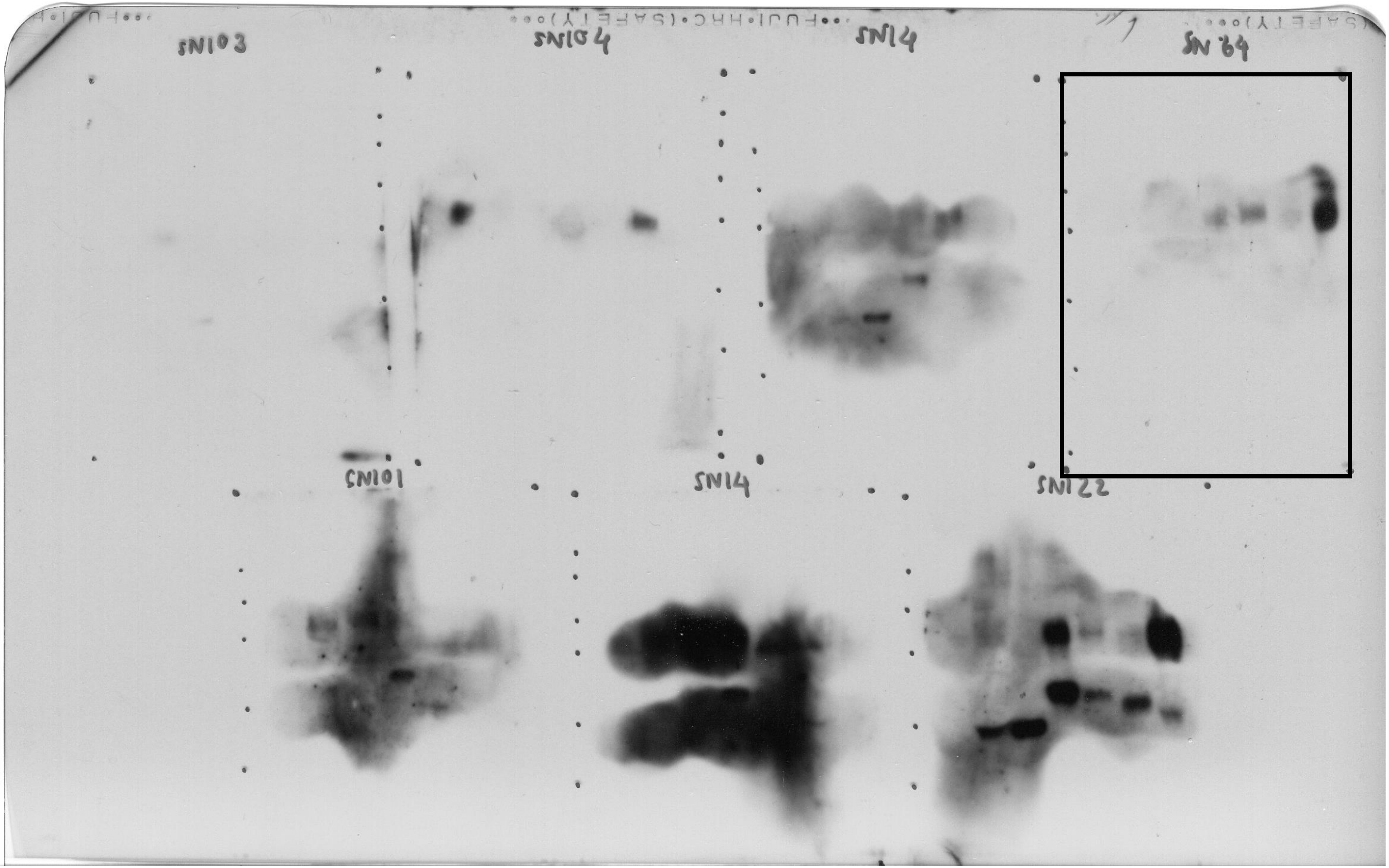

Figure S15

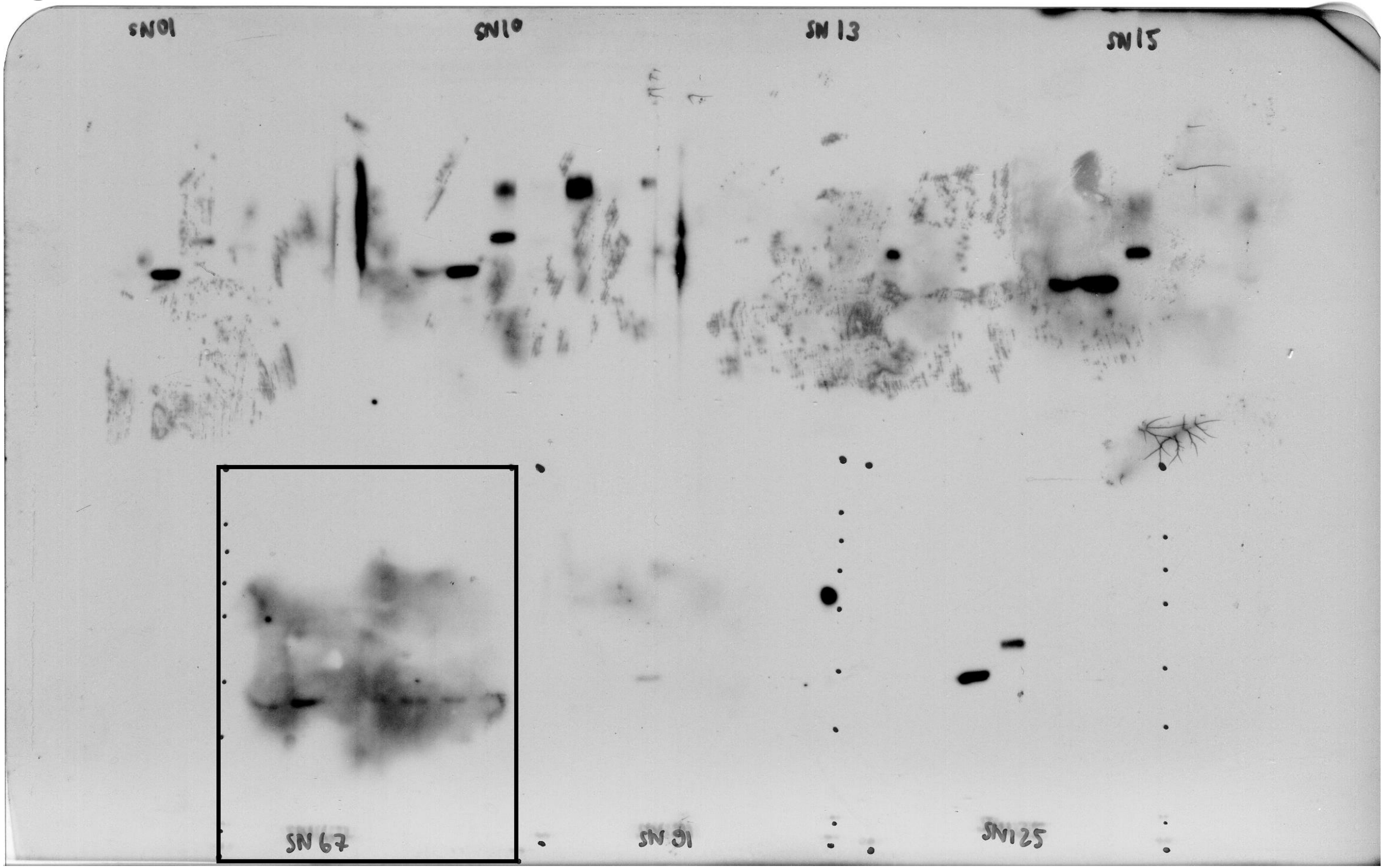

Figure S16

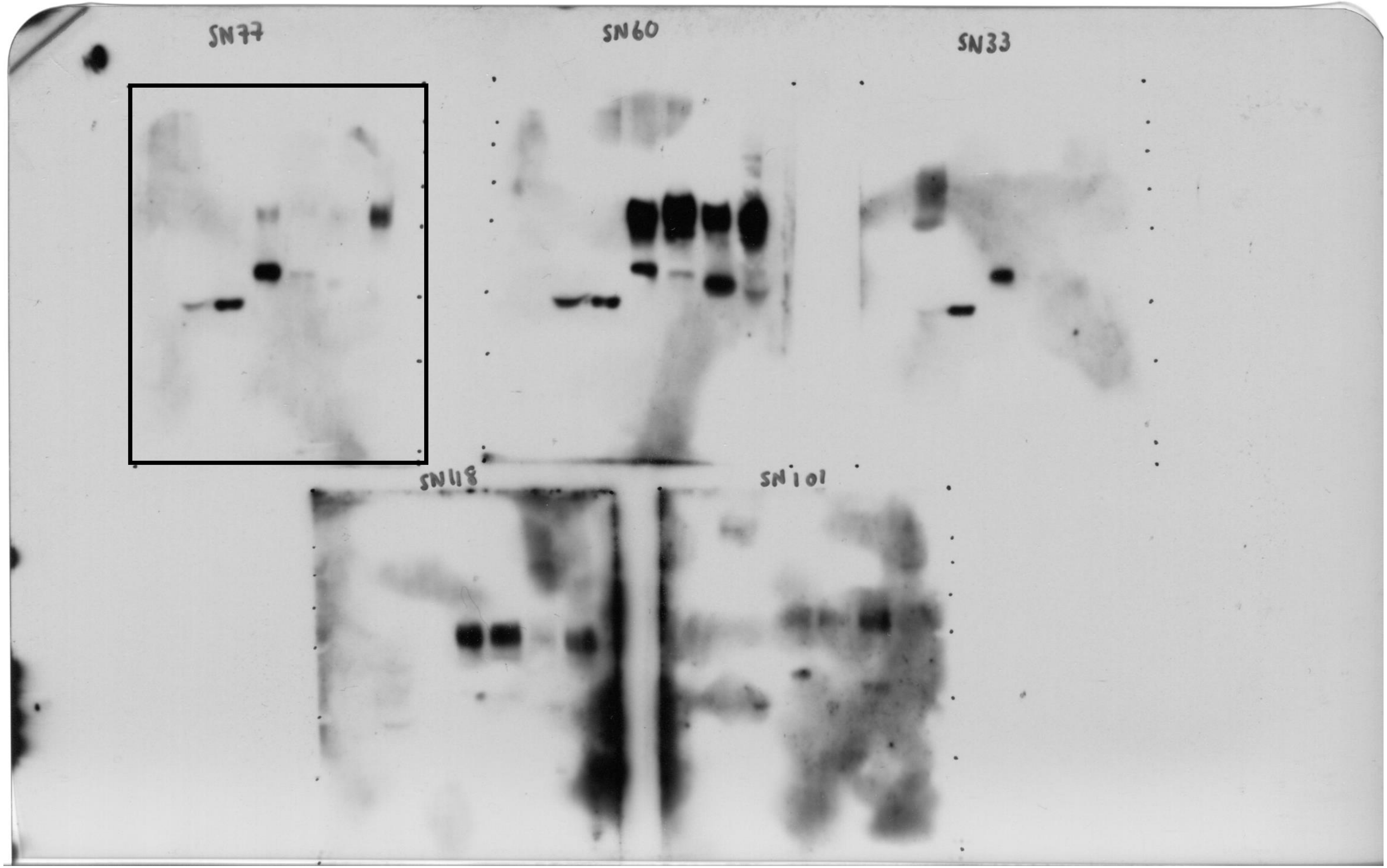

Figure S17

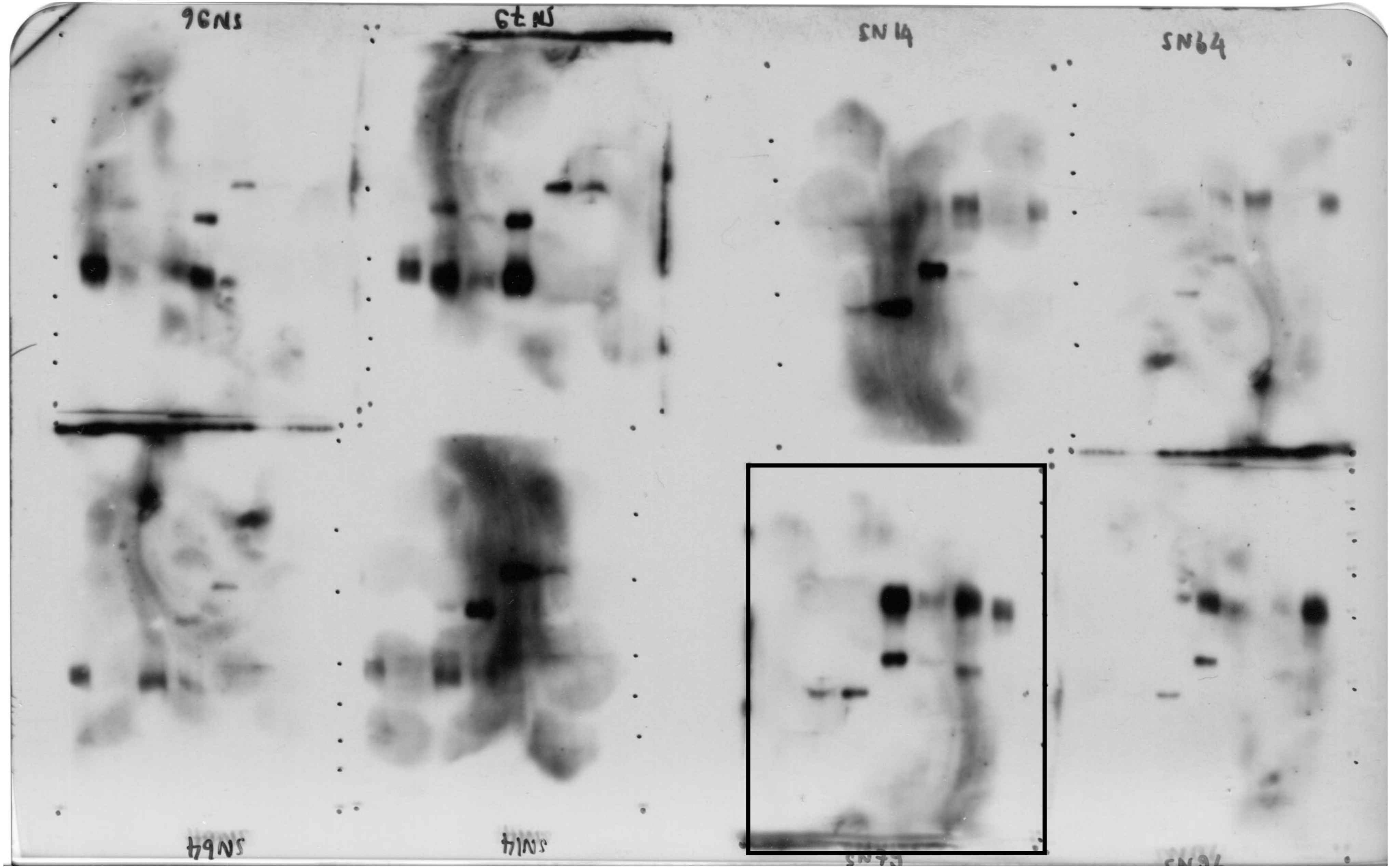

Figure S18

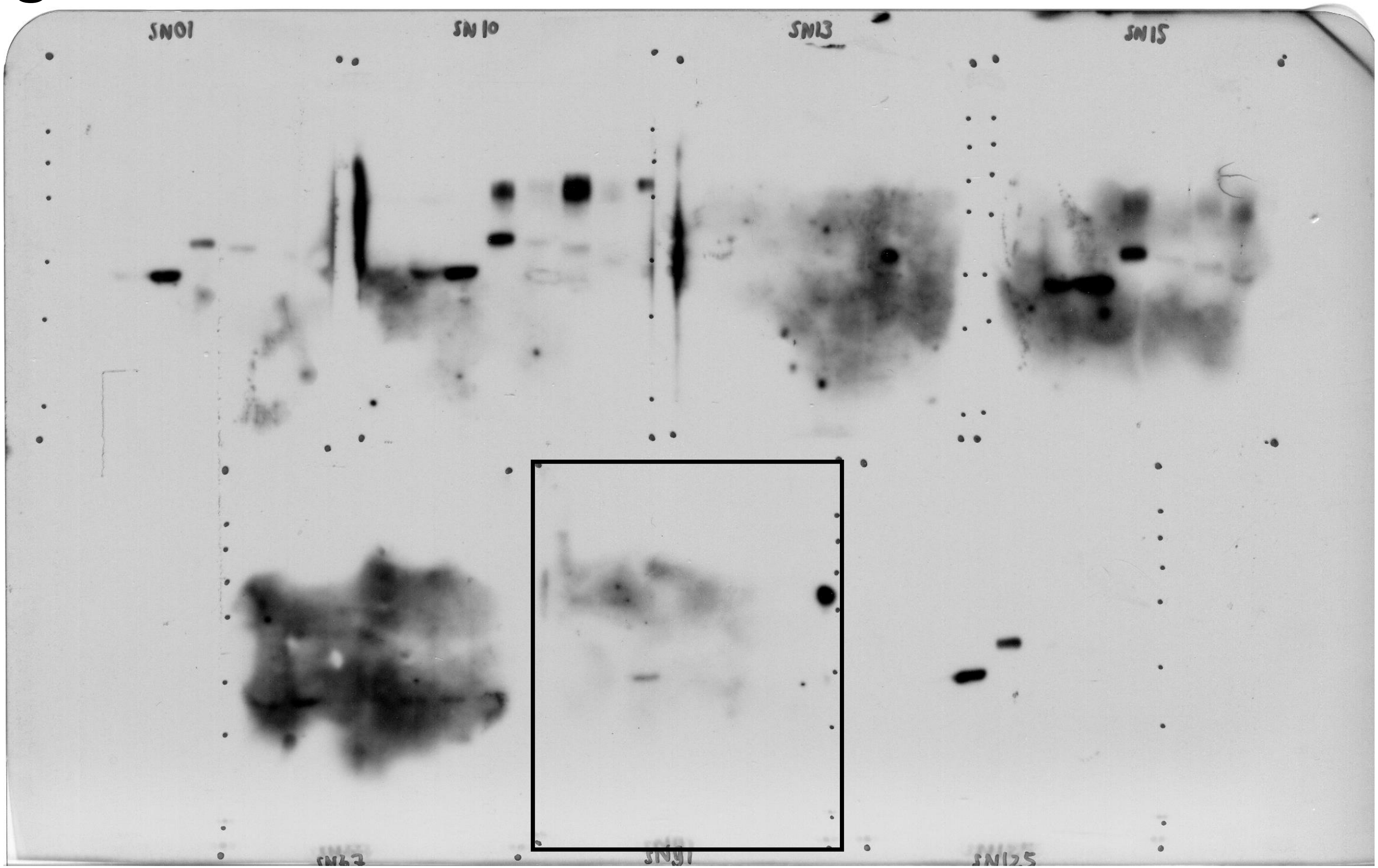

Figure S19

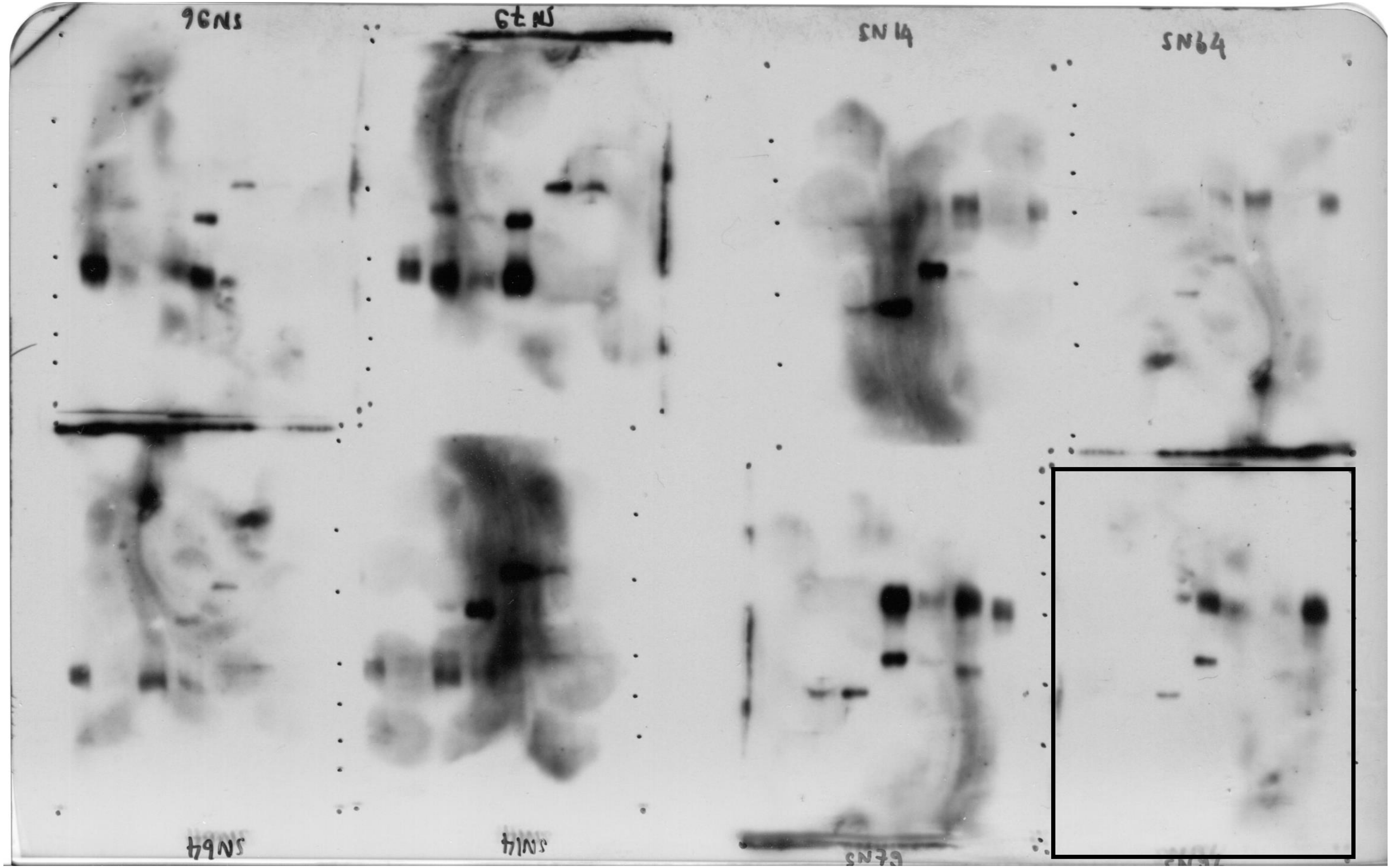

Figure S20

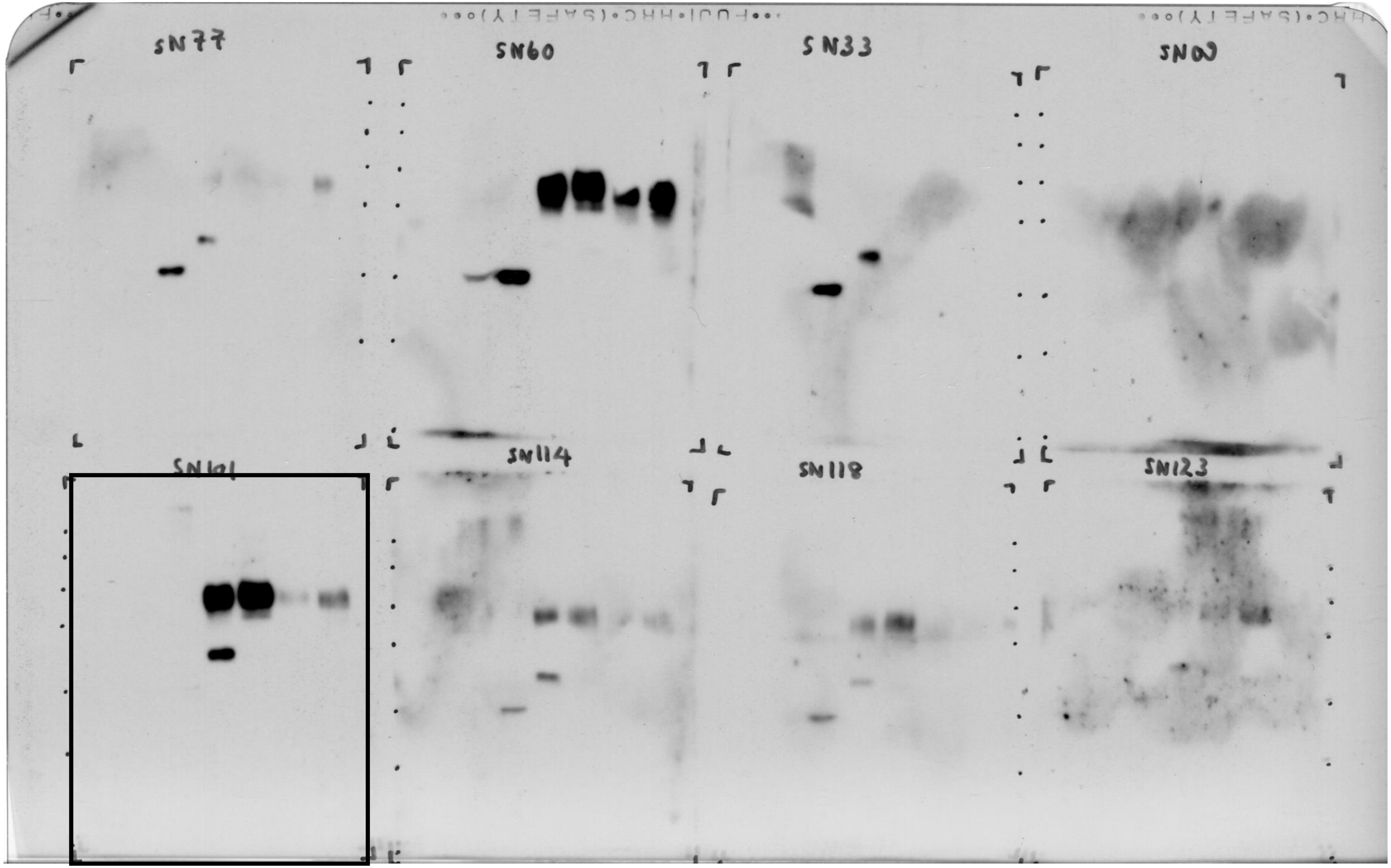

Figure S21

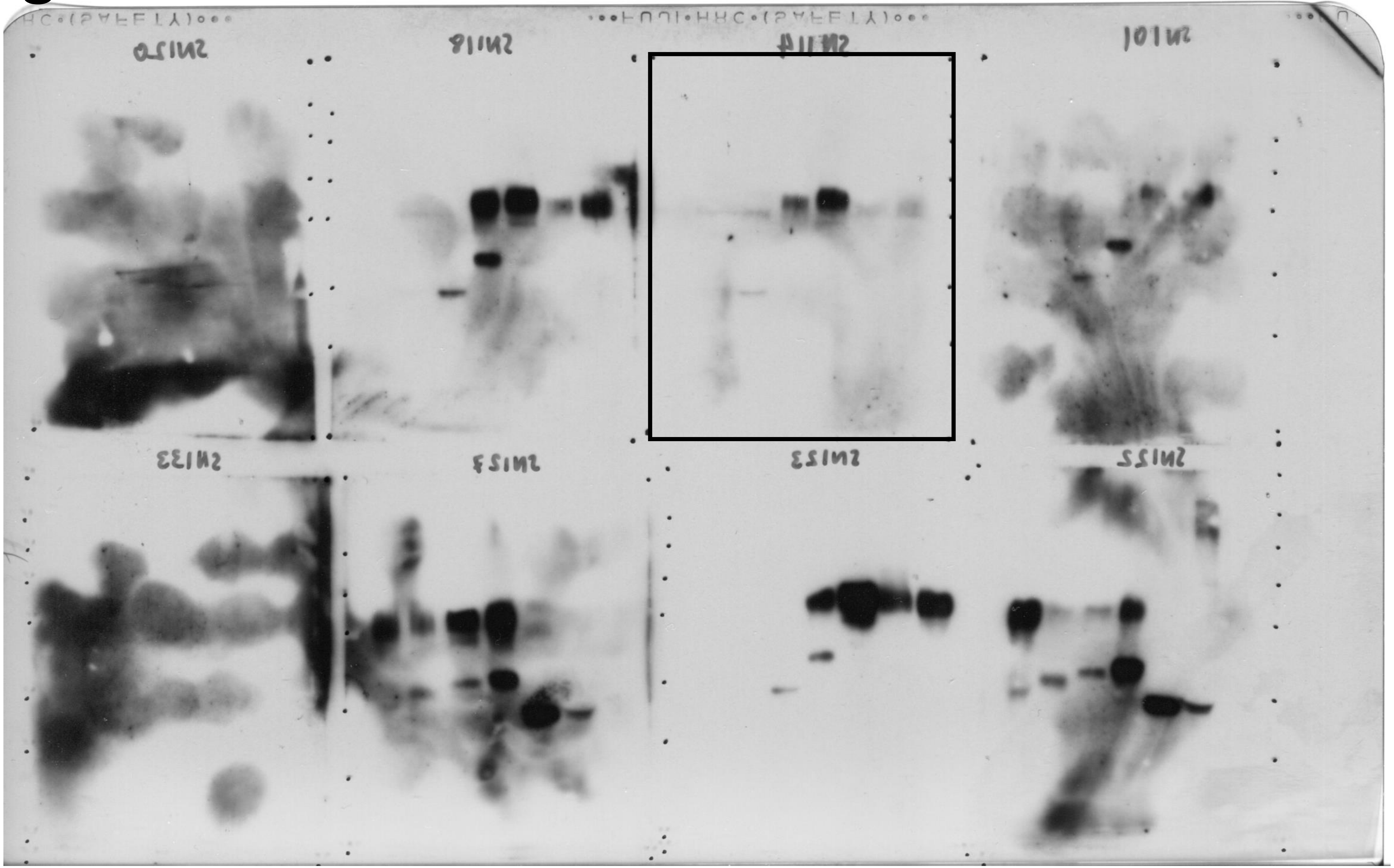

Figure S22

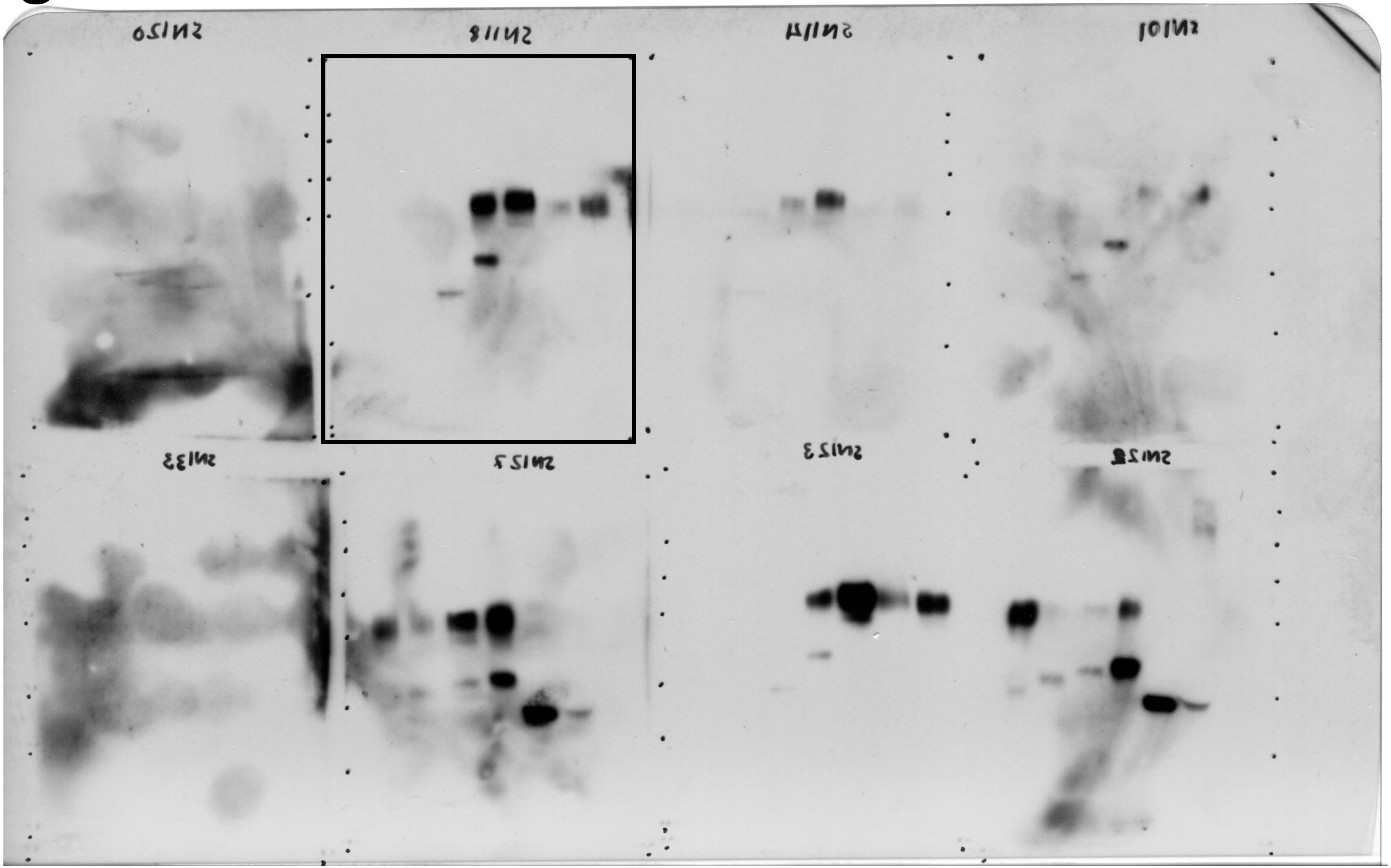

Figure S23

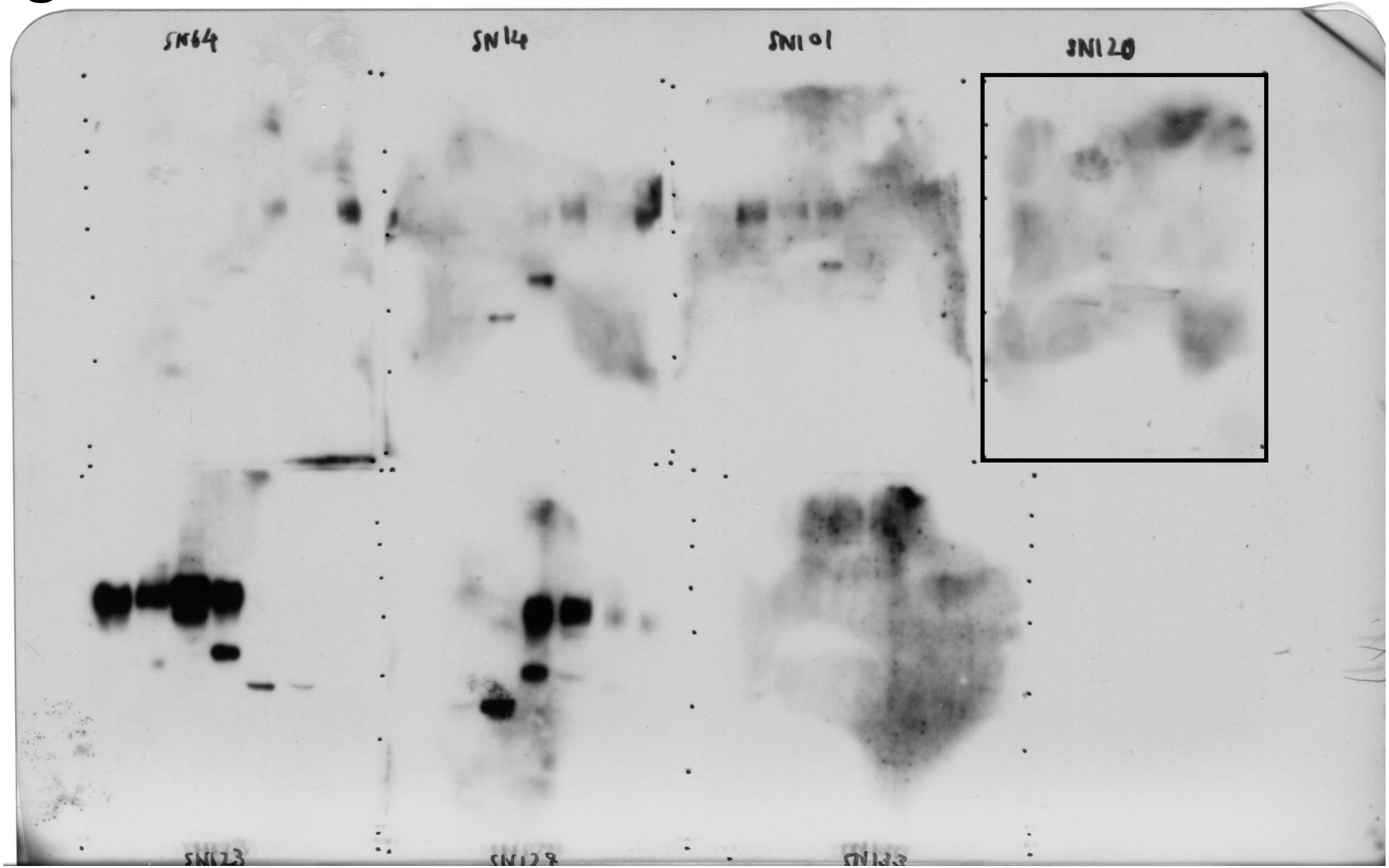

Figure S24

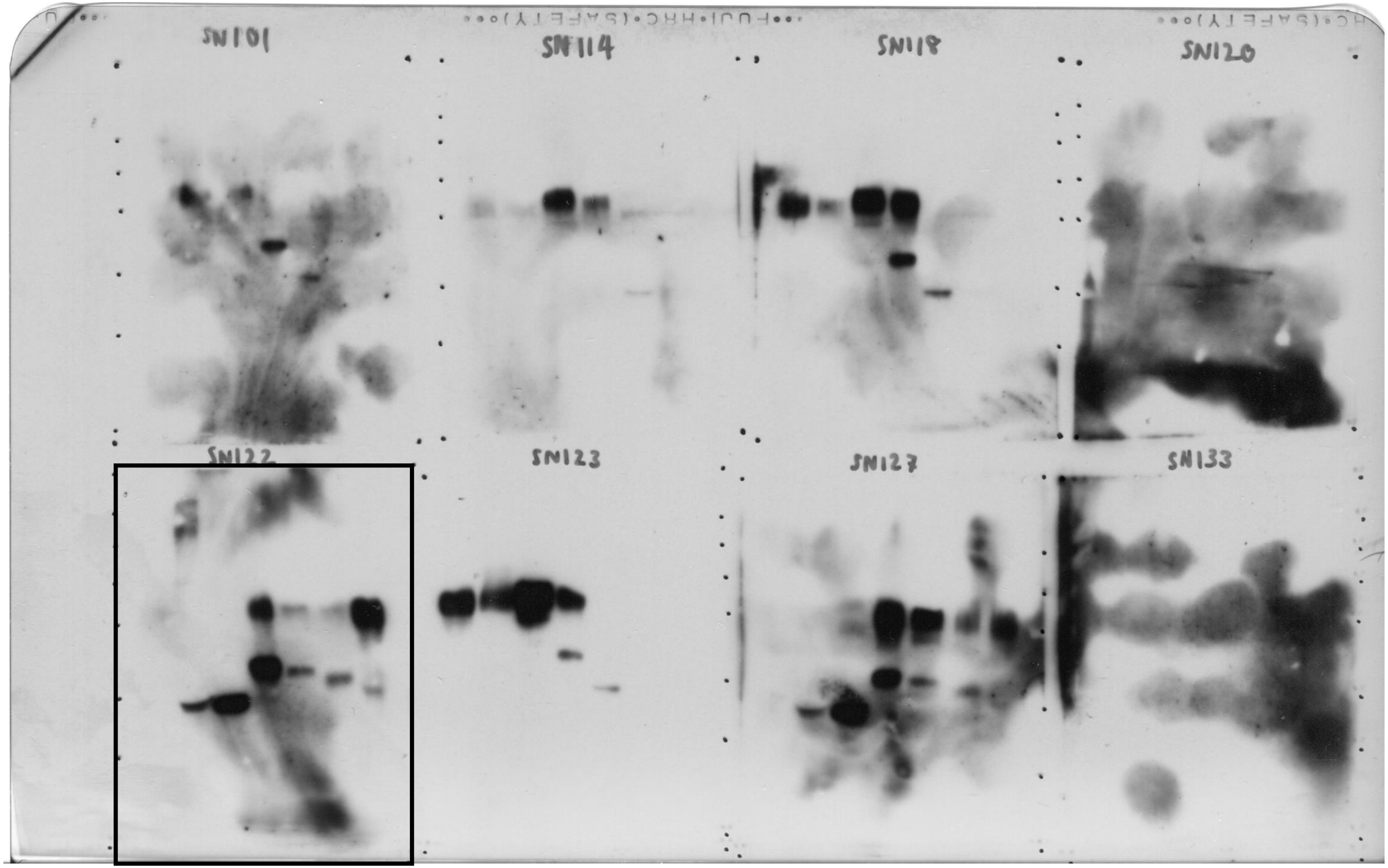

Figure S25

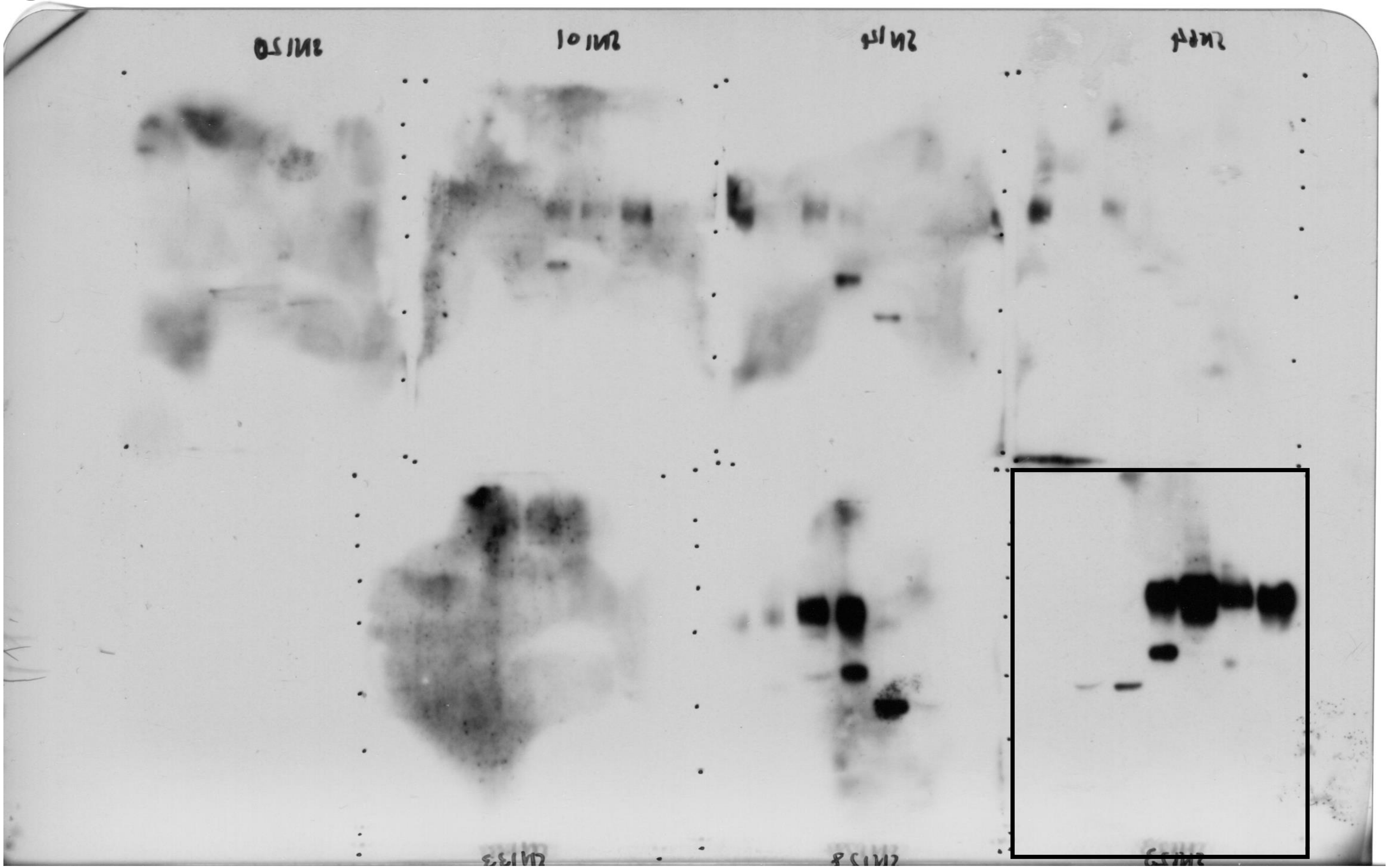

Figure S26

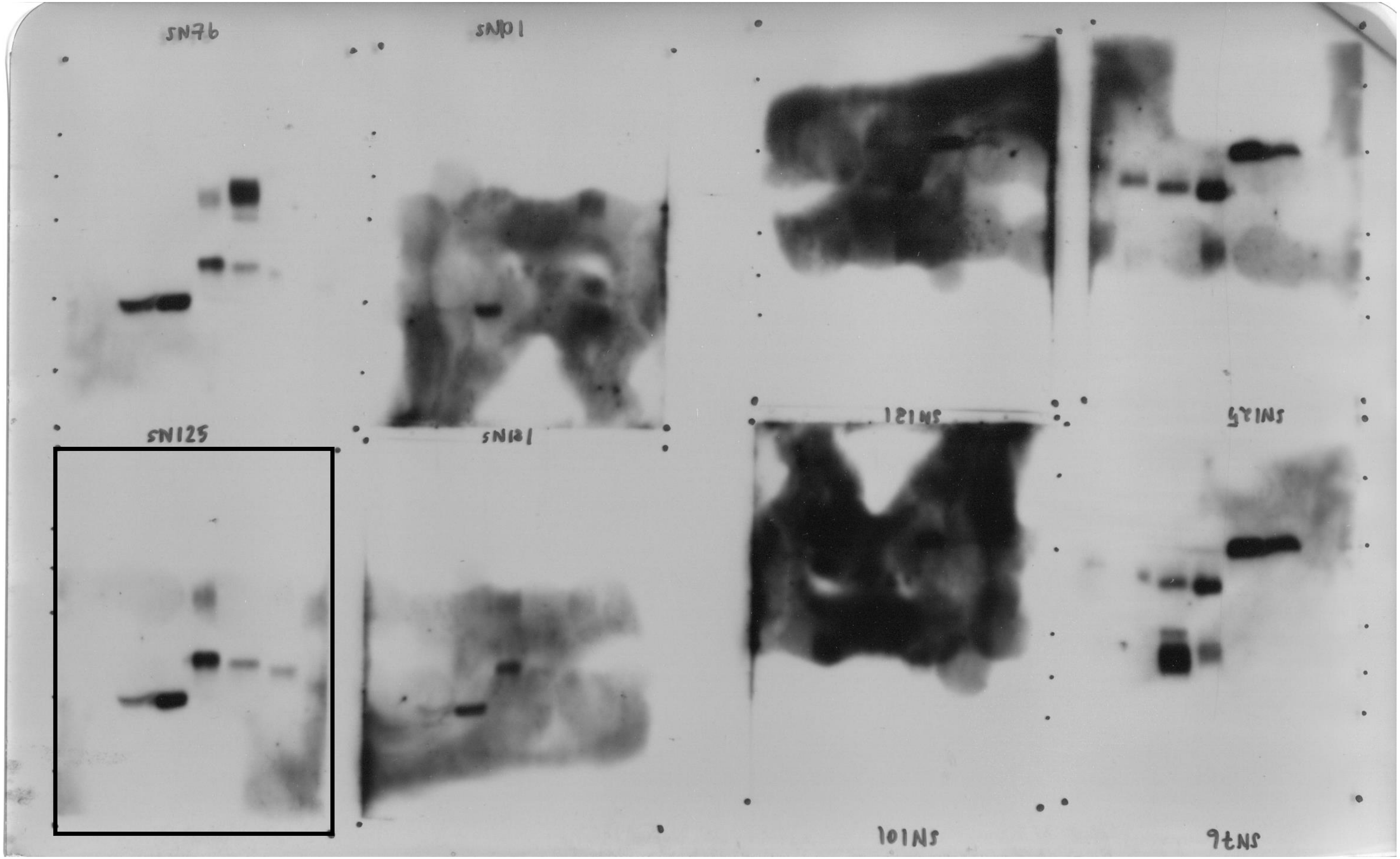

Figure S27

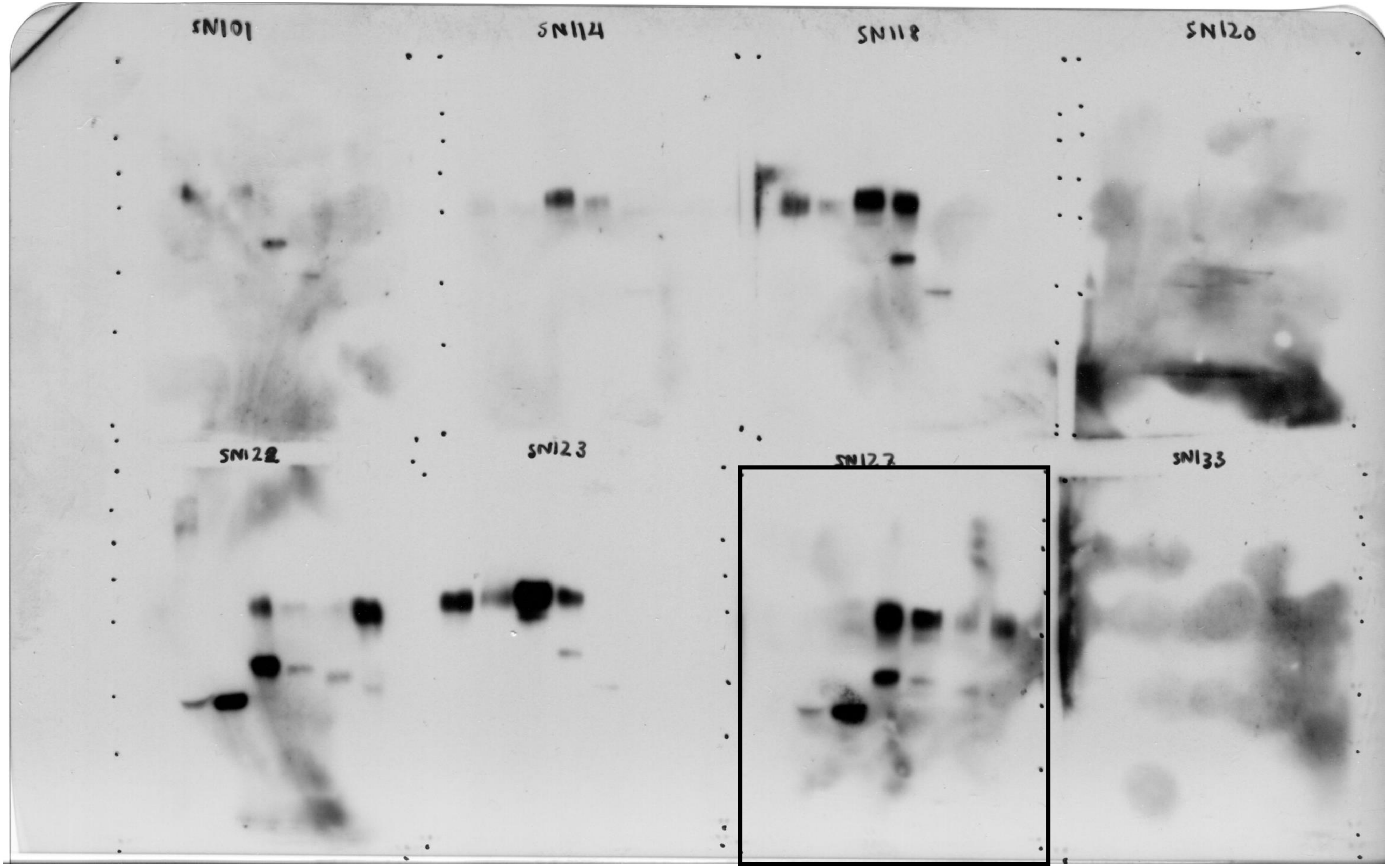

Figure S28

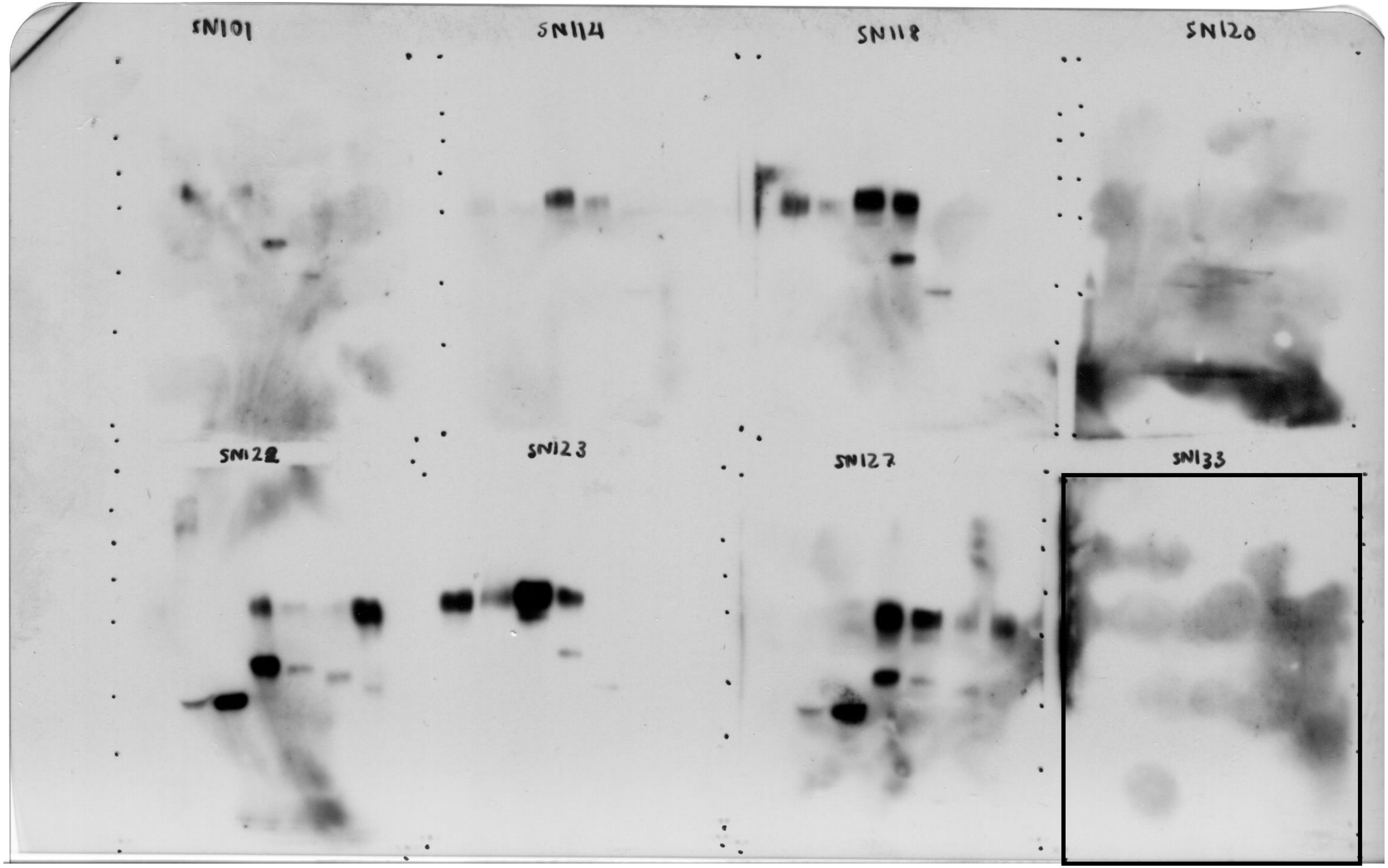

Figure S29

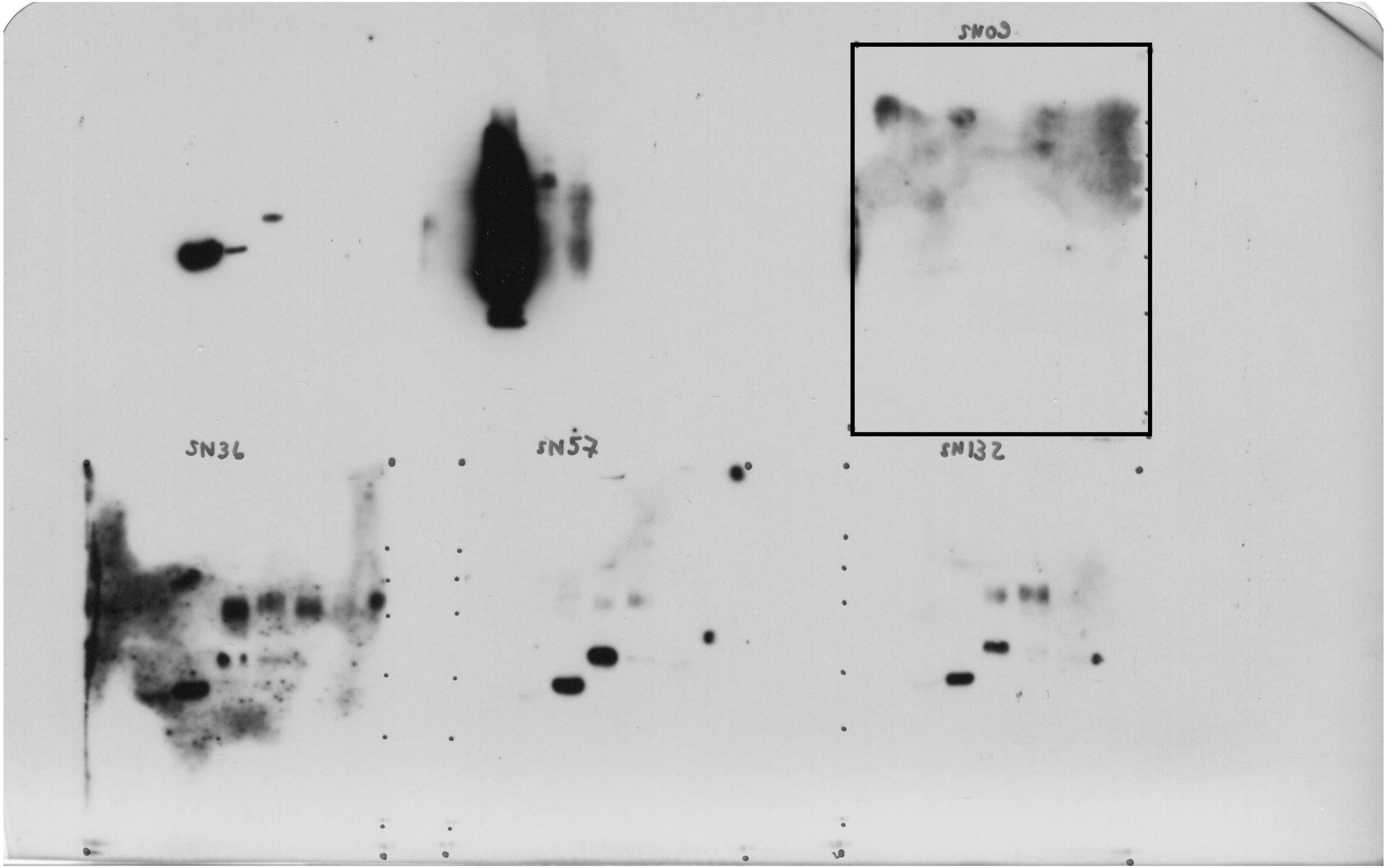

Figure S30

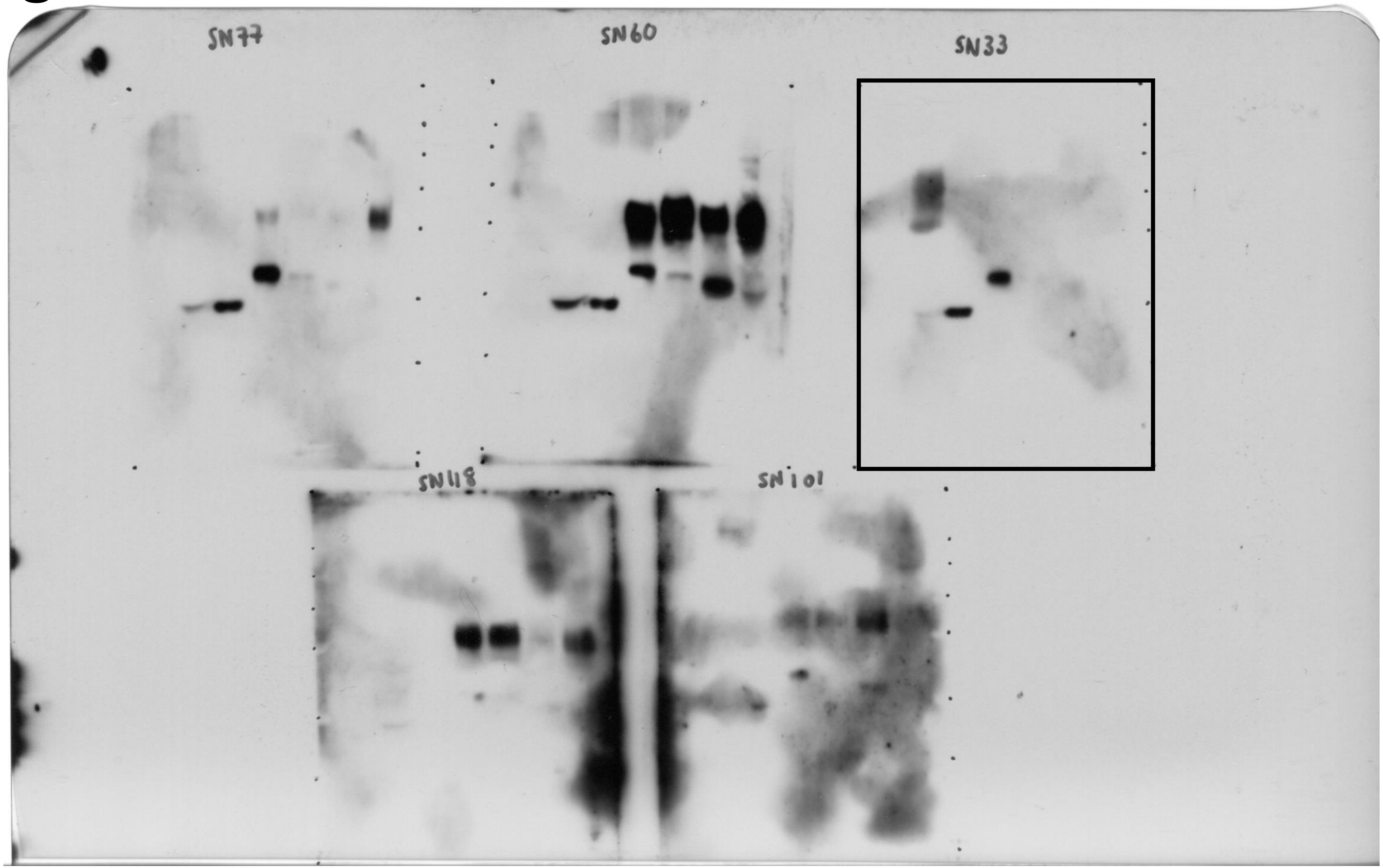

Figure S31

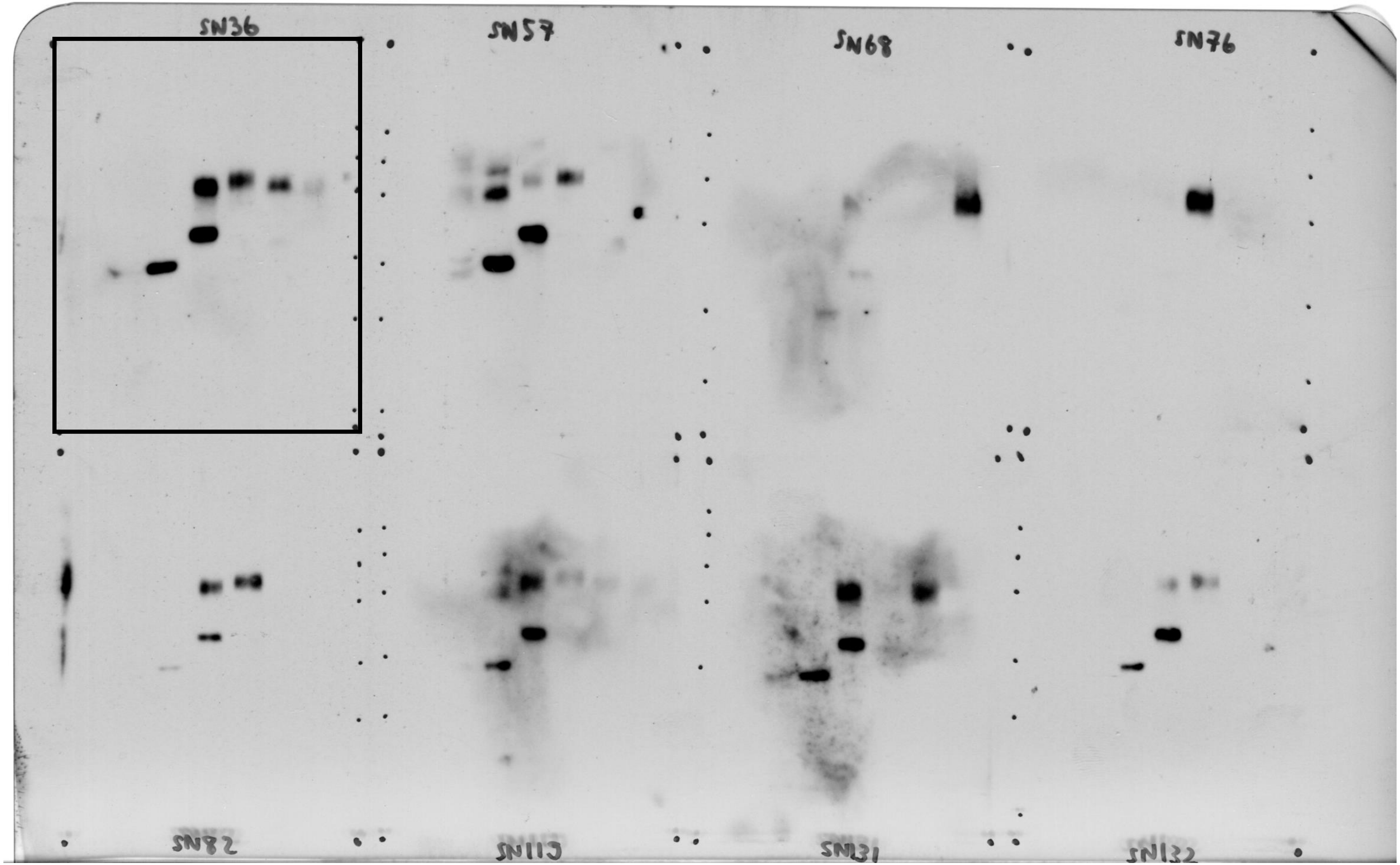

Figure S32

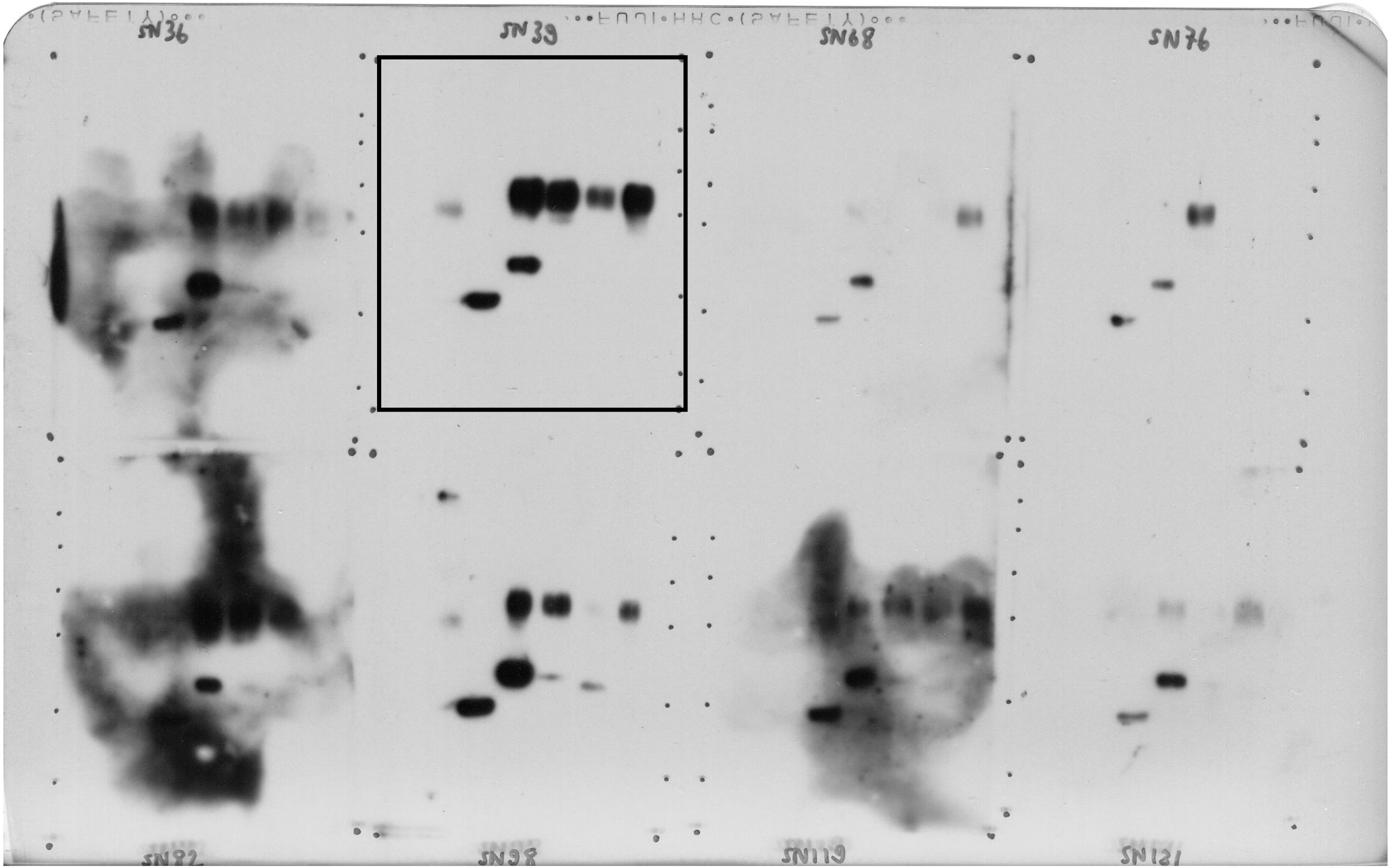

Figure S33

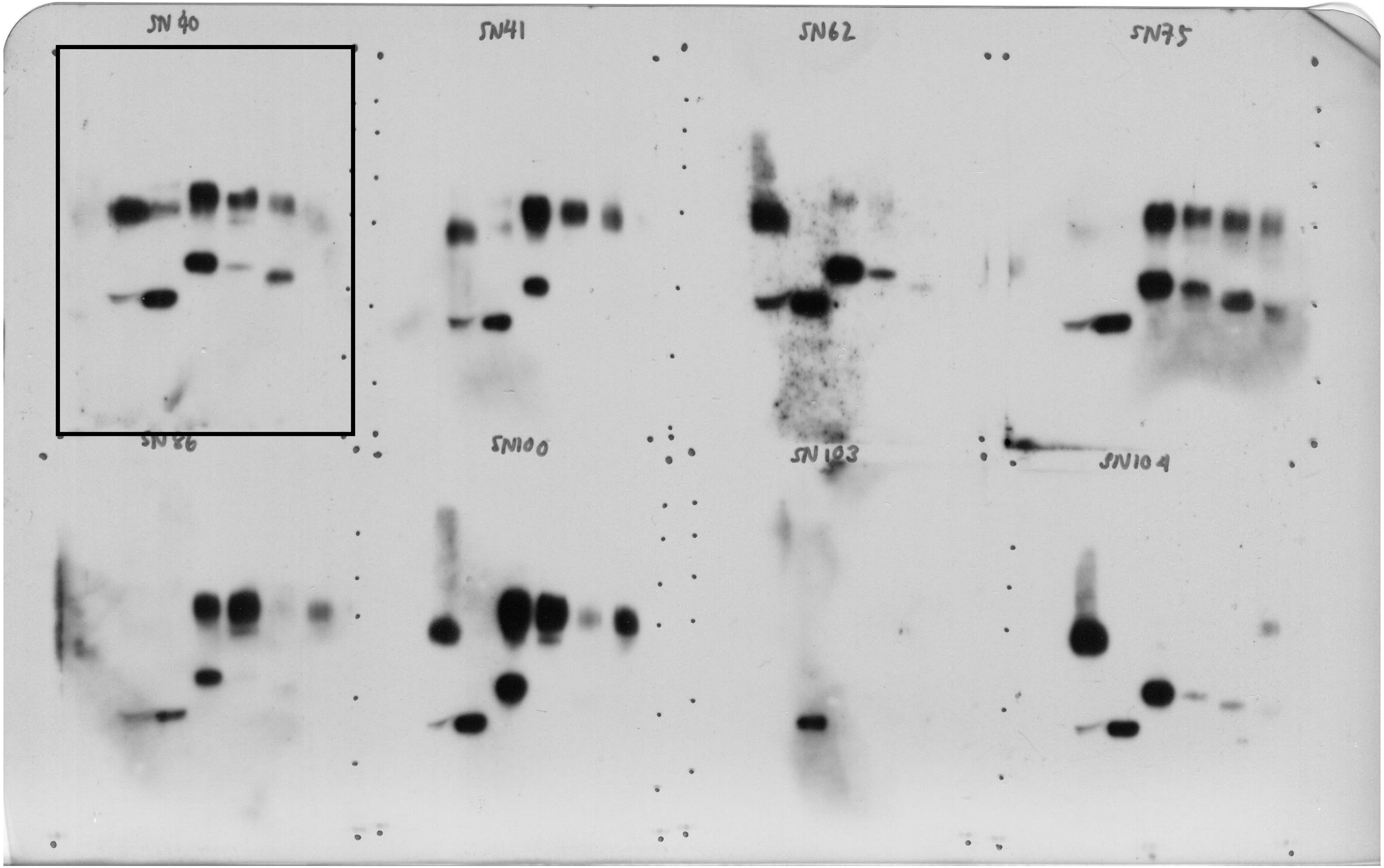

Figure S34

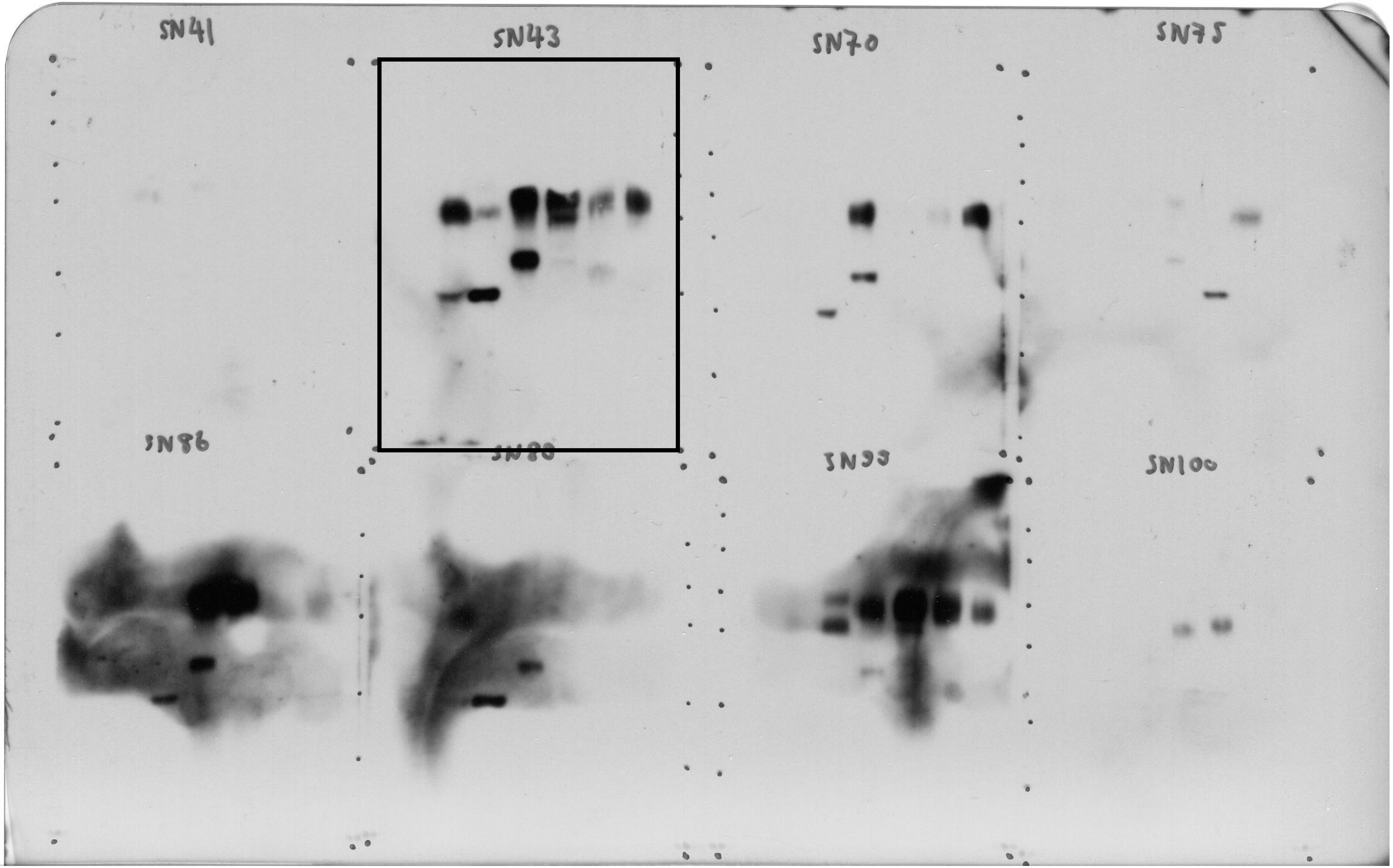

Figure S35

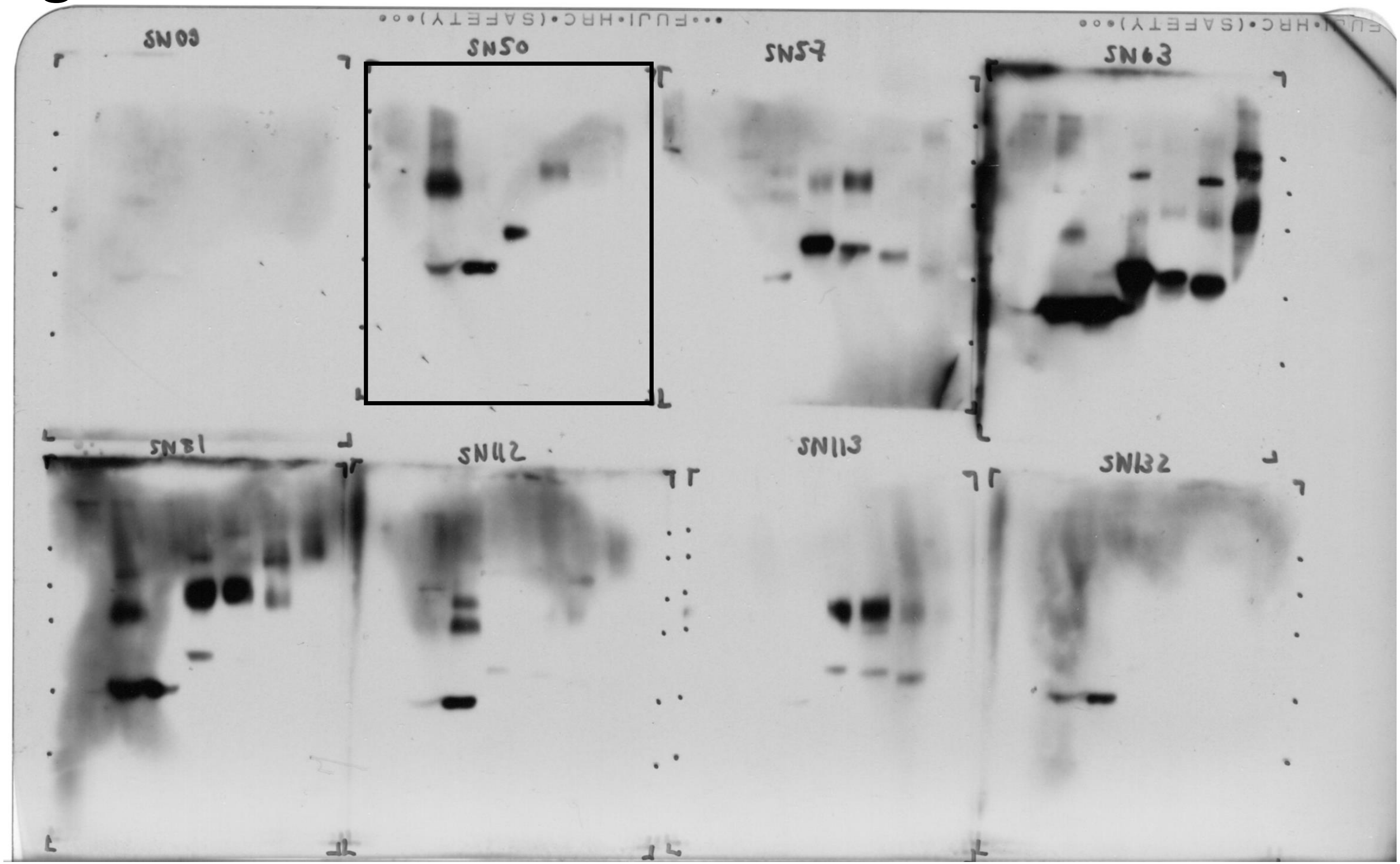

Figure S36

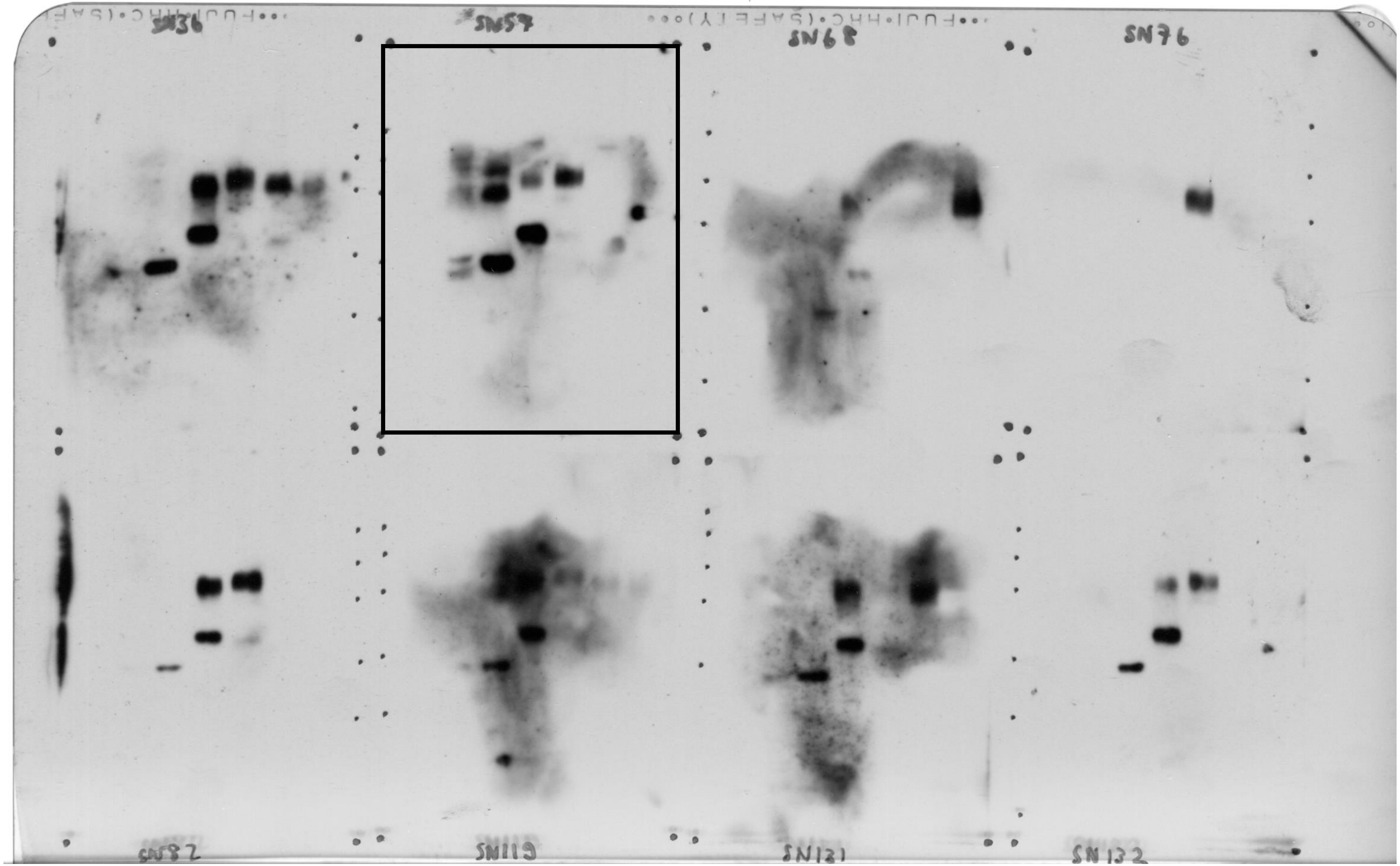

Figure S37

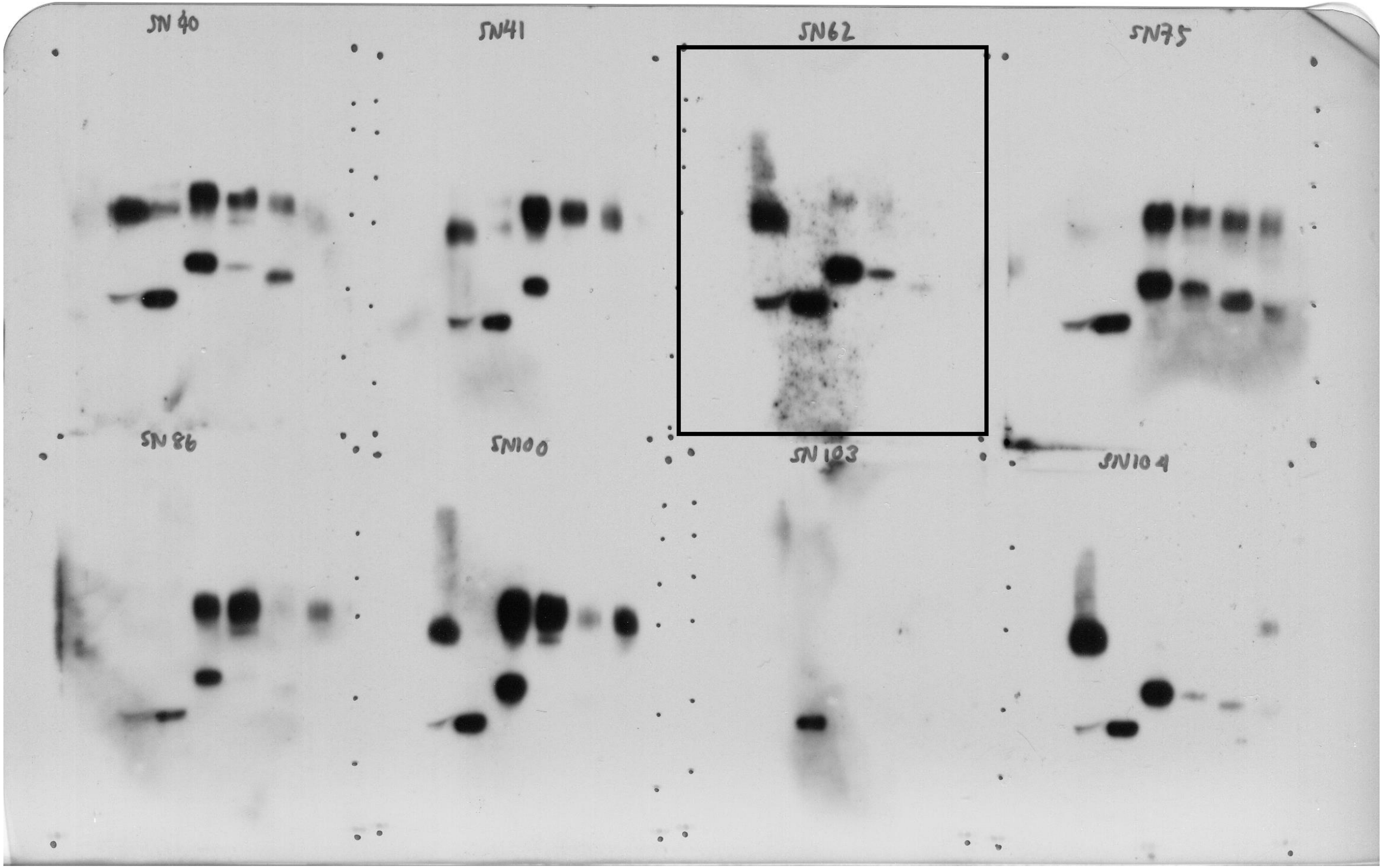

Figure S38

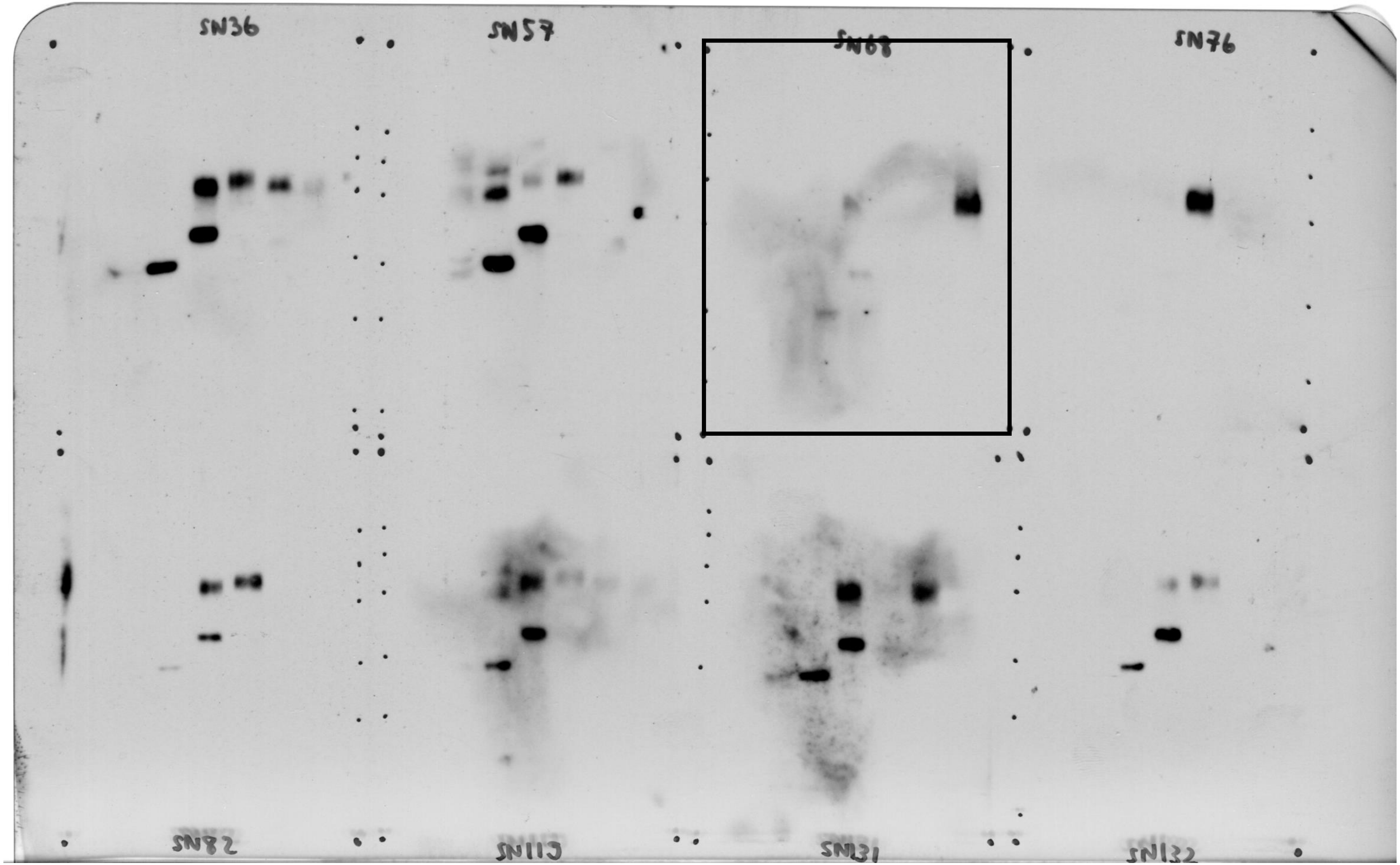

Figure S39

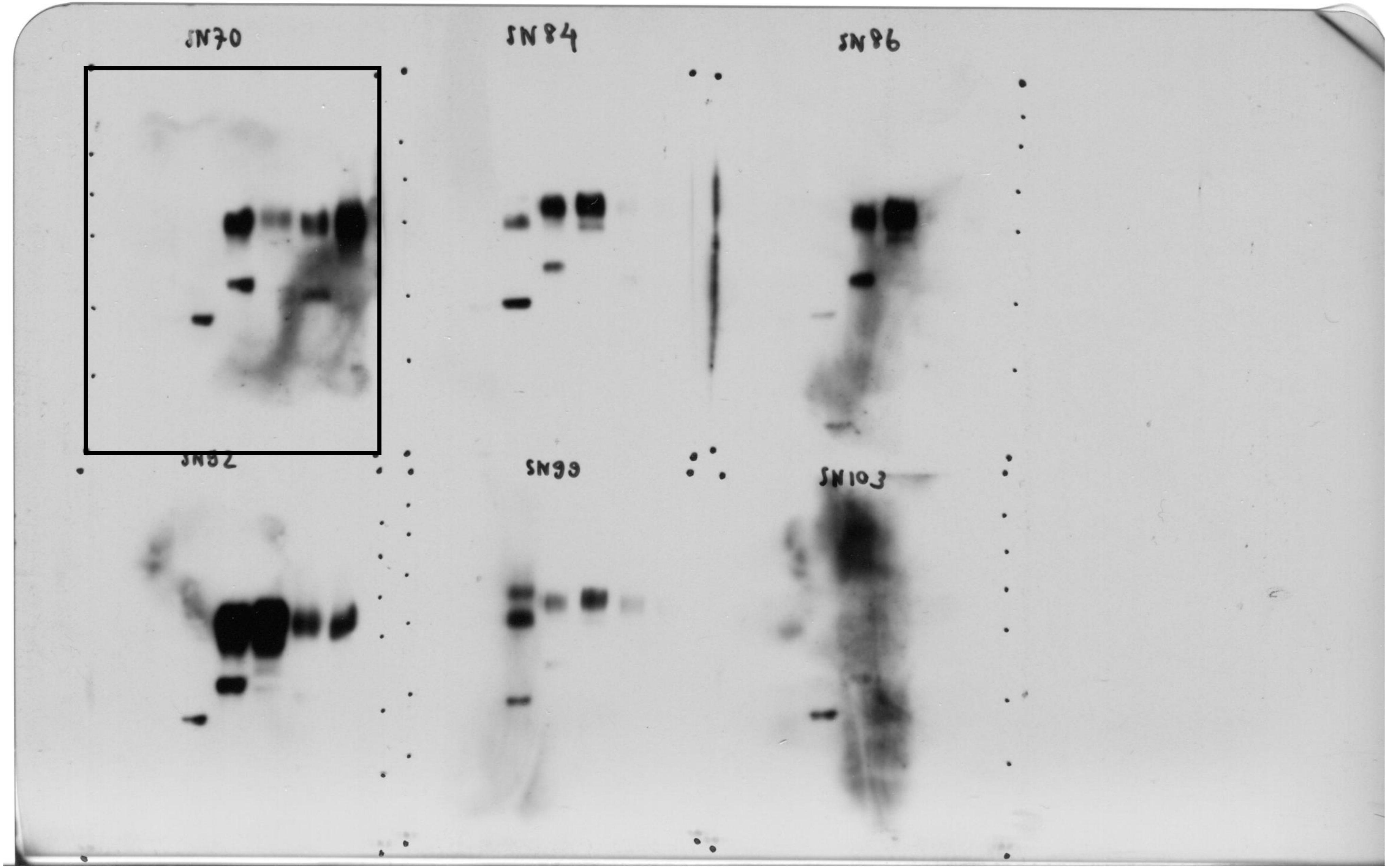

Figure S40

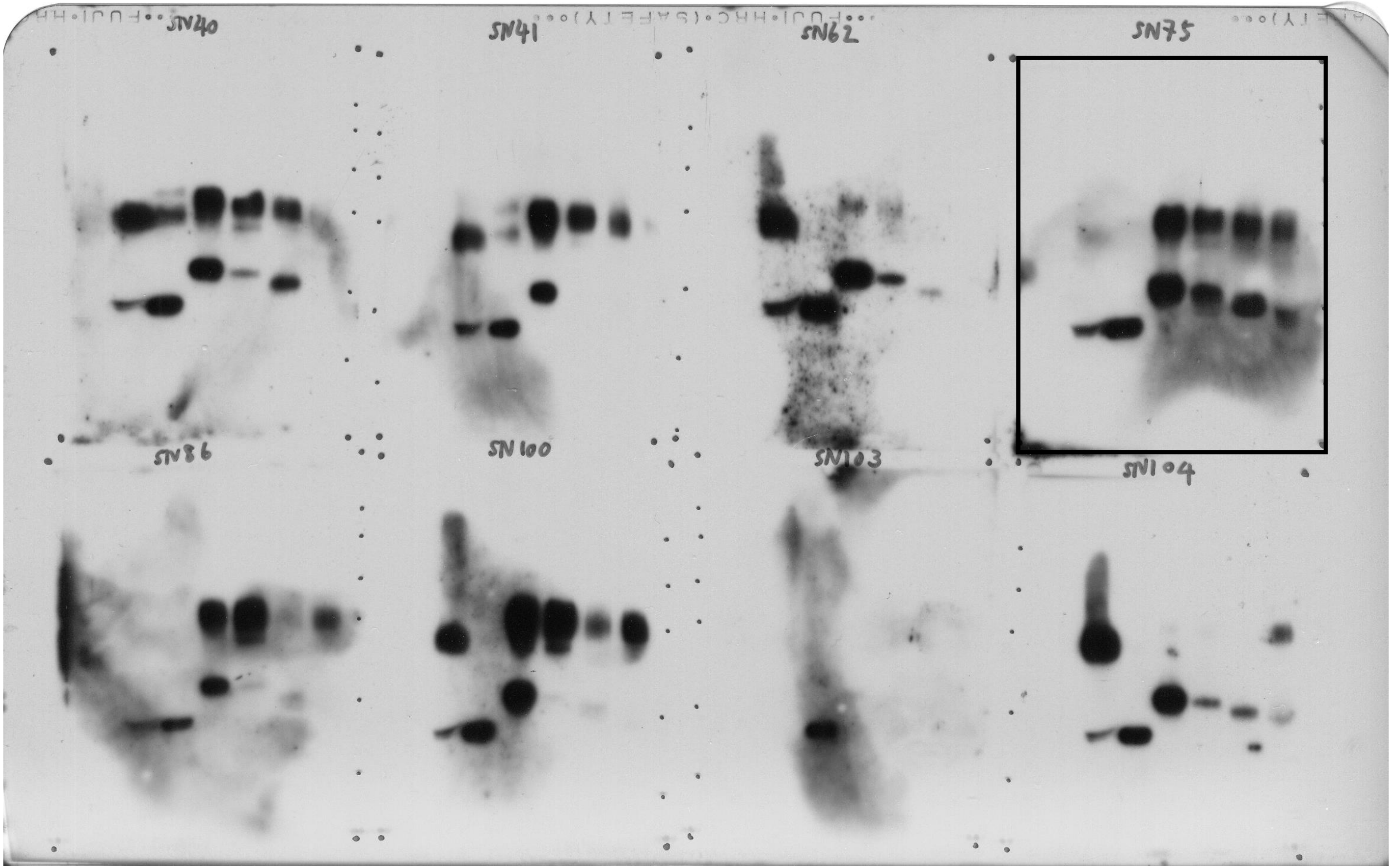

Figure S41

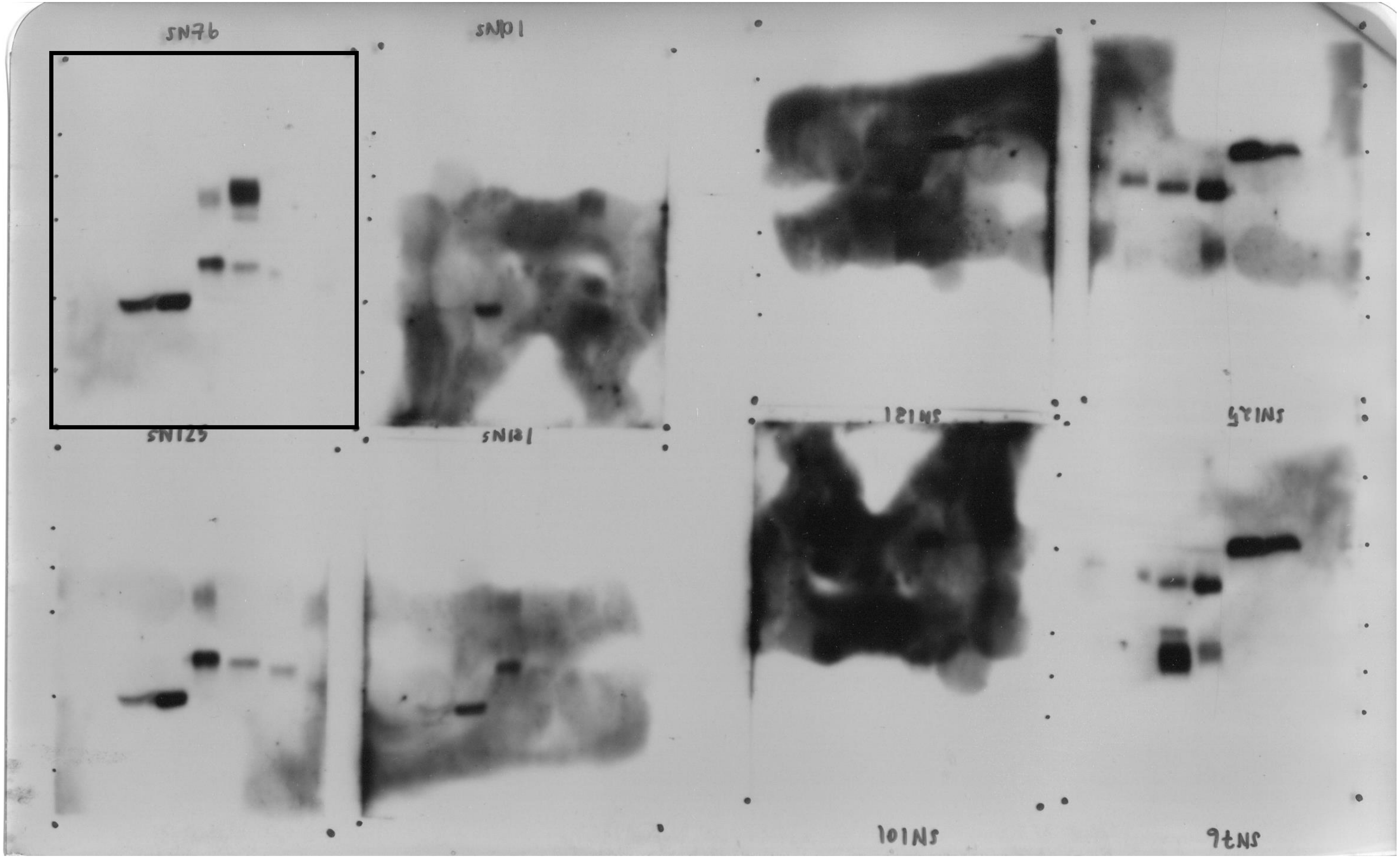

Figure S42

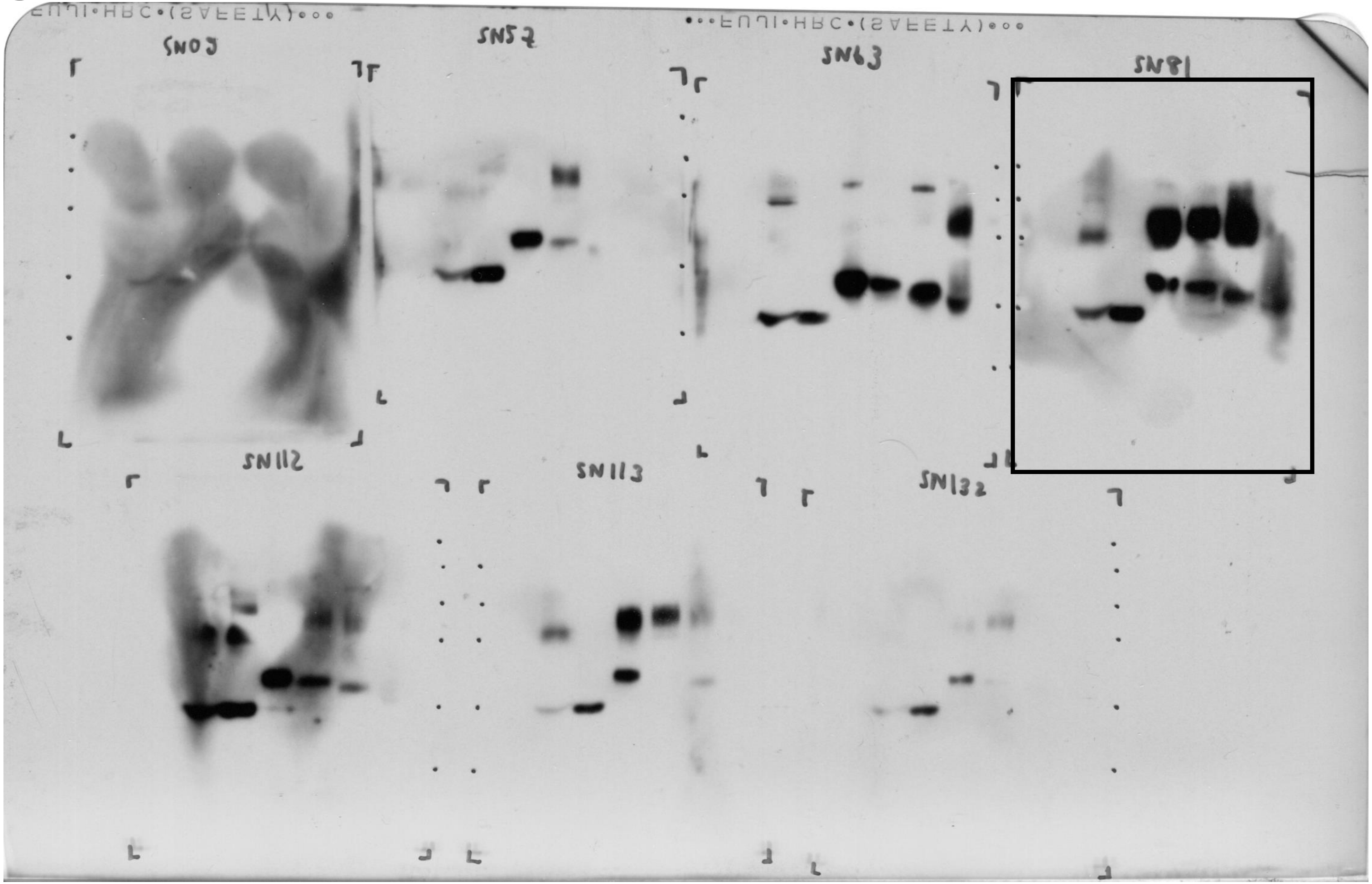

Figure S43

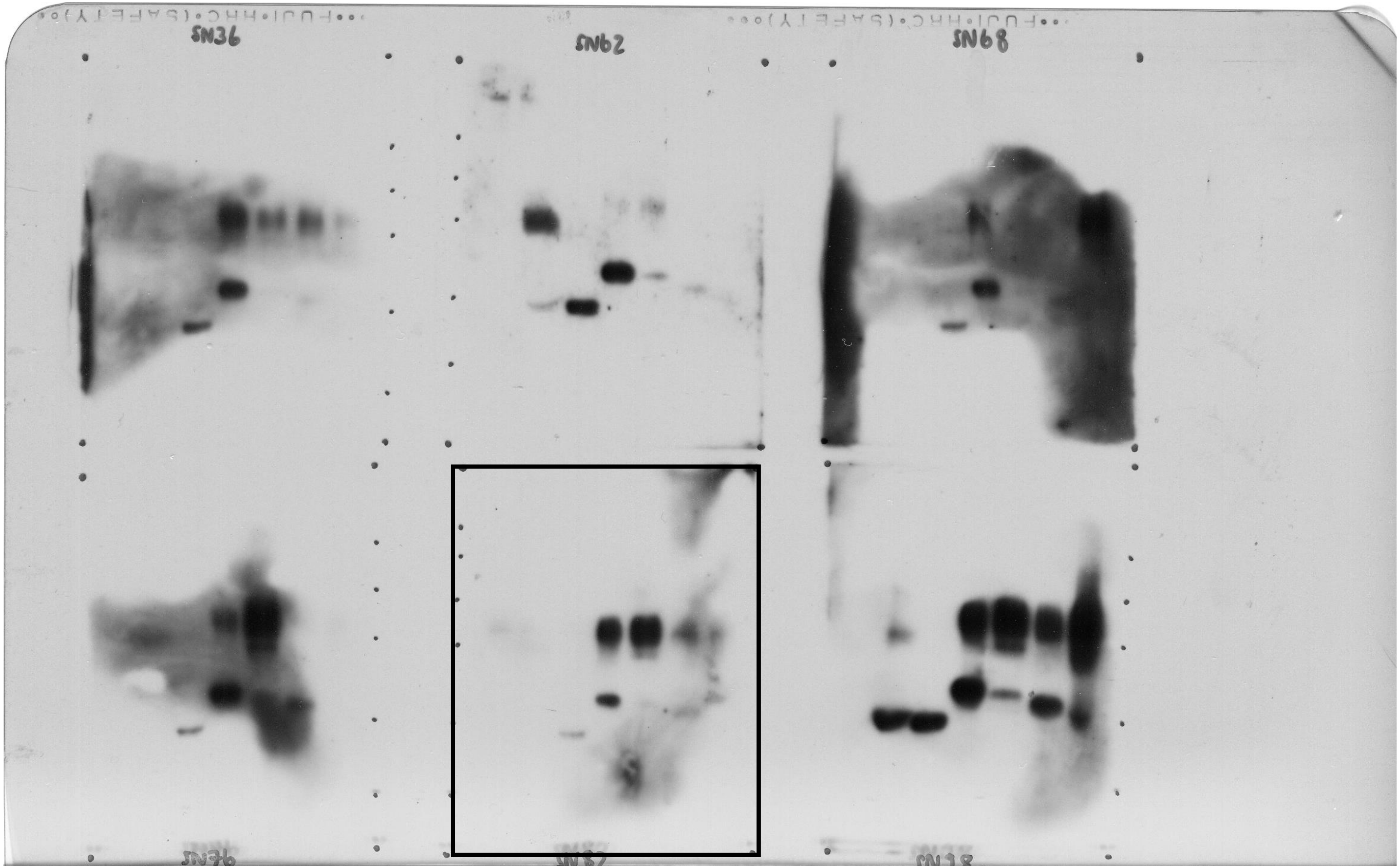

Figure S44

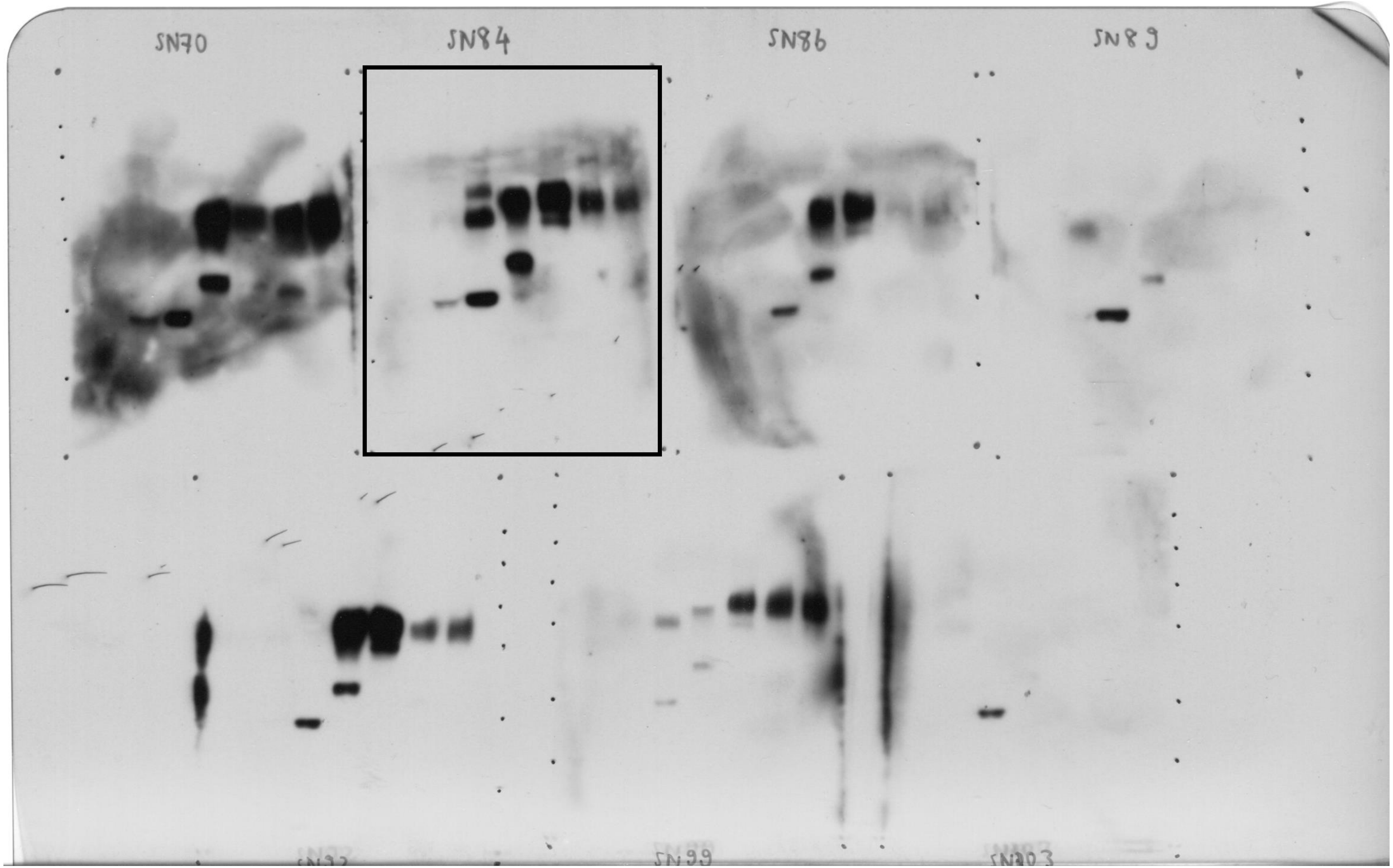

Figure S45

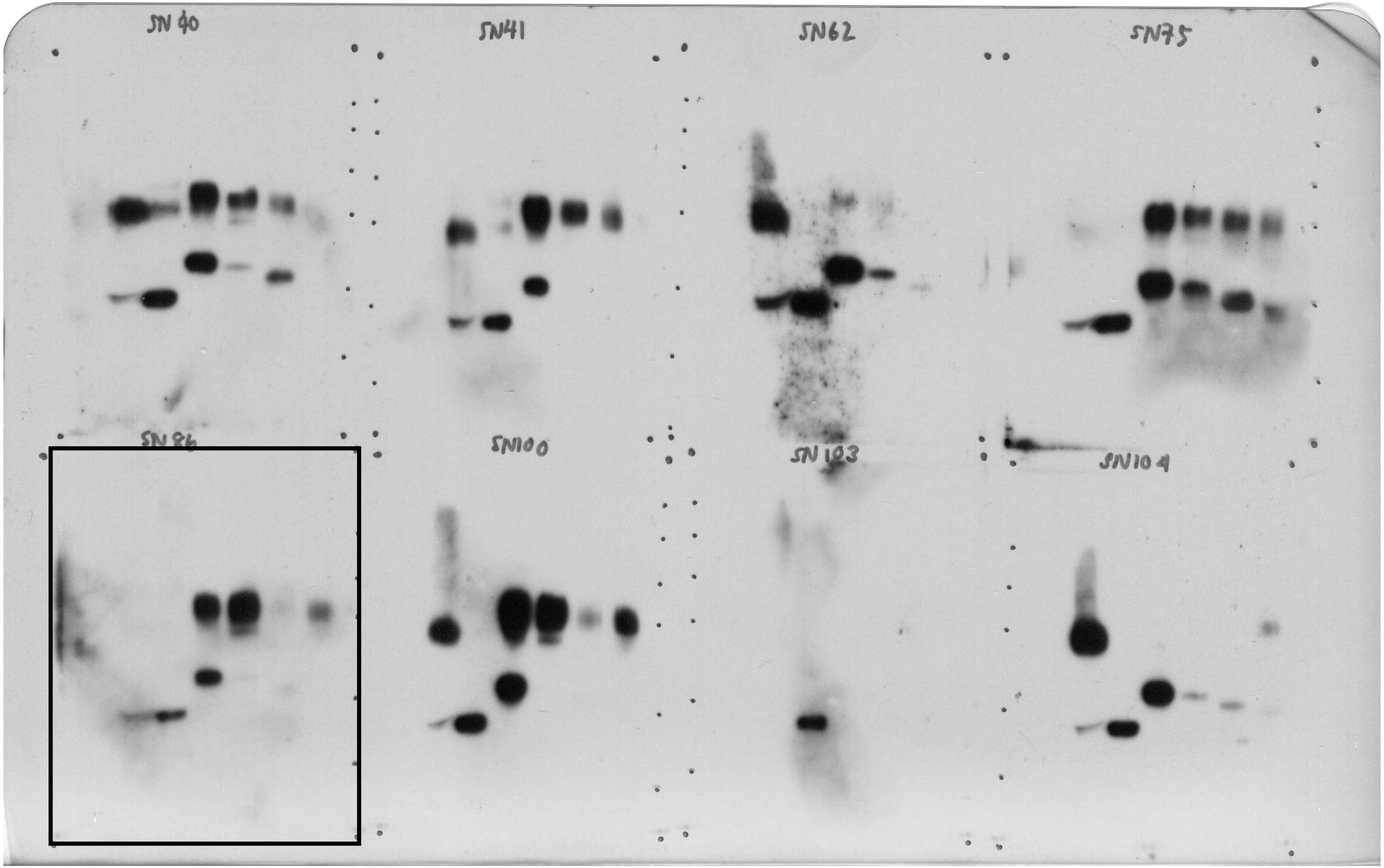

Figure S46

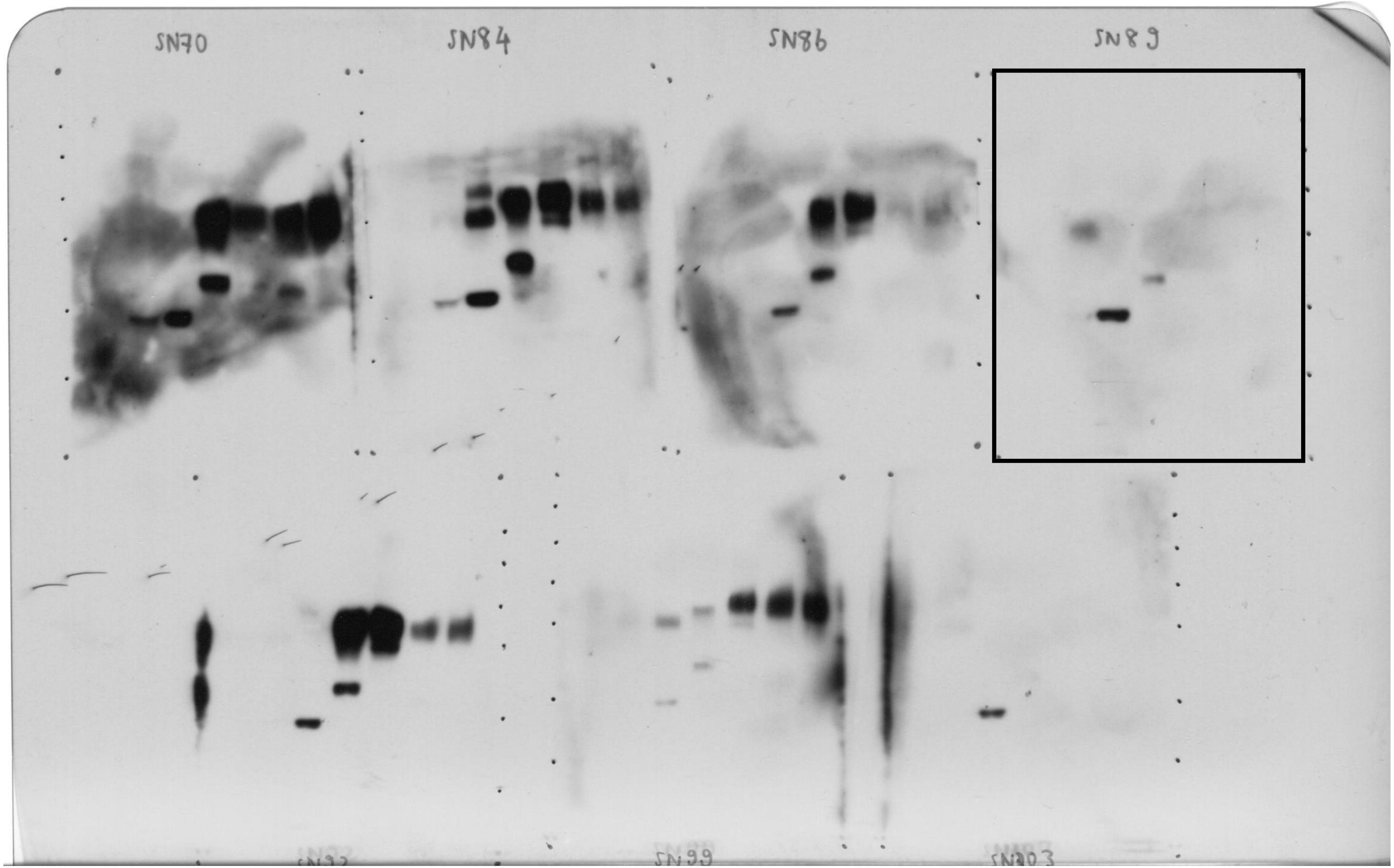

Figure S47

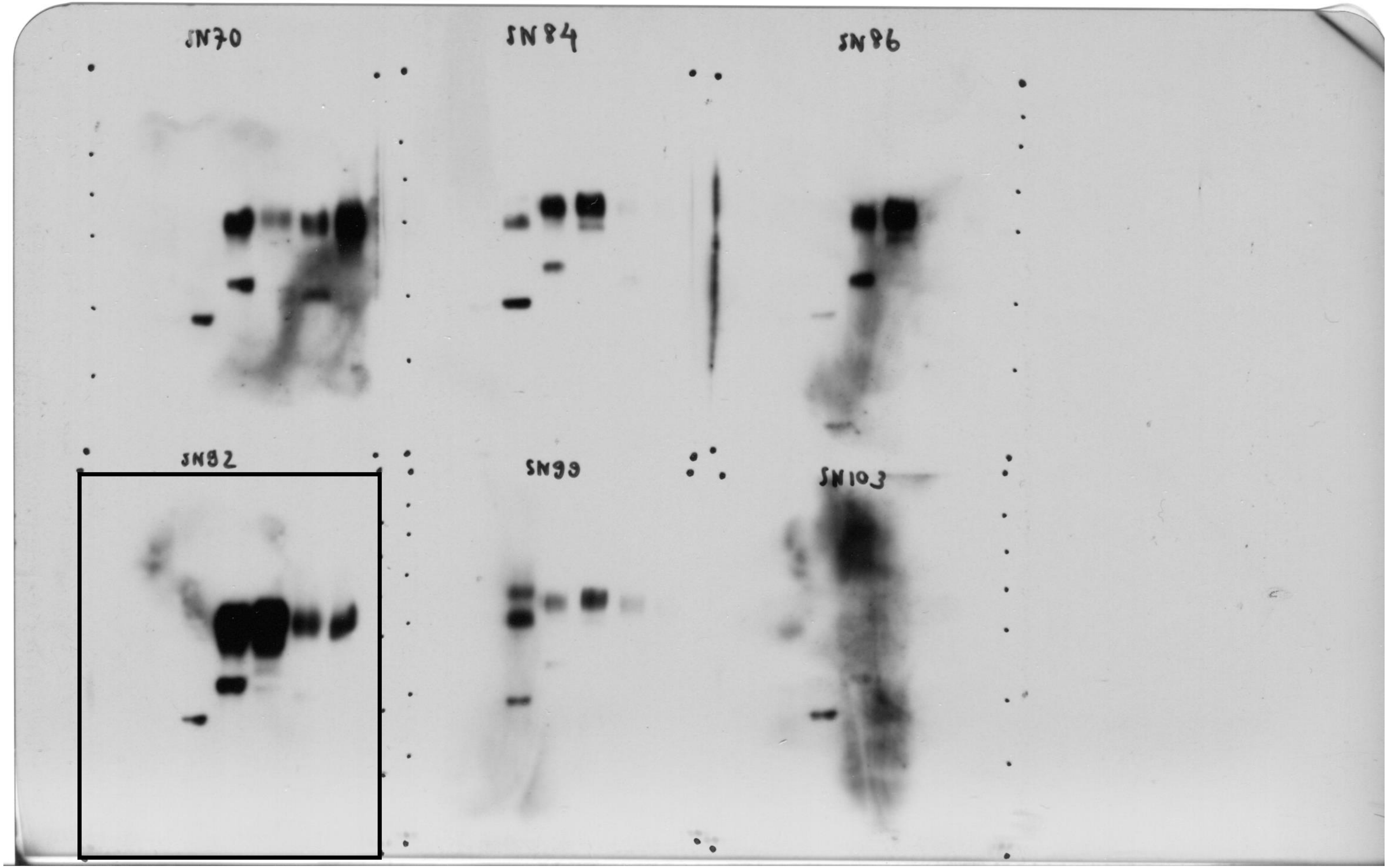

Figure S48

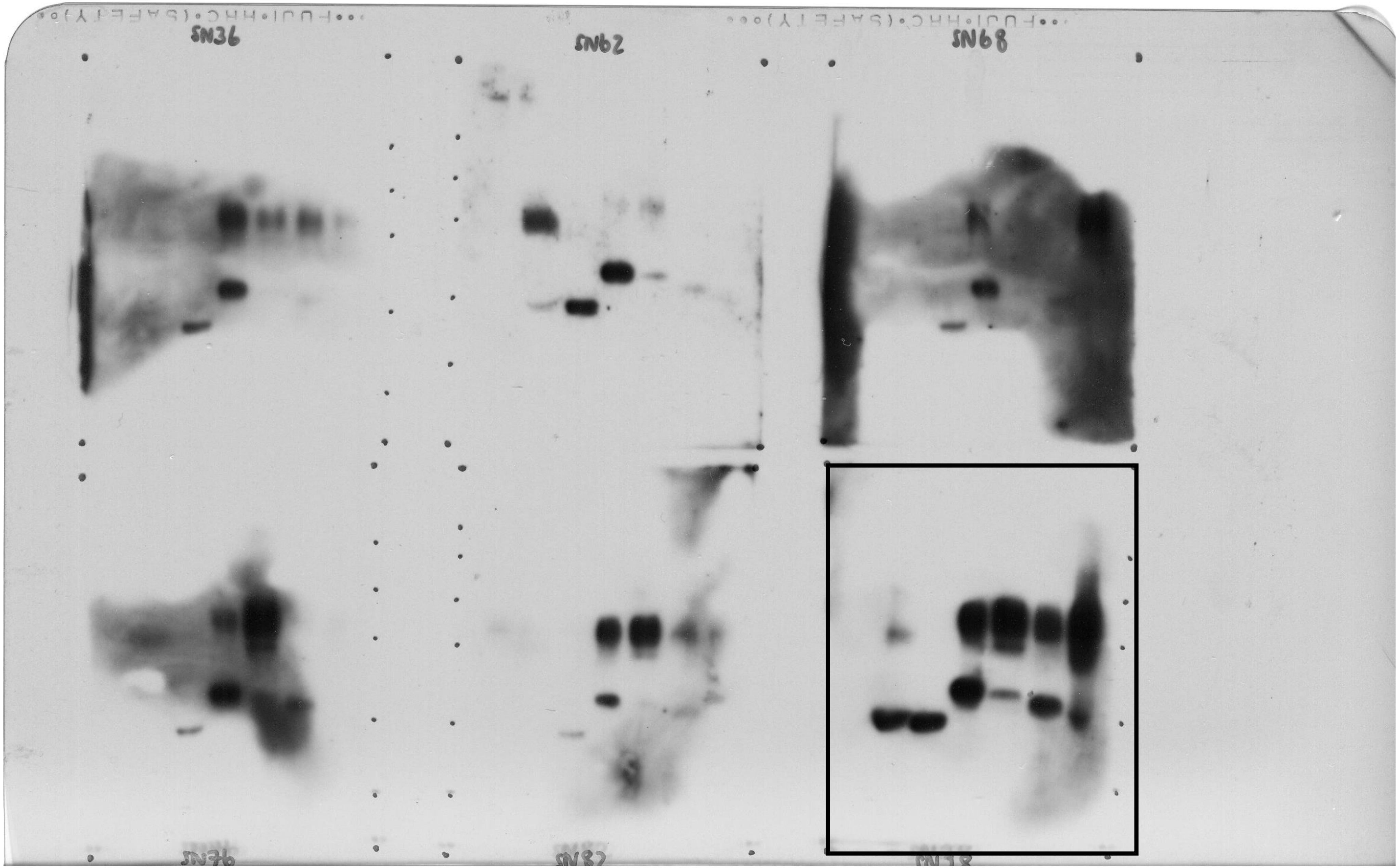

Figure S49

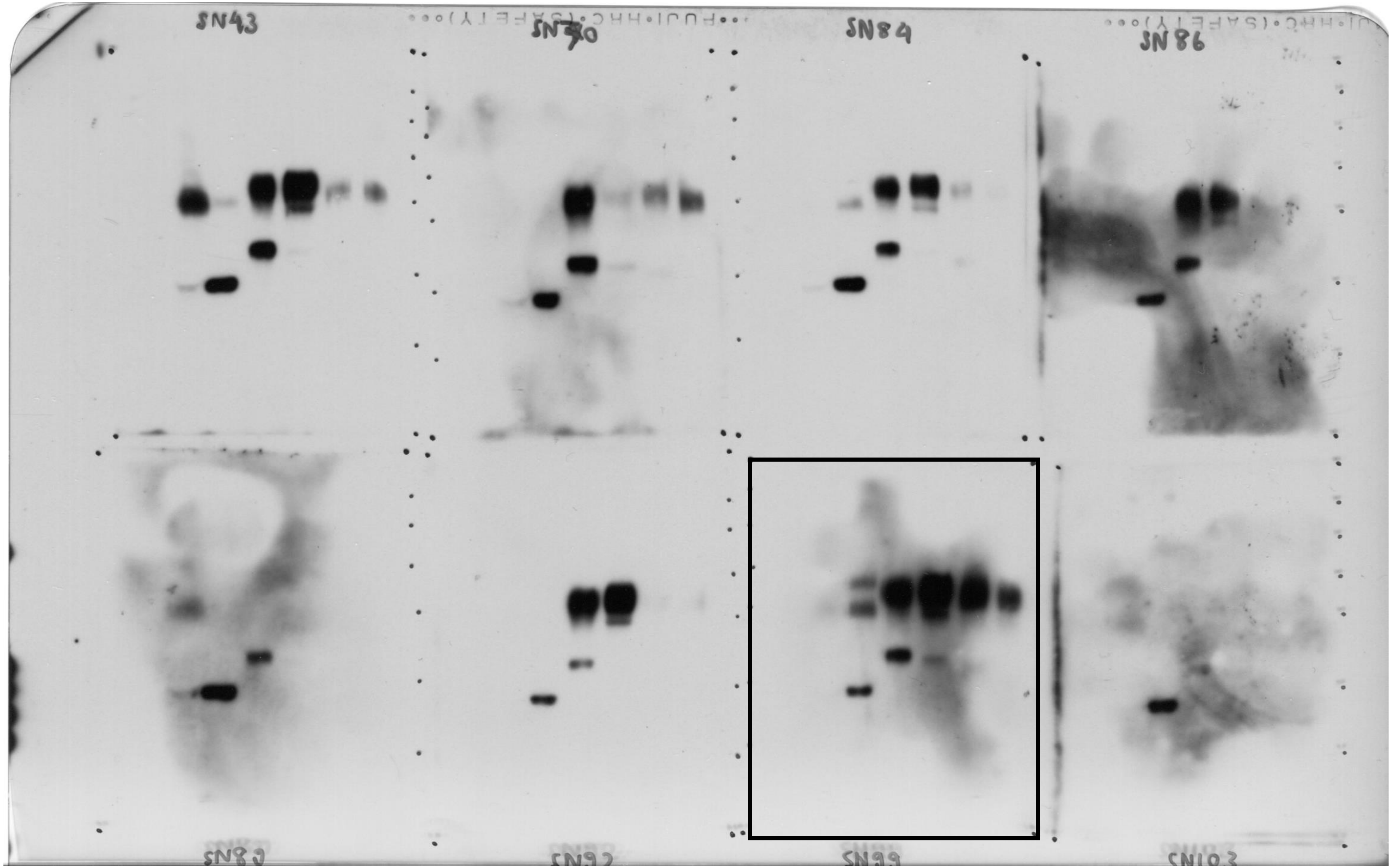

Figure S50

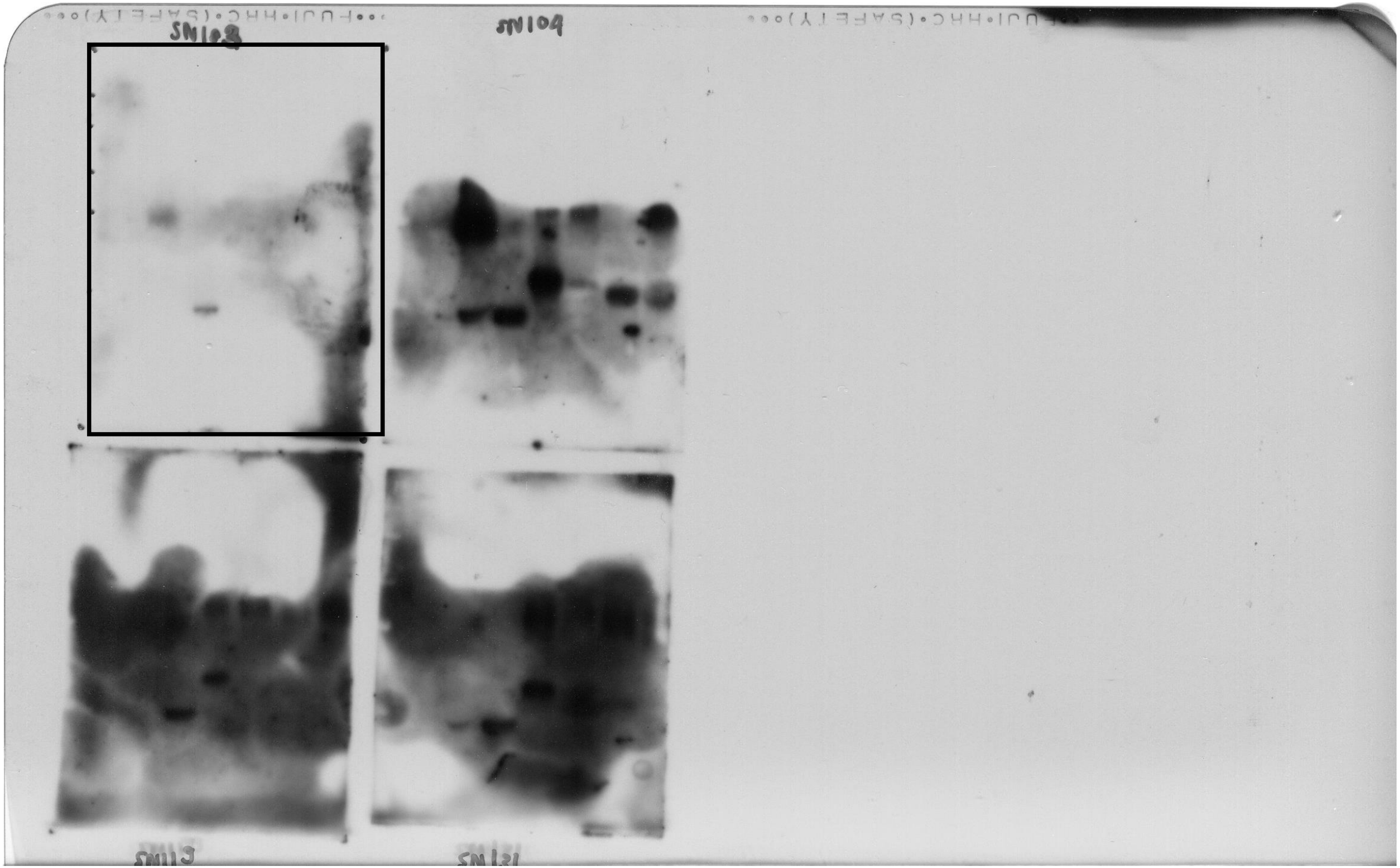

Figure S51

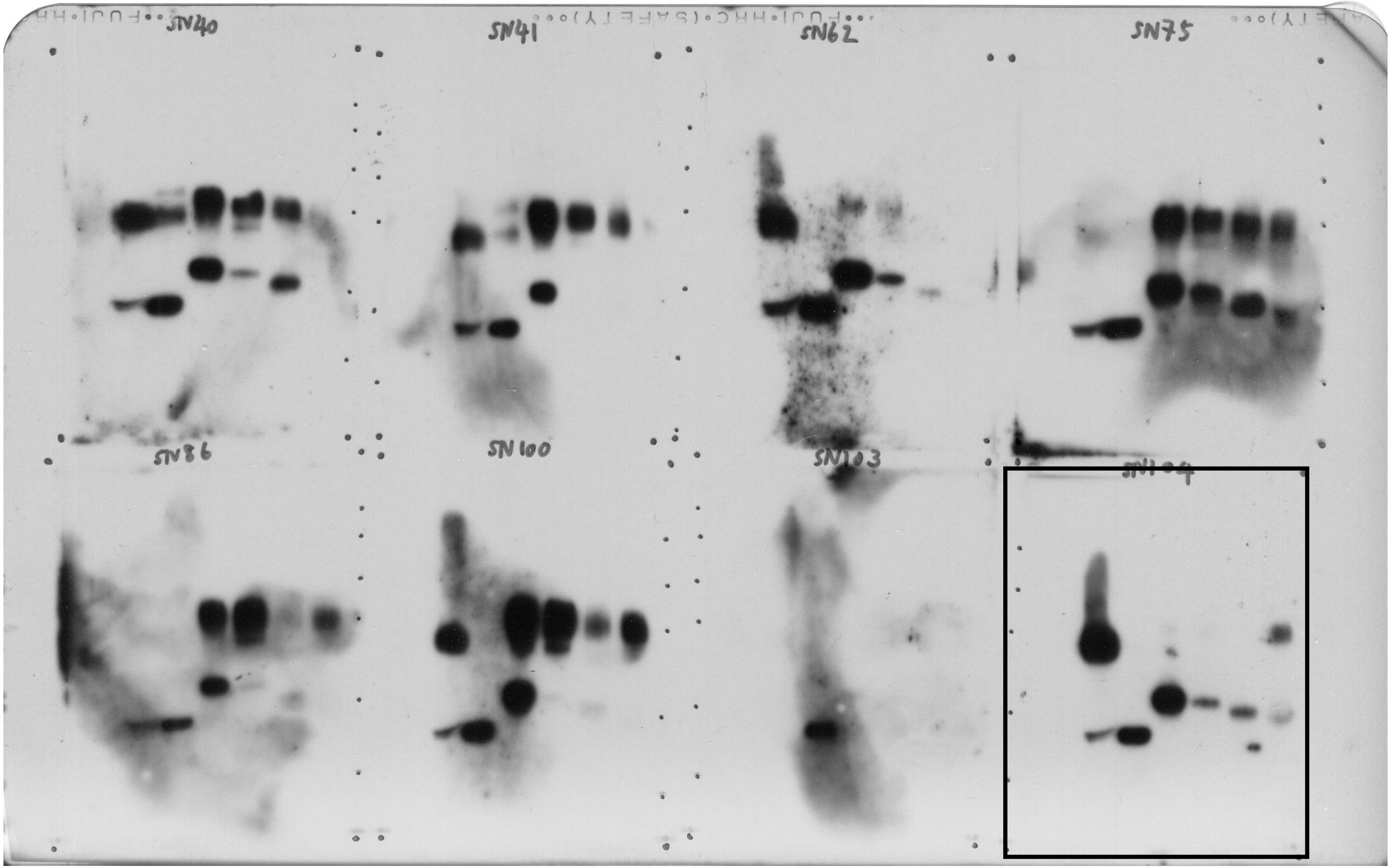

Figure S52

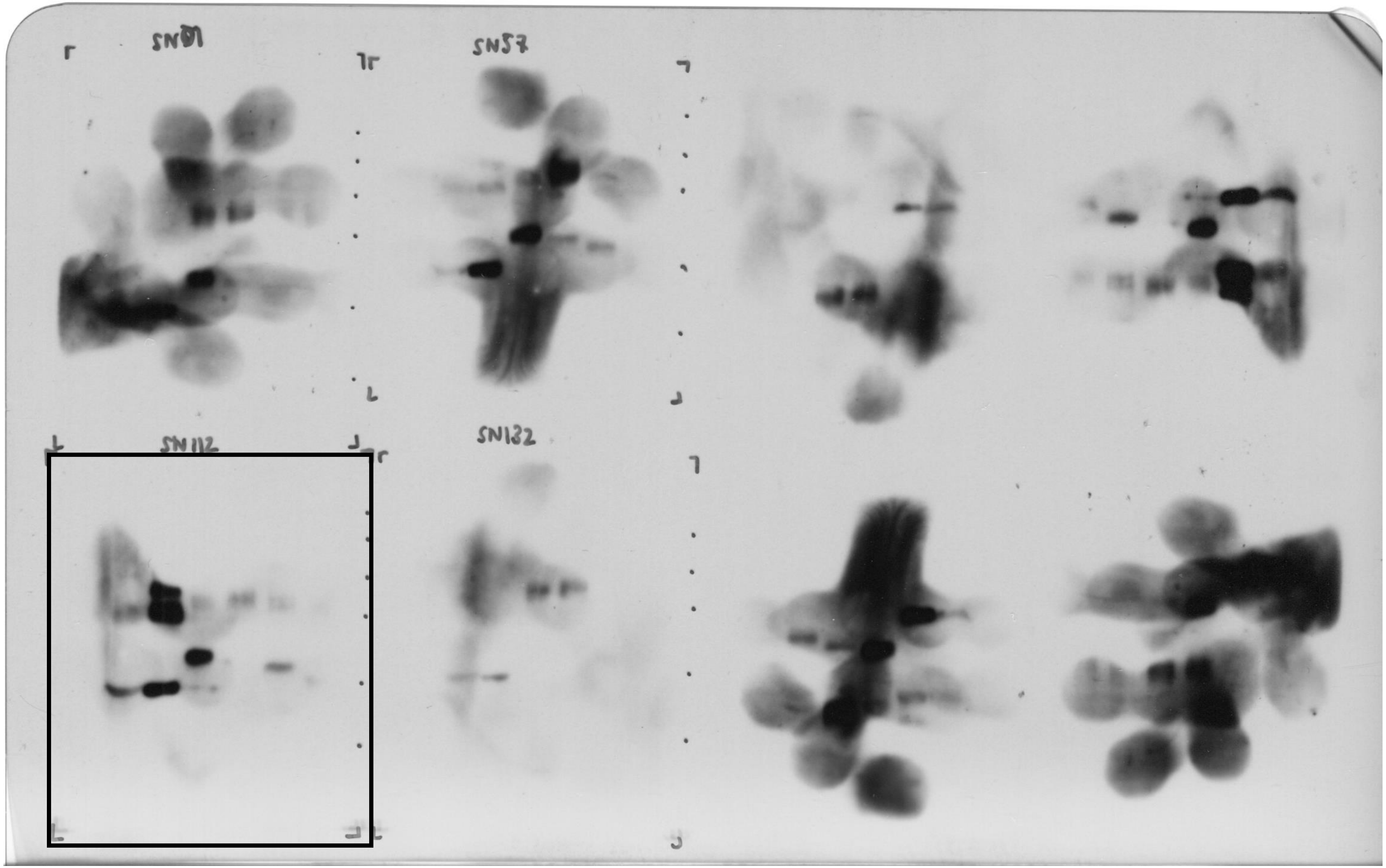

Figure S53

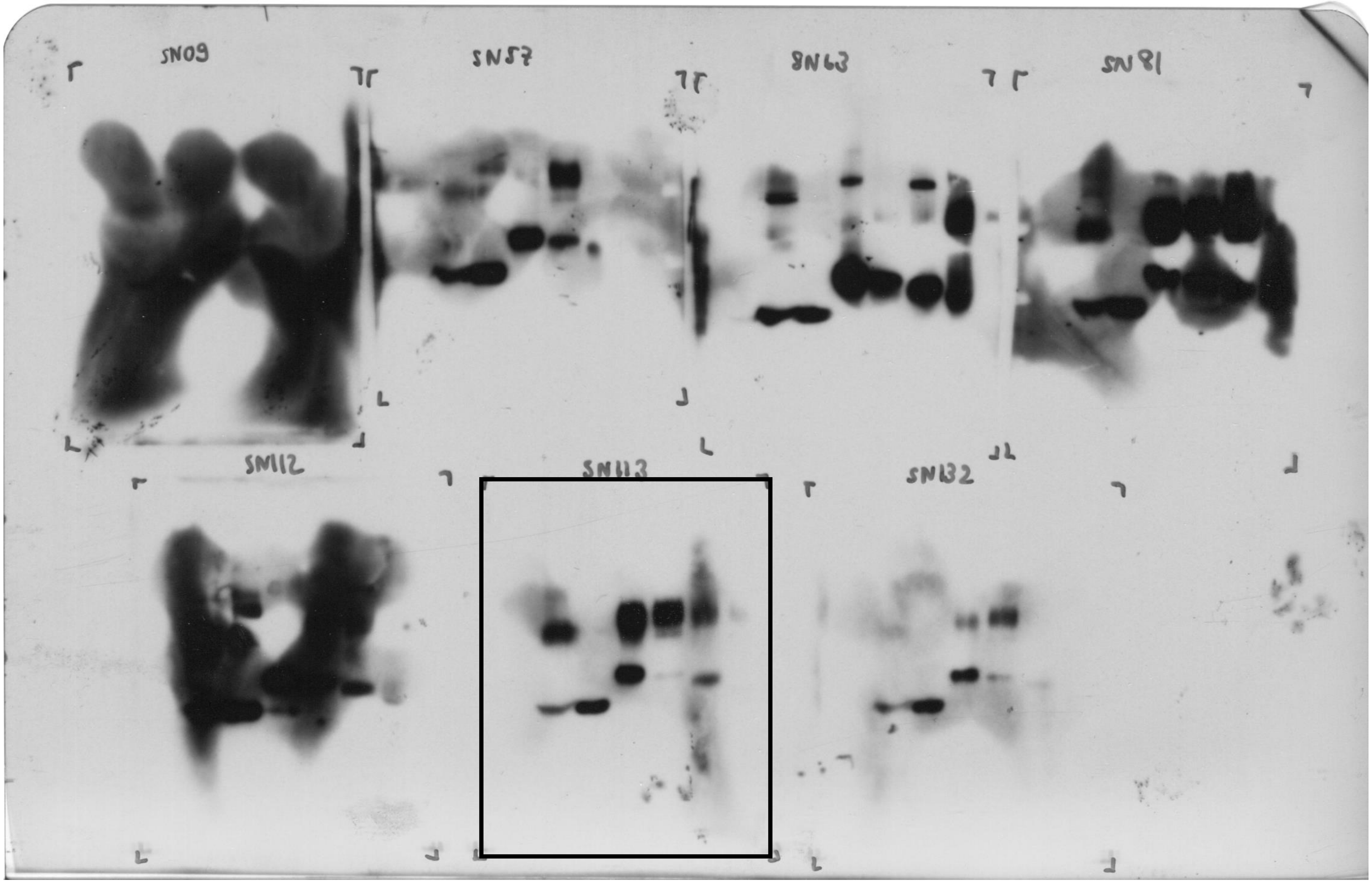

Figure S54

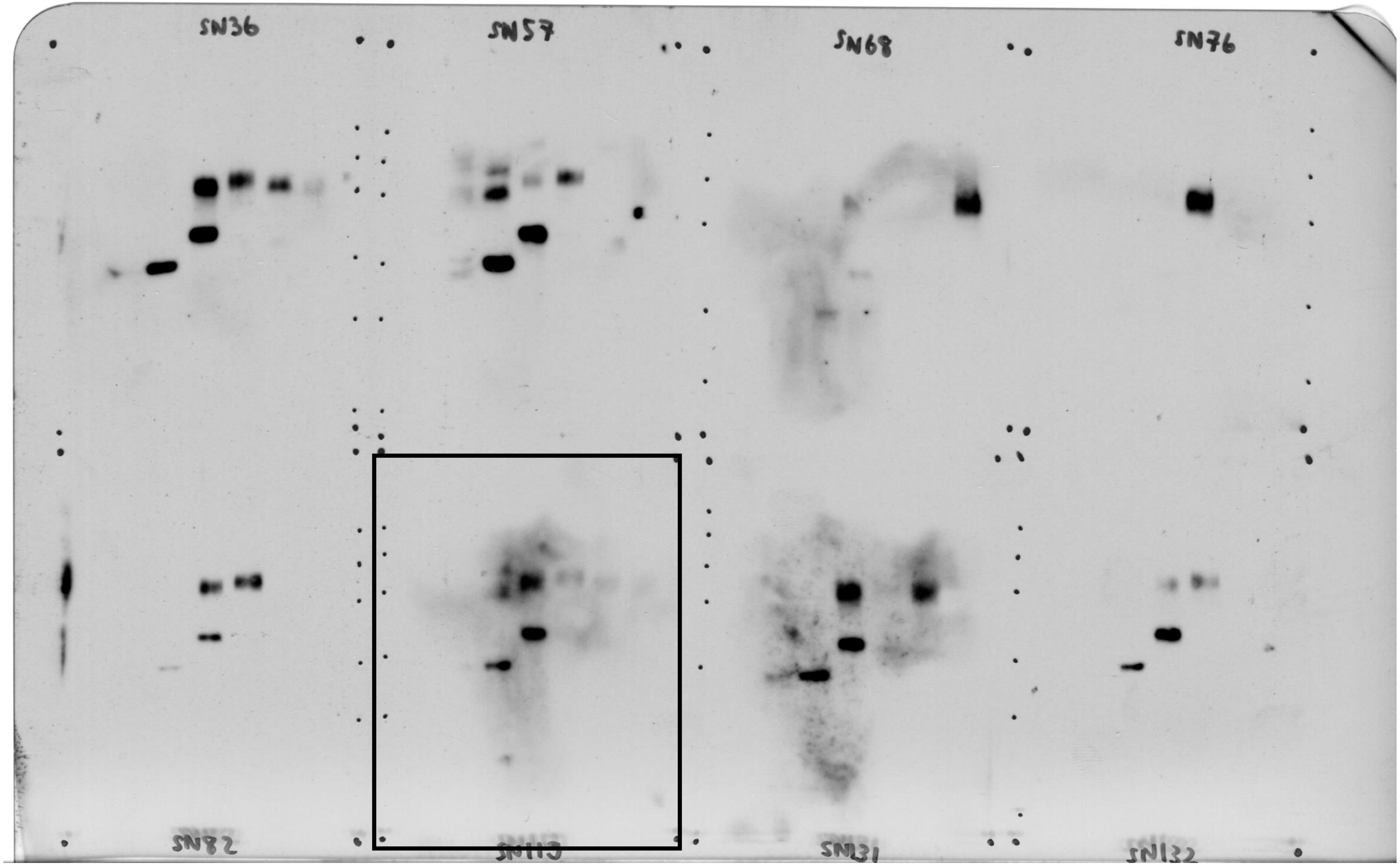

Figure S55

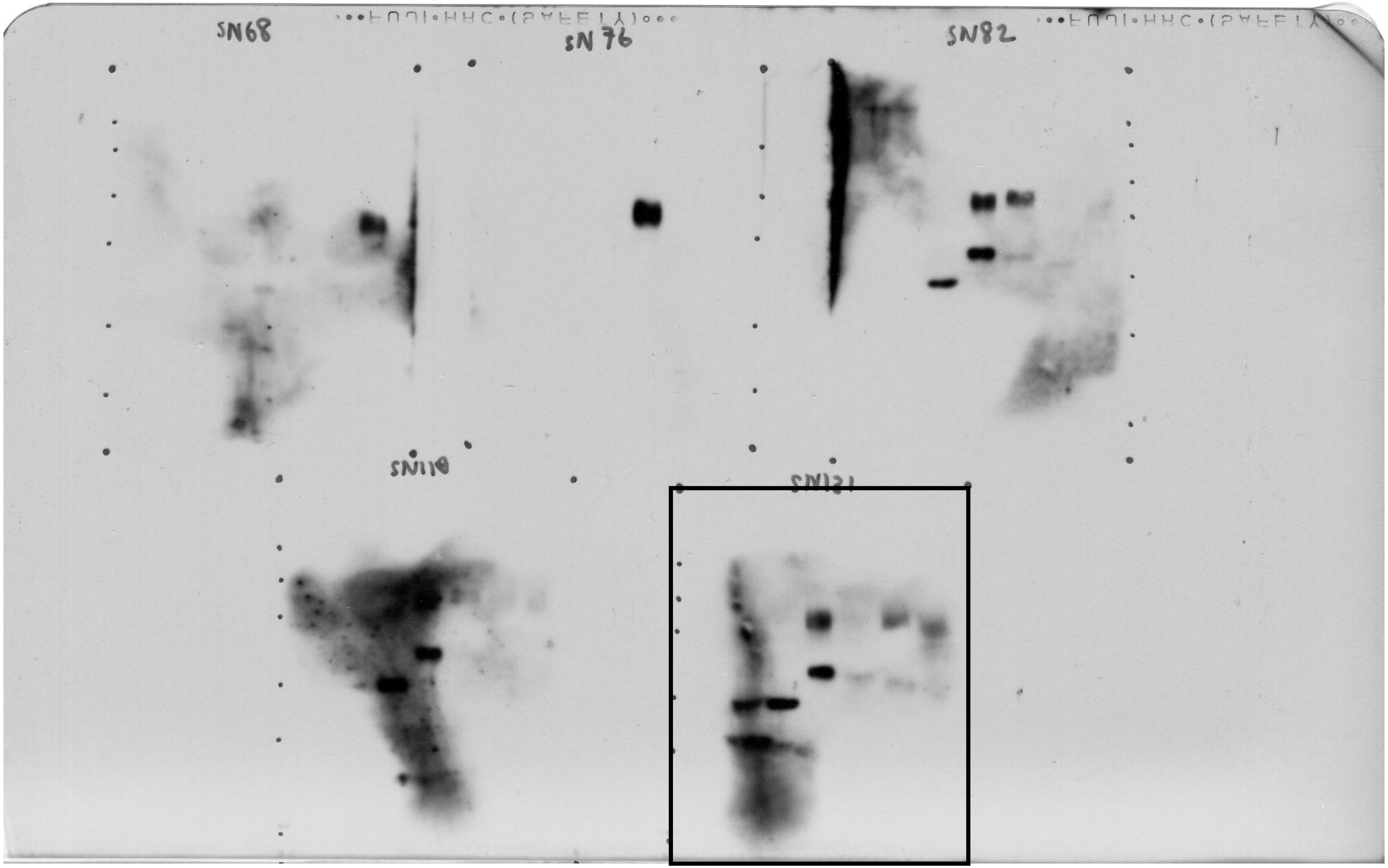

Figure S56

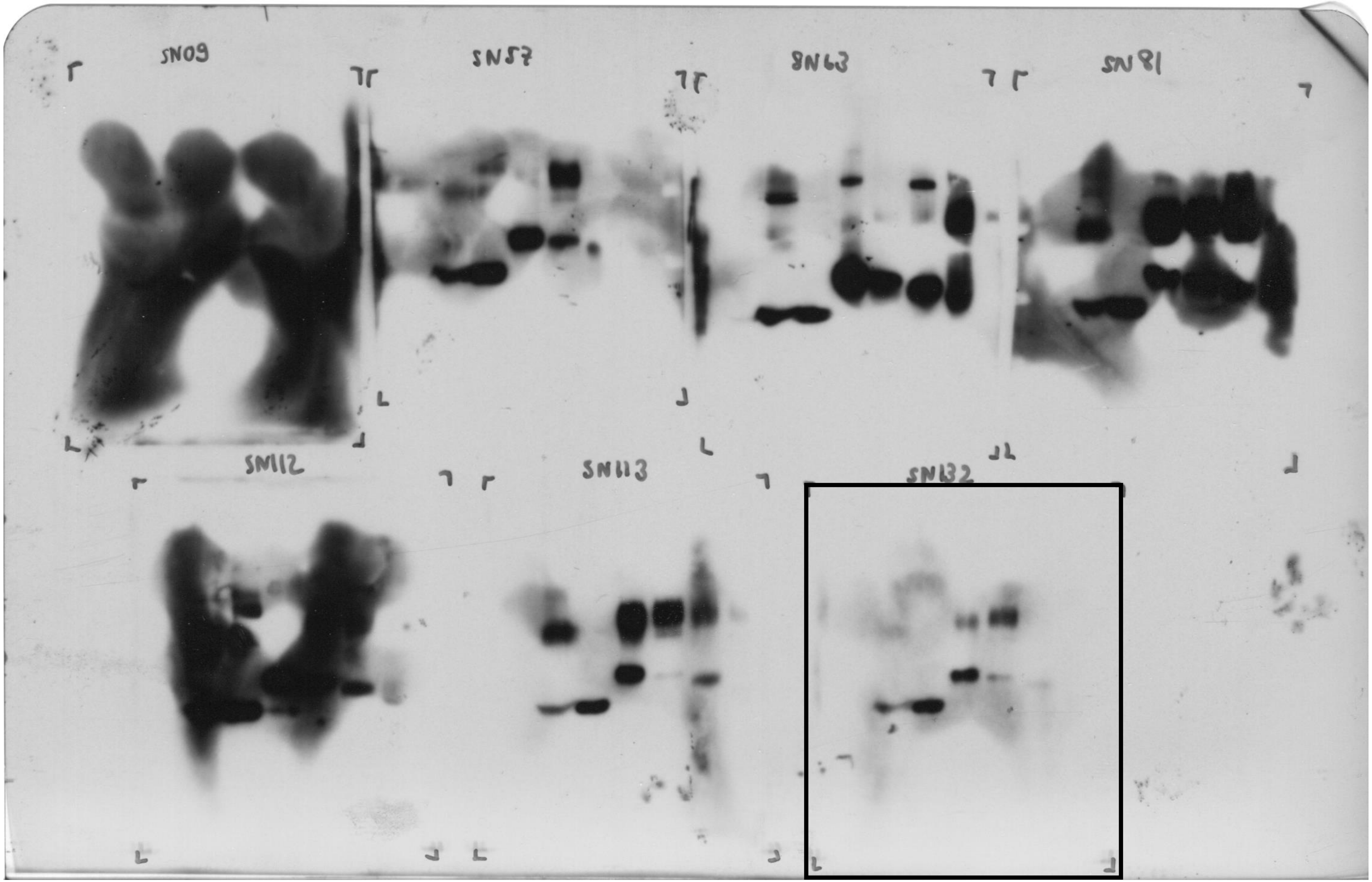

Figure S57

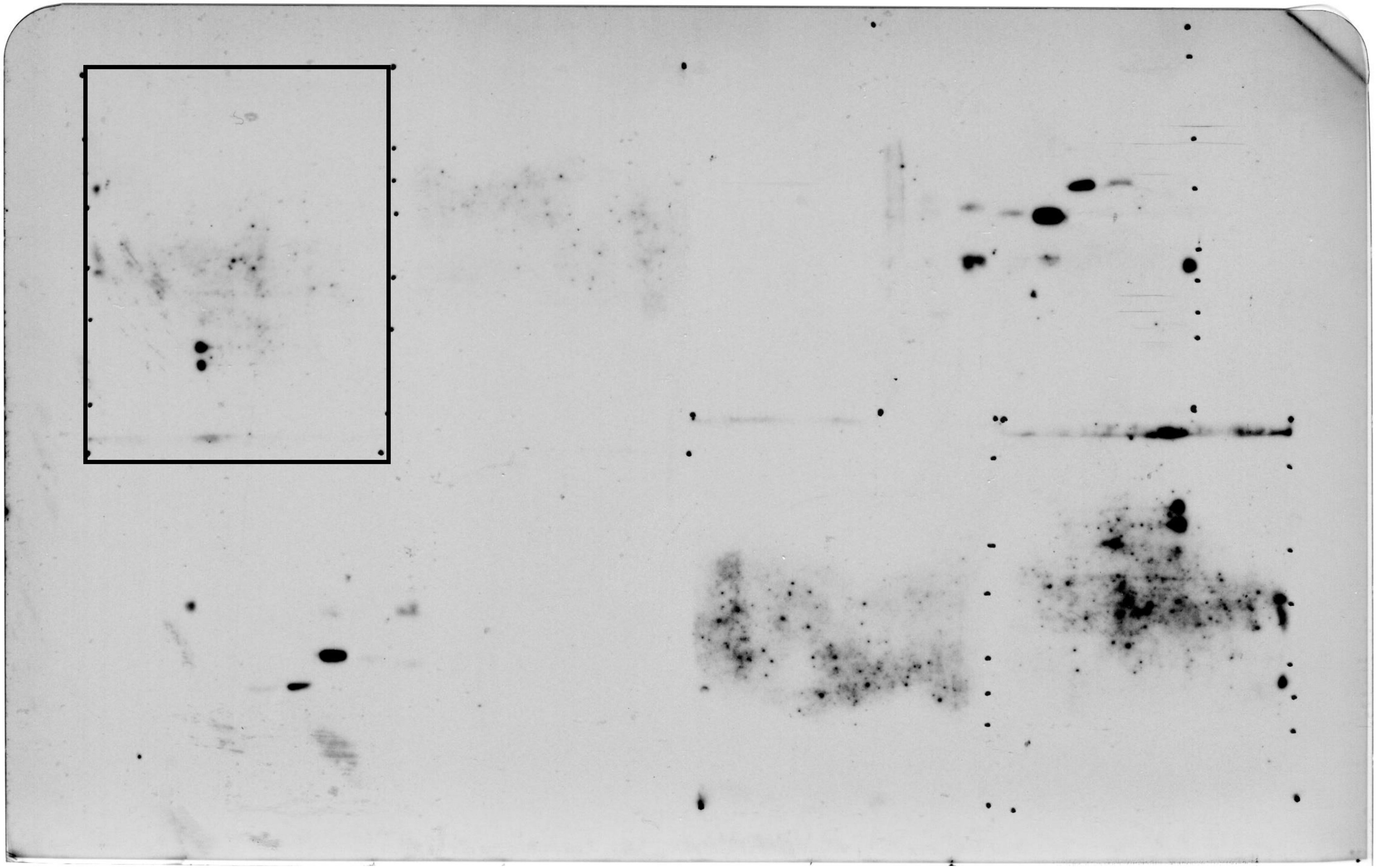

Figure S58

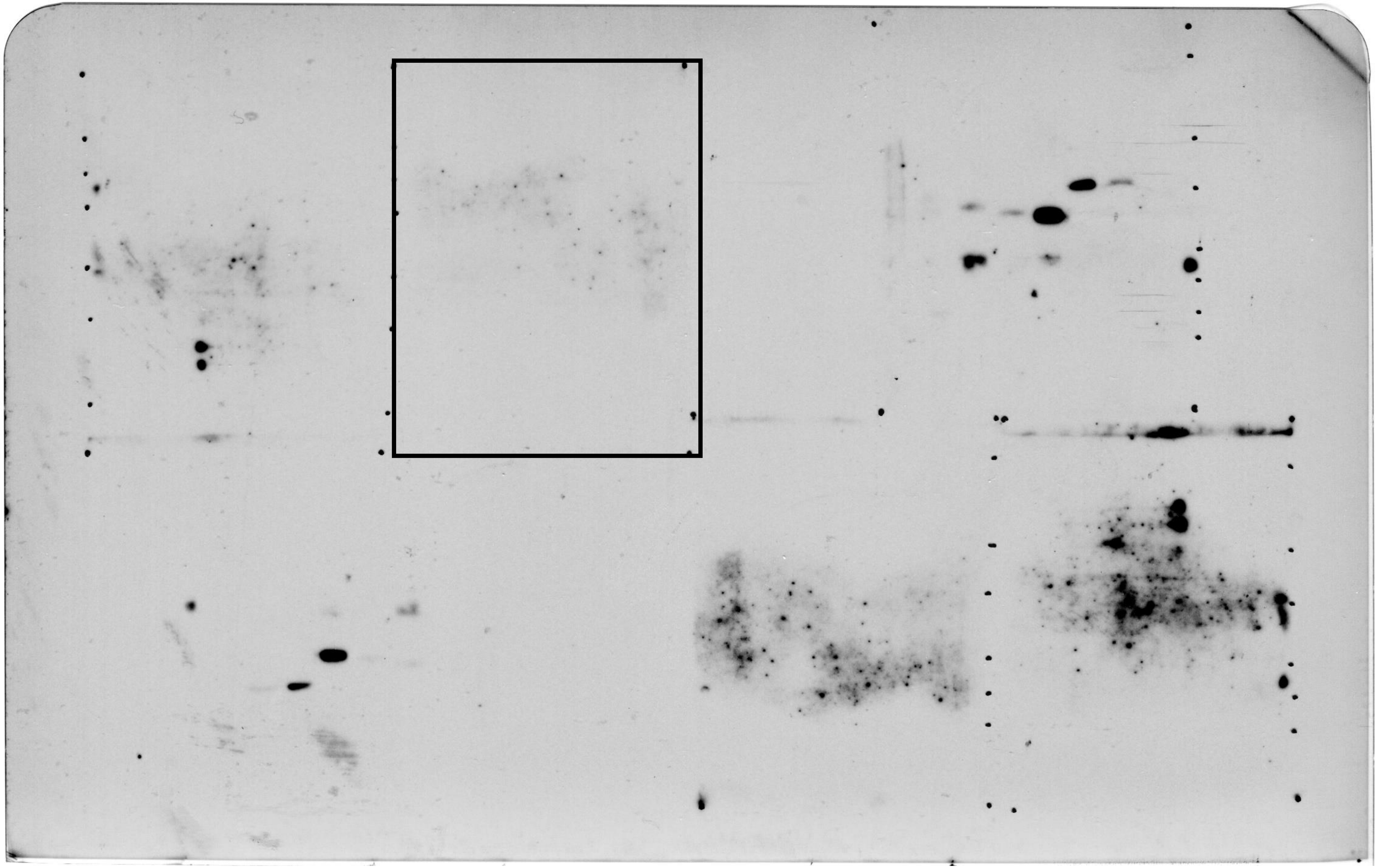

Figure S59

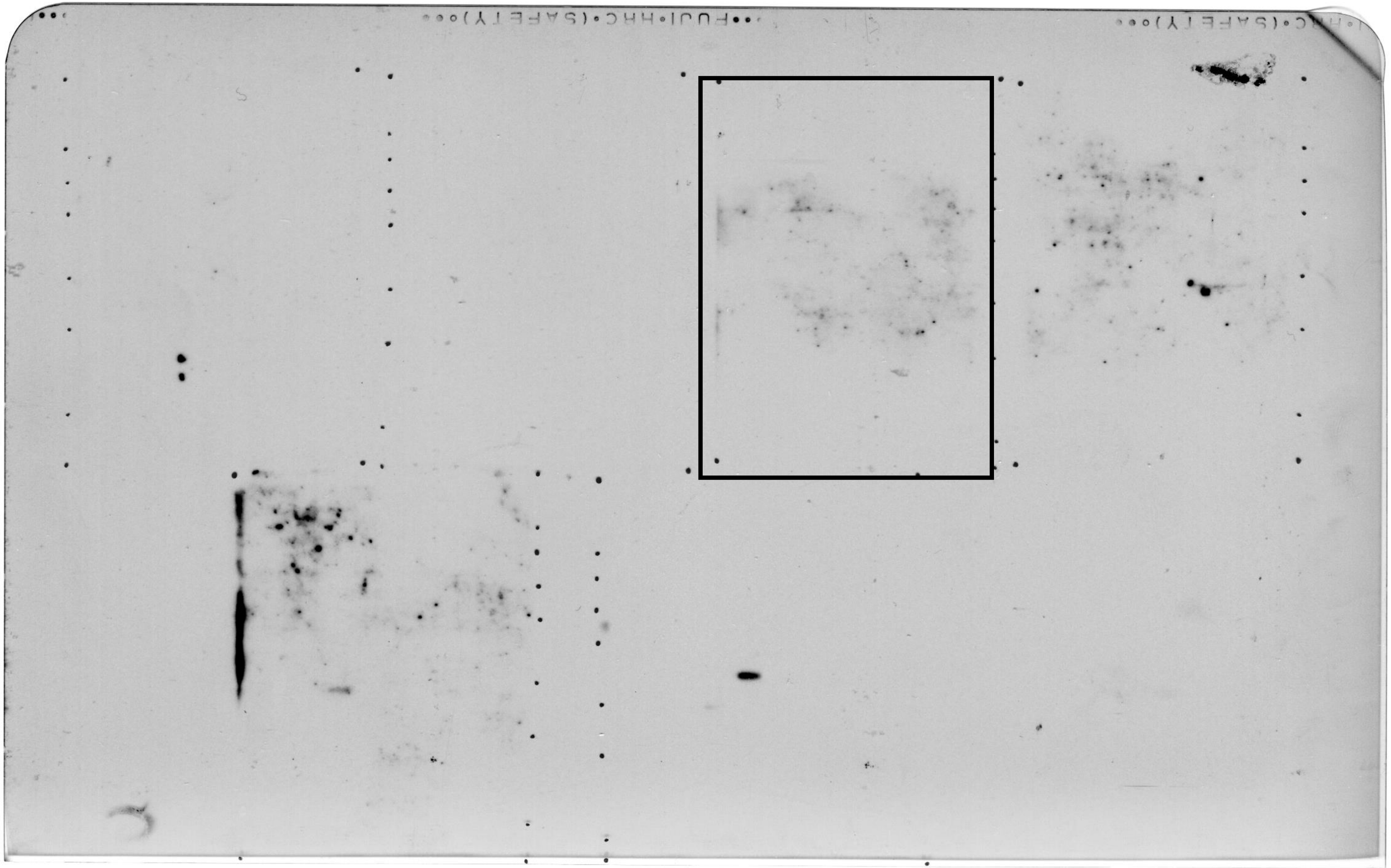

Figure S60

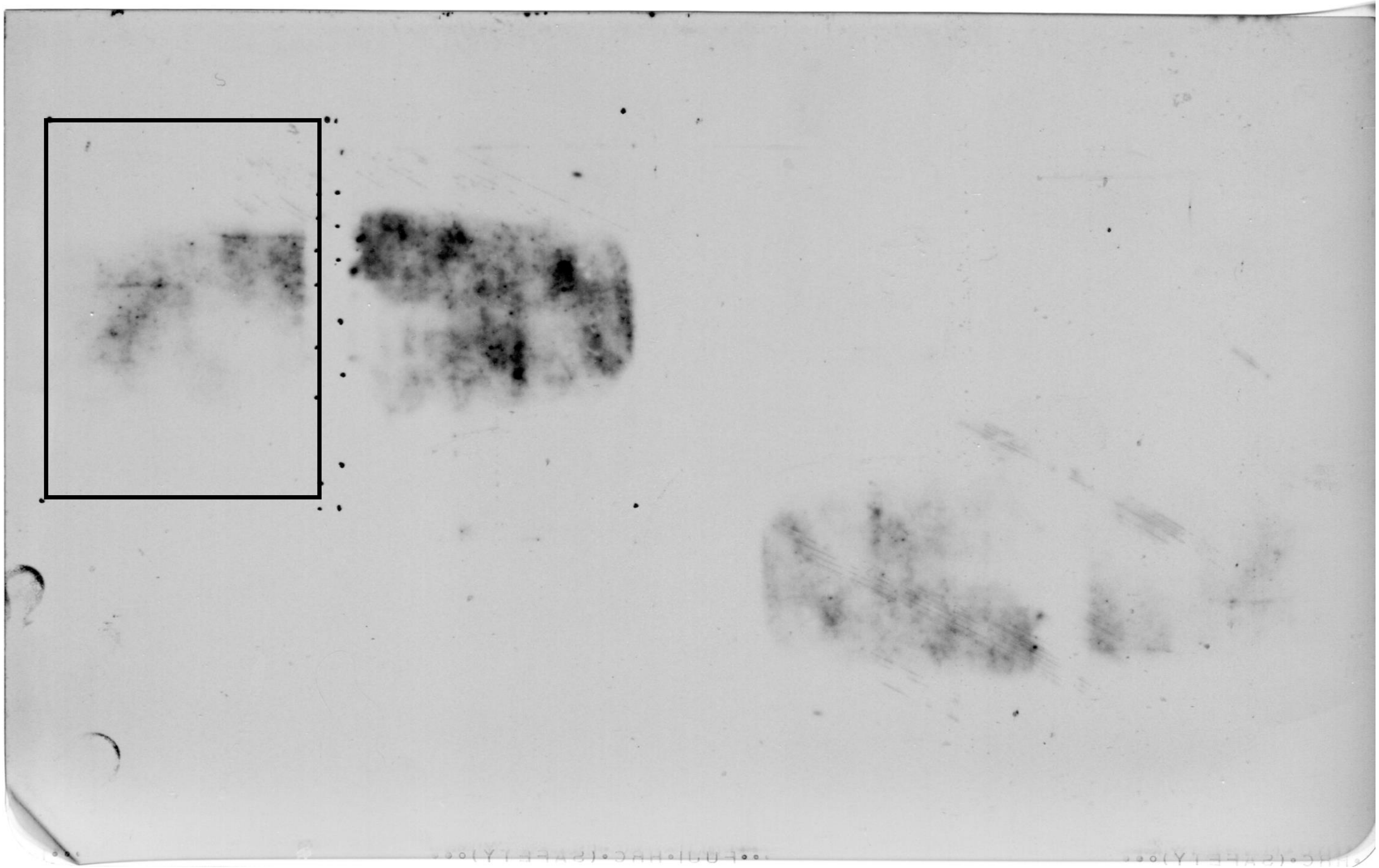

Supplement: Supplementary file 2 — Uncropped western blots [file 41598_2019_49569_MOESM2_ESM.pdf]
